# Loss of E3 Ubiquitin Ligase RINES via CpG Methylation Relieves Suppression of STAT3 and MYC, Facilitating Multiple Tumorigeneses

**DOI:** 10.1002/advs.202523684

**Published:** 2026-07-14

**Authors:** Lili Li, Ka Man Ng, Xingsheng Shu, Xiaoxue Chai, Kai Yau Wong, Gopesh Srivastava, Anthony T.C. Chan, Wai Yee Chan, Qian Tao

**Affiliations:** ^1^ Cancer Epigenetics Laboratory Department of Clinical Oncology State Key Laboratory of Translational Oncology and Sir YK Pao Center for Cancer The Chinese University of Hong Kong Hong Kong SAR China; ^2^ School of Medicine Shenzhen University Health Science Center Guangdong China; ^3^ Department of Pathology The University of Hong Kong Hong Kong SAR China; ^4^ School of Biomedical Sciences The Chinese University of Hong Kong Hong Kong SAR China; ^5^ Johns Hopkins Singapore Johns Hopkins School of Medicine Baltimore Maryland USA; ^6^ Hainan Academy of Medical Sciences Hainan Medical University Haikou China

**Keywords:** E3 ligase, methylation, RINES, stemness, tumor suppressor, ubiquitination

## Abstract

Dysregulated protein modifications alter the stability and activation status of key regulatory proteins and contribute to tumorigenesis. RING finger (RNF) proteins as E3 ubiquitin ligases, play fundamental roles in diverse biological processes, including cancer cell stemness. Through cancer epigenomic profiling, we identify tumor‐specific promoter CpG methylation of the E3 ligase *RINES* across multiple common cancer types (esophageal, nasopharyngeal, colorectal, breast, lung, kidney, cervical, and liver), in addition to previously reported gastric cancer, which correlates with poor patient survival. We further find that RINES inhibits tumor cell growth in vivo and in vitro. Mechanistically, RINES physically interacts with STAT3 and MYC to promote their protein degradation via its E3 ubiquitin ligase activity. RING domain‐dependent ubiquitin–proteasome degradation of STAT3 and MYC is essential for RINES‐mediated suppression of cancer stemness. Consistently, RINES knockdown diminishes the ubiquitination of STAT3 and MYC and elevates their protein stability, which enhances cancer stem cell properties and promotes tumorigenesis. Thus, our study establishes RINES as a bona fide tumor suppressor that directly modulates STAT3 and MYC stability to restrict cancer cell stemness. *RINES* promoter methylation may serve as a promising epigenetic biomarker for multiple common cancers.

## Graphical abstract

Dysregulated protein modifications drive tumorigenesis. RINES, an E3 ubiquitin ligase, represses tumor cell proliferation and metastasis by facilitating RING domain‐dependent, ubiquitin–proteasome‐mediated degradation of STAT3 and MYC, which consequently restrains cancer stemness and oncogenic progression. Aberrant promoter CpG methylation of *RINES* in multiple human malignancies correlates with unfavorable patient survival outcomes and represents a promising diagnostic/prognostic biomarker.

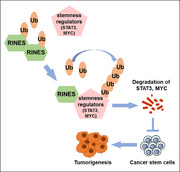

## 1 Introduction

Protein ubiquitination, a common post‐translational modification in eukaryotes, plays crucial roles in cancer development and progression [1]. This modification critically regulates multiple cancer hallmarks, including cell proliferation, apoptosis, metastasis, stemness, and metabolism [2, 3]. E3 ubiquitin ligases confer substrate specificity within the ubiquitin‐proteasome system (UPS) by directly binding to target proteins [4]. Recent advances in targeted protein degradation technologies, particularly molecular glues and proteolysis‐targeting chimeras (PROTACs), have enabled the targeting of previously undruggable proteins, highlighting significant therapeutic potential for cancer treatment [5, 6].

RING (Really Interesting New Gene) finger (RNF) proteins constitute the largest group of E3 ligases among the five subfamilies in the UPS [7, 8]. Over 300 RNF family members have been identified, most of which exert E3 ligase activity via single or multiple subunits [9, 10]. Acting as either oncoproteins (e.g., MDM2, SKP2) or tumor suppressors (e.g., BRCA1/RNF53, FBXW7, and VHL), RNF E3 ligases profoundly contribute to tumor pathogenesis by disrupting genomic integrity and cellular homeostasis [1, 2, 7]. Emerging evidence indicates that RNF E3 ligases play fundamental roles in maintaining cancer stemness properties, thereby driving tumor initiation, metastasis, and therapy resistance [11]. For instance, FBXW7 governs the balance between self‐renewal and quiescence in cancer stem cells by promoting Notch and ZMYND8 degradation [12]. As a critical stem cell‐related E3 ligase, RNF43 targets Wnt coreceptors for proteasomal degradation [13, 14], whereas BRCA1/RNF53 functions as a key stemness regulator in breast cancer [15, 16]. Numerous additional RNF proteins involved in the modulation of cellular stemness remain to be fully identified and functionally characterized.

In human cancers, genetic and/or epigenetic aberrations frequently lead to the inactivation of oncogenes and tumor suppressors, including multiple RNF E3 ligases [17, 18]. For example, *BRCA1/RNF53* mutations are strongly associated with increased breast cancer risk [19]; *HLTF/RNF80* inactivation through splicing mutations and promoter CpG methylation has been observed in multiple cancers [20, 21]. Notably, epigenetic alterations often precede genetic mutation accumulation during tumorigenesis, rendering them promising biomarkers for early cancer detection [22, 23, 24, 25, 26, 27]. These findings underscore the critical role of epigenetic disruption in modulating cancer‐related genes, especially RNF E3 ligases, during human carcinogenesis.

RINES, also known as ring finger protein 180, is an E3 ubiquitin ligase initially identified as a novel RING finger protein via screening for binding partners of the zinc finger transcription factor Zic2 [28]. Mouse Rines interacts with Zic2 via its basic coiled‐coil domain, promoting its ubiquitination and subsequent proteasomal degradation [28]. Human RINES shares high homology with mouse Rines and has been characterized as a tumor suppressor by regulating cell proliferation and metastasis in gastric cancer [29, 30, 31]. Notably, promoter CpG methylation of *RINES* exhibits higher sensitivity for early gastric cancer detection than conventional tumor markers, including AFP, CEA, CA199, CA125, and CA724 [32, 33, 34]. Moreover, the combined promoter methylation of *RINES* and *SEPTIN9* represents a promising plasma biomarker for early gastric cancer diagnosis [35]. However, the functional roles of RINES in other common carcinomas, such as esophageal and nasopharyngeal cancer, as well as its potential regulatory effects on cancer stemness during tumorigenesis, remain largely uncharacterized.

Through a genome‐wide CpG methylome study, we identified aberrant *RINES* promoter methylation across multiple cancer types, including esophageal, colorectal, breast, lung, renal, cervical, and hepatic cancers. We further validated that *RINES* is transcriptionally silenced by promoter CpG methylation in these carcinomas, and its downregulation correlates with poor survival, suggesting its biomarker potential. RINES acts as a broad tumor suppressor by restricting tumor metastasis and cancer stemness. RINES downregulation inversely correlates with the protein abundance of STAT3, MYC, and MDM2. RINES physically interacts with STAT3 and MYC, promoting their ubiquitination and degradation. Collectively, our findings uncover a previously unrecognized mechanism whereby RINES represses tumor stemness and malignant progression by targeting and destabilizing the oncogenic STAT3 and MYC proteins.

## 2 Materials and Methods

### 2.1 Cell Lines, Tissue Samples and Reagents

A panel of tumor cell lines originating from esophageal, head and neck (nasopharyngeal), colorectal, gastric, breast, lung, kidney, cervix, and liver cancer cell lines (Supporting Information) were purchased from ATCC (Rockville, MD), DSMZ (Braunschweig, Germany), or obtained from our collaborators [36, 37]. Immortalized human normal epithelial cell lines were cultured in KSFM or DMEM medium as previously reported [36, 37]. DNMT1/DNMT3B double‐knockout (DKO) human colon cancer HCT116 cells were a generous gift from Prof. Bert Vogelstein (Johns Hopkins Medicine) [38]. All cell lines used in this study were mycoplasma‐ and cross‐contamination‐free. Tumor cell lines were maintained in RPMI1640 or DMEM (Invitrogen, Grand Island, NY, USA) supplemented with 10% fetal bovine serum (FBS). For epigenetic drug treatment assays, cells were first incubated with 10 µM 5′‐aza‐2′‐deoxycytidine (Aza; Sigma–Aldrich, St Louis, MO, USA) for 72 h with daily medium refreshment, followed by 16 h of exposure to 100 ng/mL trichostatin A (TSA; Sigma–Aldrich, St Louis, MO, USA) [39].

Genomic DNA samples from primary carcinomas, adjacent normal tissues, nasal swabs of NPC patients, and normal epithelial tissues were obtained as previously described [37, 39, 40, 41, 42, 43]. The study protocol was approved by the Ethics Committee of The Chinese University of Hong Kong (2011.188 and 2015.132). Commercially sourced total RNA from non‐diseased adult human and fetal tissues was acquired from BioChain (Newark, CA, USA). Tissue microarrays (TMAs), consisting of primary ESCC lesions, matched adjacent normal esophageal mucosa, and NPC specimens with complete accompanying clinical data, were obtained from Shanghai Outdo Biotech Company.

### 2.2 Public Databases for Gene Expression and CpG Methylome Analysis

For screening across the NCI‐60 tumor cell line panel, datasets were downloaded from the CellMiner website [44, 45]: whole transcriptome RNA‐Seq data (Dataset: RNA‐seq composite expression), CpG methylome data (Dataset: DNA methylation [Illumina 450K array]—Gene average), and ChIP‐seq data (Dataset: Protein—Histone H3K4me3 and H3K27me3 [TMM‐FPKM (log_2_) normalized]).

Additional raw expression and CpG methylome datasets were retrieved from either the TCGA Data Portal or the GEO database. Correlations between *RINES* expression and DNA methylation were analyzed via the DNMIVD database. Associations between *RINES* methylation levels and overall survival of cancer patients were analyzed using MethSurv. Differential gene expression was evaluated with the UALCAN database [46] and the Human Protein Atlas [47].

### 2.3 Establishment of Stable Cell Lines

Lentiviral plasmids were co‐transfected into HEK293T cells along with a packaging plasmid encoding Gag‐Pol, VSVg, and Rev expression cassettes. Viral supernatants were collected at 48 h post‐transfection. To generate stable cell lines, target cells were transduced with lentiviral supernatants diluted 1:1 in complete culture medium supplemented with 8 µg/mL polybrene. Stable clones were selected via continuous culture in medium containing 1–2 µg/mL puromycin for 7 days.

### 2.4 In Vivo Xenograft Models

Four‐week‐old female BALB/c nude mice were used for xenograft tumor assays. KYSE150 cells in logarithmic growth phase were harvested and resuspended in an appropriate volume of Hank's Balanced Salt Solution (HBSS). After random grouping, mice received subcutaneous injections of 0.2 mL cell suspension (2 × 10^7^cells/mL) adjacent to the dorsal side of the right forelimb. Tumor growth monitoring began on day 8 post‐injection and was performed every three days. All measurements followed institutional animal welfare guidelines to ensure maximal tumor diameter remained below 20 mm. Tumor volume was calculated using the formula V = 0.5 × L × W^2^, where L represents the longest diameter and W the shortest diameter. For in vivo bioluminescence imaging, mice were imaged with the IVIS Lumina LT system (PerkinElmer), and average radiant efficiency was quantified as [p/s/cm^2^/sr]/[µW/cm^2^] to assess photon emission. All animal experimental protocols were approved by the Institutional Animal Care and Use Committee of Shenzhen University (IACUC‐202300054).

### 2.5 Antibodies and Reagents

Detailed antibody information is listed in Table S3. All chemical reagents were obtained from commercial sources: 5‐aza‐2'‐deoxycytidine (Aza), trichostatin A (TSA), cycloheximide (CHX), polybrene and puromycin were purchased from Sigma–Aldrich (St. Louis, MO, USA); MG132 (HY‐13259) was obtained from MedChemExpress (Monmouth Junction, NJ, USA); and small interfering RNAs (siRNAs, SR317264) were purchased from OriGene (Rockville, MD, USA).

### 2.6 Tumor Spheroid Formation

Freshly isolated tumor cells were seeded in 24‐well ultra‐low attachment plates (Corning, New York) at a density of 5000 cells per well. Cells were cultured in sphere‐forming medium consisting of serum‐free DMEM/F‐12 supplemented with 1× B27 serum substitute, 20 ng/mL human recombinant epidermal growth factor, and 20 ng/mL basic fibroblast growth factor (Thermo Fisher Scientific, Waltham, MA) [48]. Plates were incubated at 37°C with 5% CO_2_ for 5–7 days. Spheroids were subsequently quantified under an optical microscope (Olympus, Tokyo, Japan).

### 2.7 Protein Ubiquitination Assay

Protein ubiquitination assay was performed as previously described [36, 49]. Briefly, tumor cells stably expressing RINES, isoform 2, or RING‐domain mutant were either directly lysed or transfected with His‐Ub, His‐Ub‐K48, GFP‐STAT3, or GFP‐MYC plasmids for 48 h. Before harvesting, cells were treated with 10 µM MG132 (Sigma–Aldrich, St. Louis, MO, USA) for 6 h. Endogenous or exogenous STAT3 and MYC were immunoprecipitated with the indicated antibodies, and their ubiquitination levels were determined via immunoblotting using anti‐Ub or anti‐His antibodies.

Ni‐NTA pull‐down assay was conducted as previously described [36, 50]. Cell lysates were affinity‐purified using Ni‐NTA agarose beads (#30210, Qiagen, Hilden, Germany), followed by immunoblot analysis using STAT3‐ and MYC‐specific antibodies.

In vitro ubiquitination assays were performed using a ubiquitination kit (#BML‐UW9920, Enzo Life Sciences, Farmingdale, NY, USA) according to the manufacturer's instructions. Briefly, each 50 µL reaction mixture contained 2.5 µM biotinylated ubiquitin, 100 nM E1, 2.5 µM E2 (UbcH6), 5 mM Mg‐ATP, 100 nM purified GST‐RINES, and 1 µM purified target protein: STAT3 (#TP315836, Origene, Rockville, MD, USA) or MYC (#TP760019, Origene, Rockville, MD, USA). Reactions were incubated at 37°C for 4 h, then analyzed by Western blot with Streptavidin‐HRP detection.

### 2.8 Statistical Analysis

All experiments were performed independently in triplicate unless otherwise specified. For TCGA and GSE dataset analyses, sample sizes differed across cancer types/subtypes and are listed in each figure. Data are presented as mean ± standard deviation (SD). Two independent groups were compared via the two‑tailed unpaired Student's *t*‐test, whereas paired specimens (e.g., tumor vs. matched adjacent normal tissue) were analyzed with the two‑tailed paired Student's *t*‐test. Non‐parametric tests (Mann‐Whitney *U* test) were used for two‐group comparisons. One‐way ANOVA with Tukey's post hoc test was applied for comparisons among three or more groups when appropriate. Statistical significance was defined as *p* < 0.05. All statistical analyses were conducted using GraphPad Prism 8 (GraphPad Software, San Diego, CA, USA). Additional analyses were performed using R software (version 4.2.1) for TCGA data processing and correlation analyses.

Additional materials and methods are detailed in the Supporting Information.

## 3 Results

### 3.1 CpG Methylome Identifies *RINES* as a Methylated Target in Common Cancers

**FIGURE 1:**
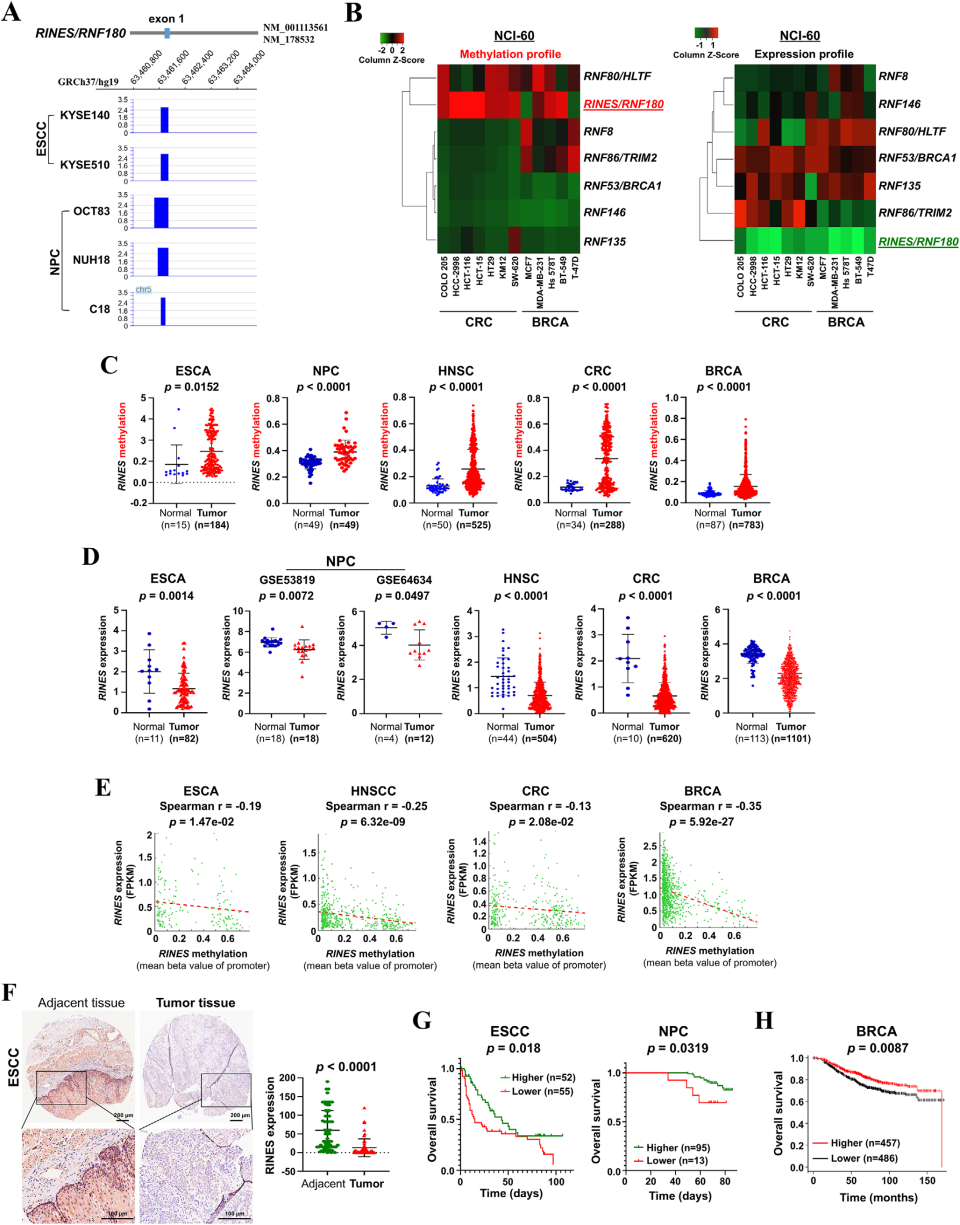
Epigenomic identification of *RINES* as a methylated cancer gene frequently downregulated in common cancers. (A) CpG methylome analysis by MeDIP‐chip revealed signal enrichment in the promoter CpG island (CGI) of *RINES/RNF180* in ESCC cell lines and NPC tumors. Positive signal peaks (marked in blue) and *RINES* exon 1 are highlighted. ESCC, esophageal squamous cell carcinoma; NPC, nasopharyngeal carcinoma. (B) Hierarchical clustering of methylome and expression profiles of various *RNF* genes using the NCI‐60 panel of CRC and breast cancer cell lines. Z‐score values for expression from DNA methylation levels by 450K methylation microarray (left) and RNA‐seq (right) are displayed, with green indicating low values and red indicating high values. The dendrogram illustrates hierarchical clustering based on RNA‐Seq and methylome data for each cell line. CRC, colorectal cancer; BRCA, breast cancer. (C) Mean β‐values of 11 CpG sites within the *RINES* CGI were plotted for primary tumors of ESCA, NPC, HNSCC, CRC, and BRCA, along with adjacent normal samples from the TCGA or GSE database (GSE52086 and GSE62336). Methylation differences were analyzed using a nonparametric *t*‐test. ESCA, esophageal cancer; HNSC, head and neck squamous cell carcinoma. (D) *RINES* expression levels across different tumor types were assessed using TCGA or GEO databases. Expression differences were analyzed using a nonparametric *t*‐test. (E) Analysis of the TCGA dataset indicated a negative correlation between *RINES* mRNA expression and methylation levels in ESCA, HNSCC, CRC, and BRCA tumors, with each green dot representing an individual clinical sample. Spearman correlation coefficient analysis was used. (F) Representative IHC images show RINES staining in ESCC tissues compared to adjacent normal tissues, revealing reduced RINES expression in ESCC samples. The *p*‐value was determined by an unpaired test. Scale bar: 200 or 100 µM. (G) Kaplan–Meier analysis of overall survival in patients with ESCC and NPC was conducted based on RINES protein expression scores using the log‐rank test. (H) Kaplan–Meier analysis showing overall survival of breast cancer patients stratified by *RINES* mRNA expression levels, assessed using the log‐rank test.

We first investigated CpG methylation of the *RINES* promoter in common cancers. Analysis of our MeDIP‐based CpG methylome data revealed significant methylation enrichment in the *RINES* promoter CpG island (CGI) in two ESCC cell lines (KYSE140, KYSE510) and three NPC tumor samples (OCT83, NUH18, C18) (Figure 1A). We next compared the methylation profiles of *RINES* with those of other RNF family members using the NCI‐60 methylome database (Figure 1B, Figure S1A) and observed a markedly higher methylation frequency of *RINES* in multiple colon and breast tumor cell lines relative to other RNF genes (Figure 1B). We further validated *RINES* methylation levels in clinical tumor datasets from TCGA and GSE cohorts. Notably, *RINES* methylation levels were significantly elevated in esophageal, nasopharyngeal, head and neck, colon, and breast tumors compared with adjacent normal tissues (Figure 1C). These results suggest that *RINES* is a frequently methylated target gene in common cancers.

### 3.2 RINES Downregulation is Linked to Poor Survival and Disease Progression in Patients

Since promoter CpG methylation typically induces gene silencing, we next examined *RINES* mRNA expression across multiple public tissue databases. We found that *RINES* mRNA is ubiquitously expressed in normal human tissues and fetal tissues (Figure S1B), but is significantly downregulated in tumor tissues of the esophagus, nasopharynx, head and neck, colorectal, and breast relative to matched normal tissues (Figure 1D). Moreover, *RINES* mRNA expression exhibits a significant inverse correlation with its promoter methylation level (Figure 1E), suggesting that *RINES/RNF180* methylation mediates its transcriptional silencing in multiple common cancers.

To evaluate RINES protein expression in tumor tissues, we performed immunohistochemistry (IHC) on a tissue microarray containing 72 paired ESCC tumor and adjacent normal tissue samples, along with 36 additional ESCC tumor specimens (Figure 1F). RINES protein expression levels were markedly decreased in ESCC tumor tissues compared with matched normal controls (Figure 1F). Clinically, reduced RINES expression correlated with poorer overall survival in patients with ESCC, NPC, and breast cancer (Figure 1G,H). In the NPC cohort, RINES downregulation was significantly associated with advanced TNM stage (*p* = 0.00371), and the metastasis (M) stage served as an independent prognostic factor for patient survival (Table S1). Furthermore, decreased *RINES* expression was correlated with advanced pathological stages in colorectal and breast cancers, including the triple‐negative breast cancer (TNBC) subtype (Figure S1C,D). These findings suggest that RINES suppression contributes to the malignant progression of multiple human carcinomas.

### 3.3 *RINES* Methylation is Linked to Reduced Survival Rates in Cancer Patients

**FIGURE 2:**
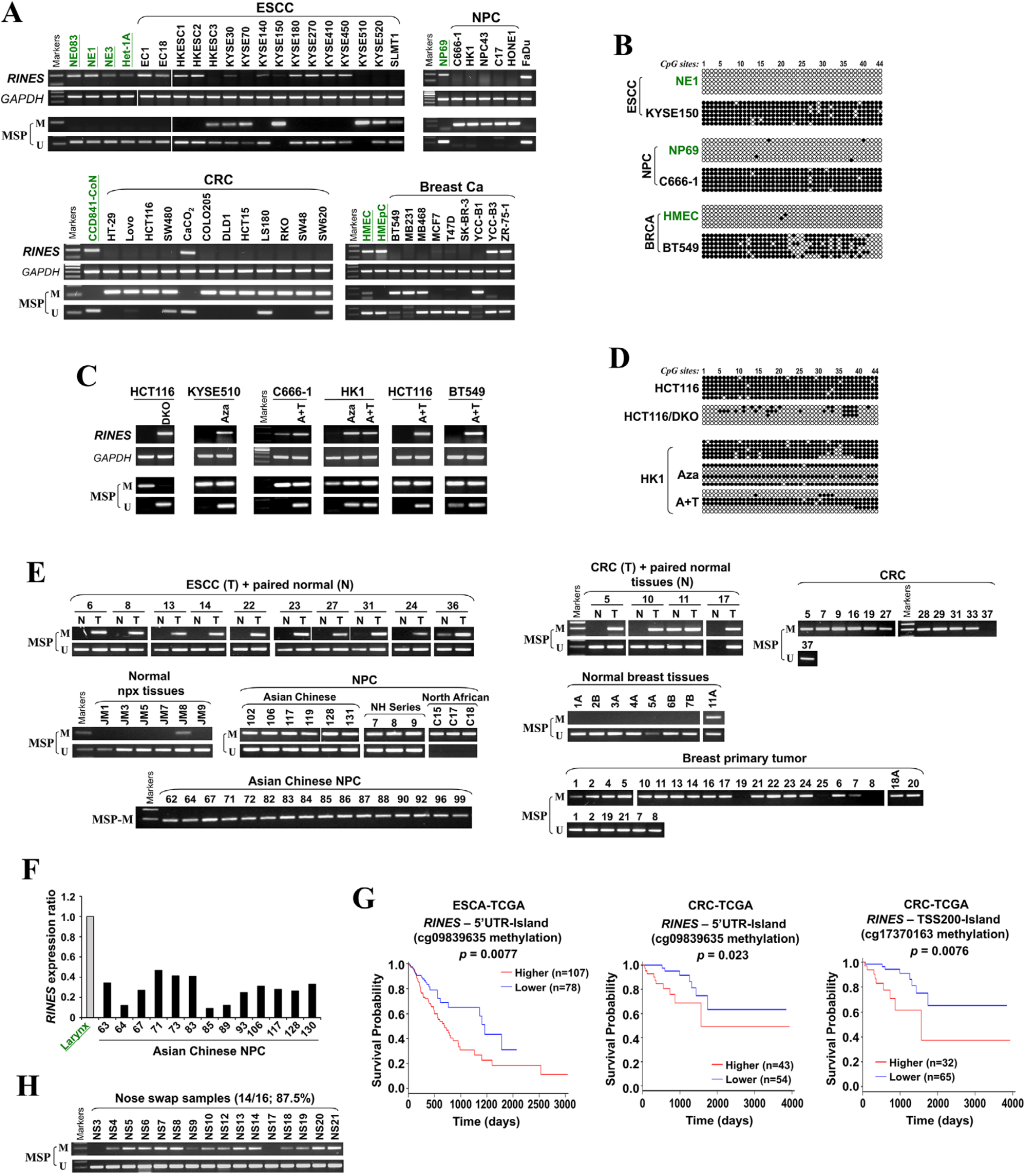
Silencing of *RINES* via promoter CpG methylation in common tumor cell lines and tissues. (A) Downregulation and methylation of *RINES* were assessed in ESCC, NPC, CRC, and breast cancer cell lines, as well as normal epithelial cell lines, using semi‐quantitative PCR or methylation‐specific PCR (MSP). ESCC, esophageal squamous cell carcinoma; NPC, nasopharyngeal carcinoma; CRC, colorectal cancer; Ca, cancer. M: methylated; U: unmethylated. (B) High‐resolution mapping of the methylation status of 44 CpG sites in the *RINES* promoter was performed using BGS in representative tumor and normal epithelial cell lines. Each allele of the *RINES* promoter is displayed as a single row, with filled circles indicating methylated CpG sites and open circles indicating unmethylated sites. BRCA, breast cancer. (C) Both pharmacological and genetic demethylation treatments restored *RINES* expression in methylated and silenced tumor cell lines. Genetic double knockout (DKO) of both DNMT1 and DNMT3B in HCT116 cells resulted in significant promoter demethylation and *RINES* re‐expression. Treatment with the demethylating agent Aza, alone or in combination with TSA, also restored *RINES* expression in tumor cell lines, along with demethylation. A, aza; T, TSA. (D) BGS analysis of *RINES* methylation in representative tumor and normal cell lines was performed following genetic or pharmacological demethylation. (E) *RINES* promoter methylation was frequently detected in primary tumors but was rare in adjacent normal tissues. T, tumor tissue; N, adjacent normal tissue; npx, nasopharynx. (F) Real‐time PCR revealed downregulation of *RINES* in representative primary NPC tumors, with expression levels normalized to those in normal laryngeal tissue. (G) Correlation between *RINES* methylation at specific CpG sites within its CGI and overall survival in esophageal and colorectal cancer patients (TCGA datasets) was assessed using Kaplan–Meier analysis. Patients were stratified into high (β > cut‐off) and low (β < cut‐off) methylation groups, with the optimal cut‐off determined by MethSurv. (H) *RINES* methylation was detected in nasal swab samples from NPC patients by MSP.

Given that the *RINES* promoter contains a typical CGI (Figure S2A), we analyzed *RINES* promoter methylation across multiple tumor cell lines and primary tumor samples. Semiquantitative RT‐PCR showed that *RINES* expression was silenced or reduced in several cancer cell lines, including ESCC, NPC, CRC, breast, cervical, and renal cancer, whereas robust *RINES* expression was retained in corresponding normal epithelial cell lines (Figure 2A, Figure S2B). Consistently, low *RINES* expression was also observed in tumor cells from the NCI60 and human protein atlas dataset (Figure 1B, Figure S3). We further interrogated mass spectrometry (MS)‐based proteomic datasets and found that the RINES protein was largely undetectable in most tumor cell lines originating from esophageal, head and neck, colorectal, and breast cancers (Figure S3).

Methylation‐specific PCR (MSP) confirmed frequent *RINES* promoter methylation in tumor cell lines of ESCC (7/16), NPC (5/5), CRC (11/12), and breast (4/9) (Figure 2A). *RINES* methylation was also detected in lung, cervical, and renal cancer cells, with relatively lower prevalence in liver cancer (Figure S2B), consistent with *RINES* silencing. In contrast, normal epithelial cell lines remained largely unmethylated, with only minimal methylation observed in HMEC cells (Figure 2A). Further bisulfite genome sequencing (BGS) analysis of *RINES* promoter methylation validated the MSP‐based methylation results (Figure 2B).

Consistent with the epigenetic regulatory mechanism, robust *RINES* expression was fully restored in DNMT1/DNMT3B double‐knockout HCT116 colon cancer cells (DKO) (Figure 2C, Figure S2C), accompanied by complete demethylation of the *RINES* promoter (Figure 2C,D). These genetic knockout results strongly indicate that promoter methylation is responsible for *RINES* transcriptional silencing. Furthermore, pharmacological inhibition of DNA methylation using the DNMT inhibitor Aza alone or combined with the HDAC inhibitor TSA significantly restored *RINES* expression and promoter demethylation in tumor cell lines (Figure 2C). BGS analysis further verified the loss of *RINES* promoter methylation following genetic or pharmacological demethylation interventions (Figure 2D). To exclude genomic deletion as an alternative mechanism of *RINES* downregulation, we performed multiplex differential genomic DNA PCR. No genomic deletion of the *RINES* locus was detected in *RINES*‐silenced tumor cell lines (Figure S2D), confirming that promoter CpG methylation serves as the predominant mechanism driving *RINES* epigenetic silencing in cancer cells.

We next validated the tumor‐specific methylation pattern of *RINES* in primary clinical specimens. Frequent *RINES* promoter methylation was detected in paired ESCC and colorectal tumors, as well as in independent cohorts of primary NPC (34/34), colorectal (10/11), and breast cancer (18/22) tumors (Figure 2E). In contrast, paired adjacent normal tissues and normal nasopharyngeal/mammary tissues exhibited negligible *RINES* methylation, further verifying its tumor‐specific methylation (Figure 2E). Reduced endogenous *RINES* expression in primary NPC tumors was also validated via quantitative real‐time PCR (Figure 2F). Clinically, elevated *RINES* promoter methylation levels were significantly associated with inferior survival outcomes in patients with esophageal and colorectal cancers (Figure 2G). Notably, *RINES* promoter methylation was detectable in 87.5% of nasopharyngeal swab samples from NPC patients (Figure 2H), highlighting its promising potential as a non‐invasive epigenetic biomarker for early NPC detection.

### 3.4 RINES, but not Its Short Isoform Lacking the RING Domain, Possesses Tumor Suppressive Activity

**FIGURE 3:**
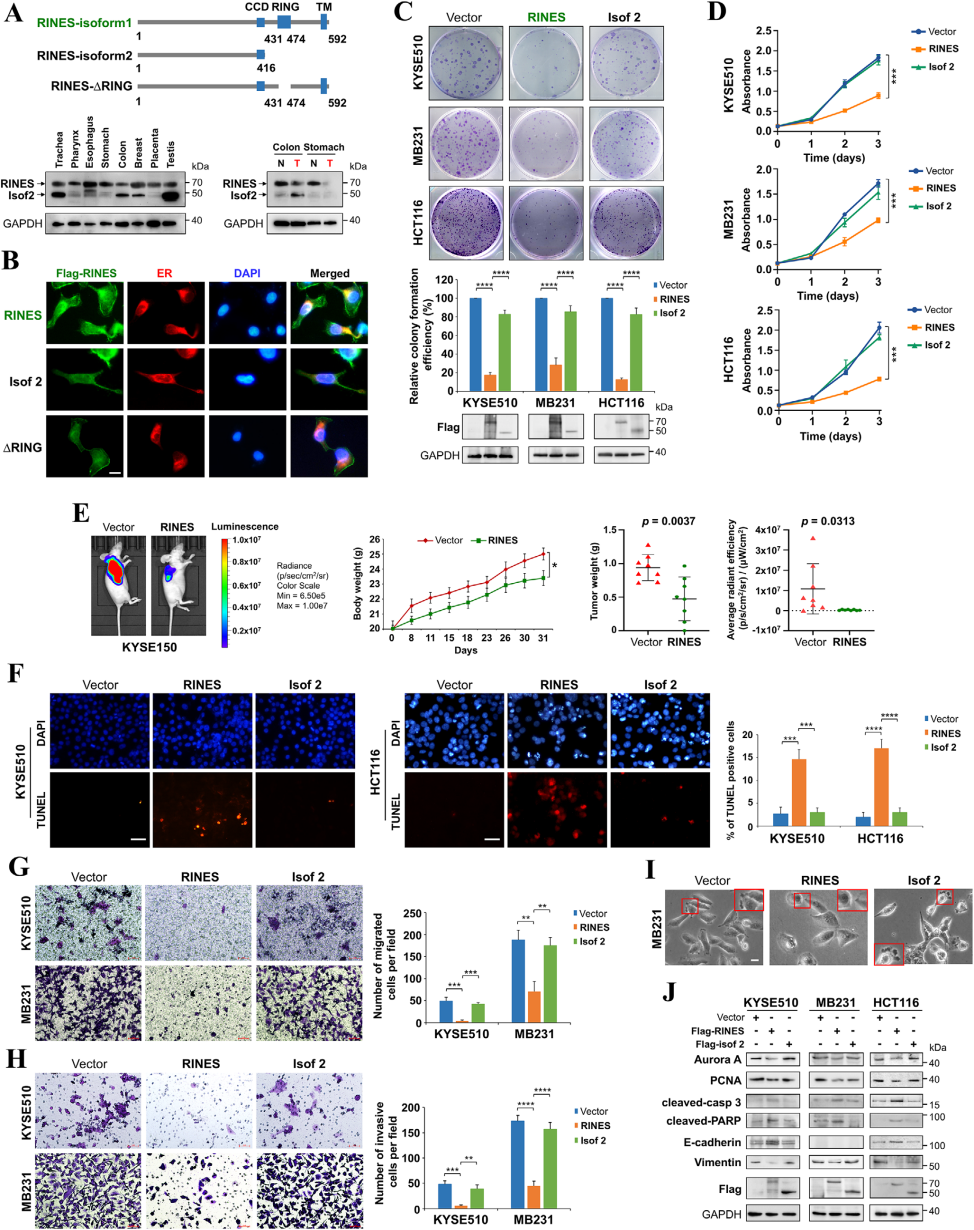
Tumor suppressive functions of RINES in carcinoma cells. (A) Schematic representation of full‐length RINES (isoform 1), RINES‐isoform 2, and the RING domain‐deleted mutant (RINES‐ΔRING). CCD, coiled‐coil domain; TM, transmembrane domain. RINES protein expression is shown in a normal tissue panel and in paired adjacent normal and tumor tissues. (B) Subcellular localization assay demonstrated co‐localization of RINES with the ER. HONE1 cells were co‐transfected with RINES, isoform 2, or RING‐mutant plasmids along with an ER‐specific marker (pDsRed‐ER). Nuclei were counterstained with DAPI (blue). Original magnification, 400×. Scale bar 100 µm. (C) Representative colony formation assay performed in monolayer cultures of tumor cells expressing RINES or isoform 2. Quantitative analysis of the colony numbers is shown, with data presented as mean ± SD from three independent experiments using one‐way ANOVA with Tukey's post hoc test. Colonies with more than 50 cells in empty vector‐transfected cells were designated as 100 for comparison. ^****^, *p* < 0.0001. (D) Cell viability of tumor cells expressing RINES or isoform 2 was assessed using the CCK‐8 assay. Data are presented as mean ± SD from three independent experiments using one‐way ANOVA with Tukey's post hoc test. ^***^, *p* < 0.001. (E) RINES suppresses tumor formation in the nude mice model of ESCC. KYSE150 cells infected with a CMV‐Firefly luciferase lentivirus vector encoding RINES or an empty vector were subcutaneously injected into BALB/c nude mice. Tumor progression was monitored by measuring body weight, tumor weight, and bioluminescence intensity. Data are presented as mean ± SD with representative images shown. Statistical significance was determined using an unpaired Student's *t*‐test. ^*^, *p* < 0.05. (F) TUNEL assay results in KYSE510 and HCT116 cells. Red fluorescence signals indicate TUNEL‐positive (apoptotic) cells. Data are presented as mean ± SD of three independent experiments using one‐way ANOVA with Tukey's post hoc test and representative data are shown. Scale bar, 50 µm. (G, H) Matrigel chamber assays evaluating migration (G) and invasion (H) of tumor cells expressing RINES or isoform 2. Data are represented as mean ± SD of three independent experiments by one‐way ANOVA with Tukey's post hoc test, and representative data are shown. Scale bar, 50 µm. (I) RINES, but not isoform 2, inhibits lamellipodia formation in MB231 cells. Scale bar 100 µm. (J) Western blot detection of markers for proliferation (Aurora A, PCNA), apoptosis (cleaved caspase‐3, cleaved PARP), and EMT (E‐cadherin, vimentin) in carcinoma cell lines.

Frequent silencing of *RINES* via promoter methylation in human cancers indicates its potential function as a tumor suppressor. RINES (isoform 1) has a unique domain architecture consisting of a RING finger domain, a coiled‐coil domain (CCD), and a transmembrane domain (TM), whereas isoform 2 lacks both the RING domain and the TM domain (Figure 3A). Amino acid sequence alignment and phylogenetic analysis further demonstrated that RINES is highly conserved across evolution (Figure S4). We examined RINES protein expression in a panel of normal tissues and found that RINES was abundantly expressed in all tissues tested, including trachea, pharynx, esophagus, stomach, colon, and breast, whereas isoform 2 was predominantly detected in the trachea, colon, breast, and testis. In paired tumor tissues of CRC and gastric cancer, RINES expression was markedly downregulated in tumor tissues relative to matched adjacent normal tissues (Figure 3A).

To investigate the biological functions of RINES, we constructed expression plasmids encoding full‐length RINES, isoform 2, and a RING domain deletion mutant (RINES‐∆RING). Immunofluorescence staining showed that full‐length RINES primarily localized to the cytoplasm. Both RINES and its mutant variants displayed similar membrane distribution, with only subtle differences in staining intensity (Figure 3B). Subsequent organelle colocalization assays revealed that RINES colocalized with endoplasmic reticulum (ER) markers, while isoform 2 and the RING‐deletion mutant exhibited substantially reduced ER association (Figure 3B). These results suggest that RINES participates in ER‐associated degradation (ERAD)‐mediated ubiquitination, likely through its RING domain.

Ectopic RINES expression in cell lines was comparable to endogenous levels but notably lower than its expression in normal brain tissue (Figure S5A), indicating that our overexpression system achieved moderate, physiologically relevant protein abundance. We next investigated the tumor suppressive role of RINES in carcinoma cells. Colony formation assays showed that ectopic expression of full‐length RINES profoundly inhibited colony formation in ESCC, NPC, CRC, and breast cancer cells (Figure 3C, Figure S5B). In contrast, cells expressing RING domain‐deficient isoform 2 exhibited only approximately 20% growth inhibition relative to cells expressing full‐length RINES (Figure 3C). Furthermore, full‐length RINES significantly suppressed the proliferation of KYSE510, MB231, and HCT116 cells compared to control cells, while isoform 2 failed to produce such an inhibitory effect (Figure 3D). In vivo experiments using an ESCC xenograft nude mouse model further verified that RINES overexpression suppressed tumor growth, as evidenced by reduced tumor weight, decreased fluorescence signal intensity in KYSE150 xenografts, and retarded tumor progression (Figure 3E). These results collectively establish RINES as a bona fide tumor suppressor.

To uncover the molecular mechanisms underlying the tumor‐suppressive effects of RINES, we performed terminal deoxyribonucleotide transferase‐mediated dUTP nick end labeling (TUNEL) assay and applied a pCaspase3‐sensor system. Compared with control cells, cells expressing full‐length RINES, but not isoform 2, displayed robust caspase activation and increased apoptotic cell numbers (Figure 3F, Figure S5C). Furthermore, RINES‐expressing cells showed markedly reduced migratory capacity (Figure 3G, Figure S5D) and invasiveness (Figure 3H, Figure S5E), accompanied by decreased lamellipodia formation (Figure 3I). At the molecular level, these phenotypic changes corresponded with downregulation of aurora A, proliferating cell nuclear antigen (PCNA), and vimentin, as well as upregulation of activated caspase and E‐cadherin (Figure 3J). Notably, none of these molecular and phenotypic alterations were observed in isoform 2‐expressing cells (Figure 3I,J). These results demonstrate that RINES, but not its isoform 2, functions as a tumor suppressor by inducing apoptosis and repressing clonogenic potential and metastatic capacity in carcinoma cells.

### 3.5 RINES Inhibits Cancer Stemness Properties by Downregulating Key Stemness Markers in a RING Domain‐Dependent Manner

**FIGURE 4:**
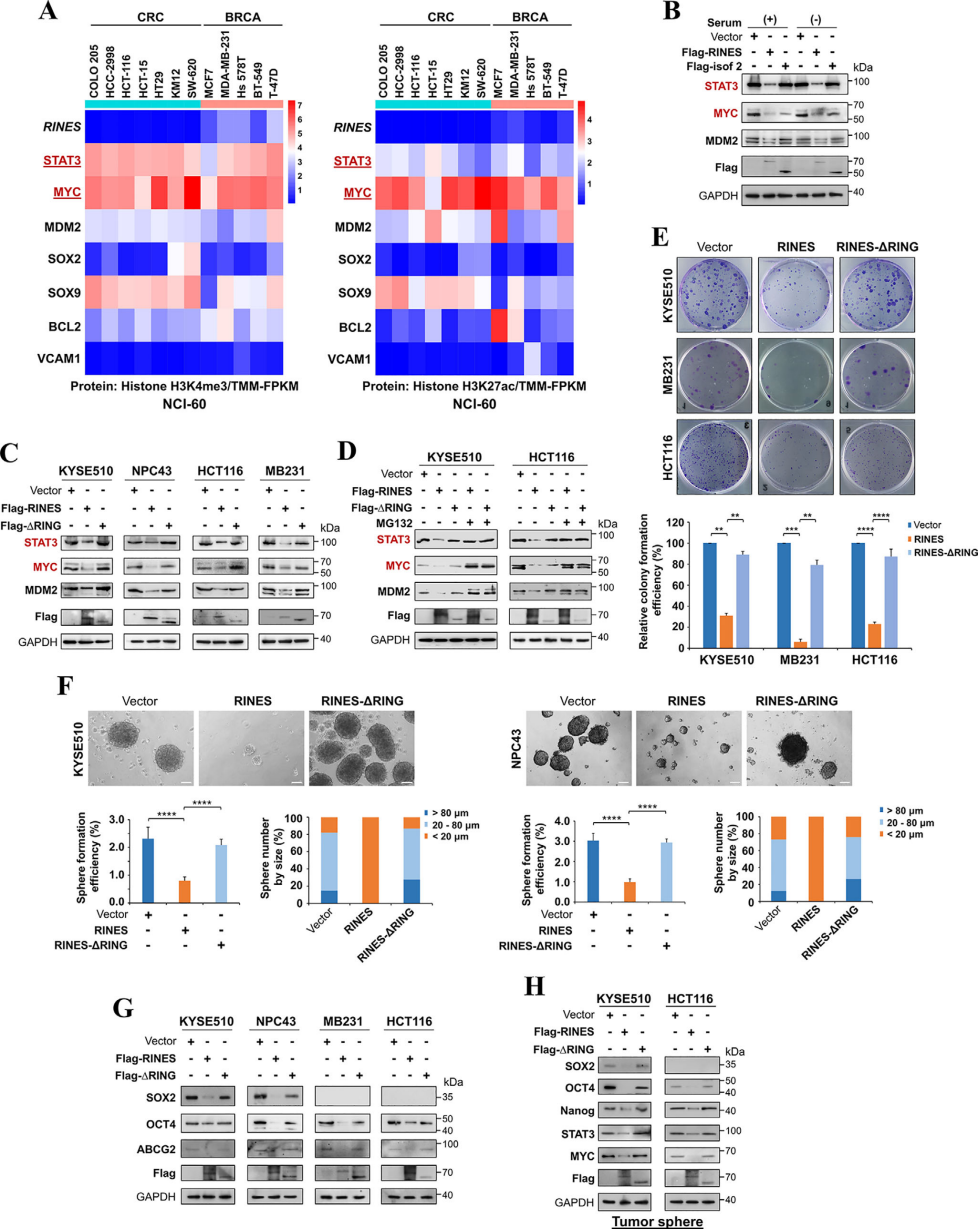
RINES suppresses tumor sphere formation by inhibiting stemness regulators via its RING domain. (A) Heatmap showing expression of RINES and selected stemness regulators. Analysis was performed on analysis of NCI60 cell lines from the CellMiner database using TMM‐FPKM (log_2_)‐normalized H3K4me3 and H3K27ac ChIP‐seq data. (B) Western blot analysis demonstrating reduced levels of stemness regulators (STAT3, MYC and MDM2) in tumor cell lines expressing RINES, but not isoform 2, under normal culture or 24 h serum starvation. MB231 cells expressing RINES or isoform 2 were cultured in serum‐free medium. (C) Western blot analysis of STAT3, MYC, and MDM2 expression in tumor cell lines expressing RINES or the RING‐domain mutant. (D) MG132 (proteasome inhibitor, 10 µM for 6 h) treatment reversed RINES‐mediated downregulation of endogenous STAT3 and MYC in KYSE510 and HCT116 cells expressing a control vector, RINES, or the RING‐domain mutant, as shown by Western blot. (E) Representative images of colony formation assays using monolayer‐cultured tumor cells expressing RINES or the RING‐domain mutant. Quantitative analysis of colony numbers is presented as mean ± SD from three independent experiments. Colonies containing more than 50 cells in the empty vector‐transfected cells set as 100 for normalization. *p*‐values were calculated using one‐way ANOVA with Tukey's post hoc tests. ^**^, *p* < 0.01; ^***^, *p* < 0.001; ^****^, *p* < 0.0001. (F) Representative images of tumor spheroids formed by KYSE510 and NPC43 cells expressing RINES or RING‐domain mutant after 7‐day incubation. Graphs show sphere‐forming efficiency and size distribution. Data are represented as mean ± SD of three independent experiments. Statistical analysis was performed using one‐way ANOVA with Tukey's post hoc test, and representative data were shown. ^****^, *p* < 0.0001. Scale bar, 50 µm. (G, H) Western blot analysis of stem cell marker expression in parent cells (G) and tumor spheres (H) expressing RINES or the RING‐domain mutant.

To elucidate the molecular mechanisms underlying RINES‐mediated tumor suppression, we analyzed CellMiner datasets and found that *RINES* expression levels are negatively correlated with those of key stemness regulators (STAT3, MYC, and MDM2) in colorectal and breast cancer cell lines from the NCI‐60 panel (Figure 4A). We also assessed the expression patterns of RINES alongside STAT3 and MYC in clinical tumor samples using the UALCAN/TCGA database [46]. Results showed that RINES expression was negatively correlated with STAT3 and MYC expression in multiple tumor types, including ESCA, HNSC, COAD, READ, and BRCA (Figure S6).

Consistent with these findings, ectopic RINES expression reduced the protein abundance of STAT3, MYC, and MDM2 in tumor cells under both normal culture and serum‐starved conditions. Notably, this repressive effect was not observed in cells overexpressing either isoform 2 (Figure 4B) or the RING domain mutant (Figure 4C). Moreover, treatment with the proteasome inhibitor MG132 restored the levels of these stemness markers that were downregulated by RINES (Figure 4D), indicating that RINES facilitates the proteasomal degradation of these proteins in a RING domain‐dependent manner.

We further examined the essential role of the RING domain in RINES‐mediated tumor suppression. Ectopic expression of full‐length RINES in KYSE510, MB231, and HCT116 cells markedly impaired colony formation, whereas this inhibitory activity was largely abrogated in cells expressing the RING‐domain mutant (Figure 4E). In addition, ESCC (KYSE510) and NPC (NPC43) cells with RINES overexpression exhibited a prominent reduction in both the number and size of tumor spheres, an effect that was not recapitulated by the RING‐domain mutant (Figure 4F). Accordingly, we observed markedly decreased protein levels of stemness markers (SOX2, OCT4, ABCG2, and NANOG) in parental cells and tumor spheres derived from RINES‐expressing KYSE510 and NPC43 cells, but not in cells expressing the RING‐domain mutant (Figure 4G,H). These results demonstrate that RINES suppresses cancer cell stemness properties by downregulating key stemness regulators via its functional RING domain.

### 3.6 RINES Directly Interacts With STAT3 and MYC to Facilitate Their Ubiquitin‐Mediated Degradation

**FIGURE 5:**
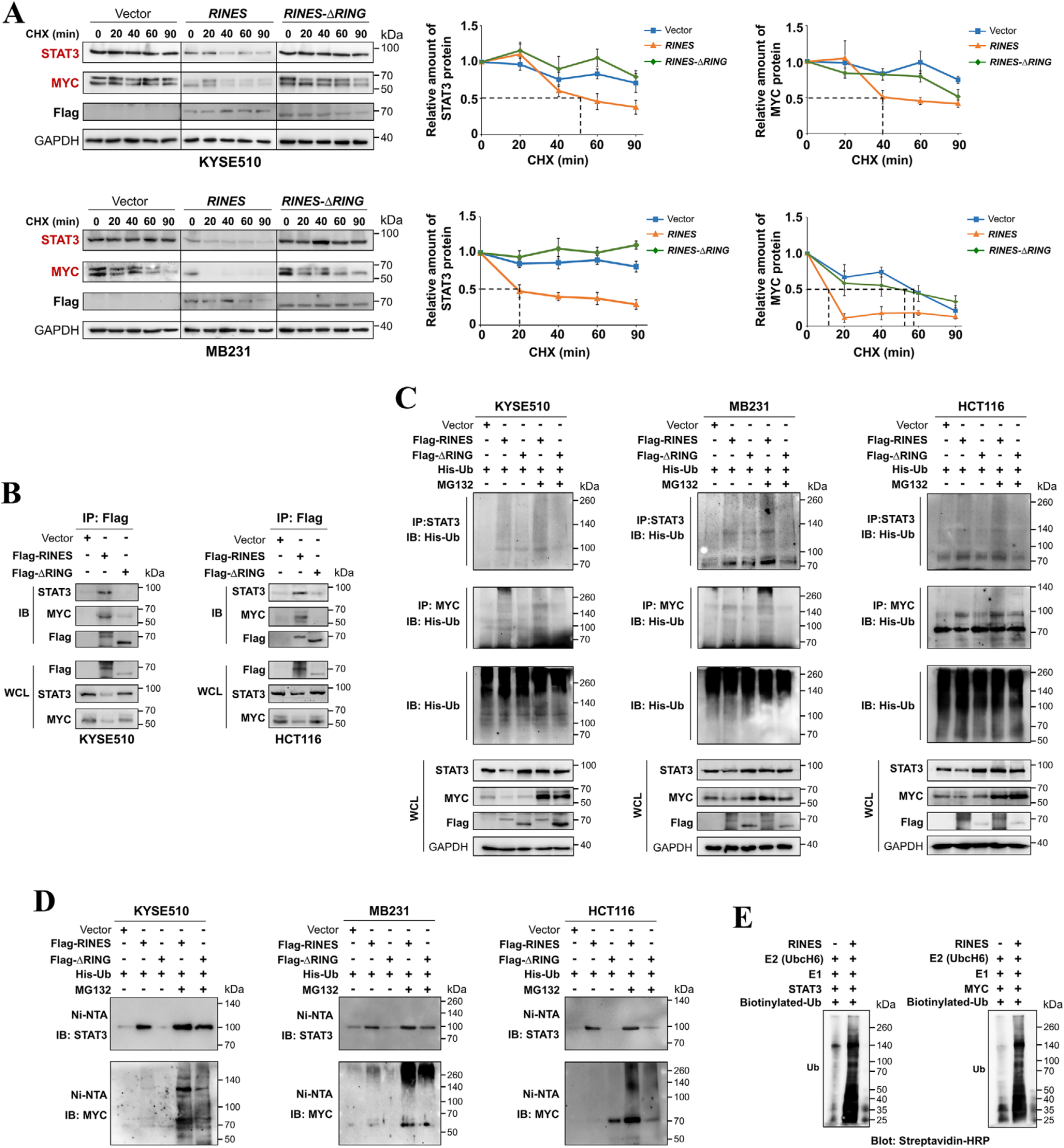
RINES mediates ubiquitination and degradation of STAT3 and MYC. (A) RINES, but not its RING‐domain mutant, shortens the half‐life of STAT3 and MYC proteins. KYSE510 and MB231 cells expressing RINES or the RING‐domain mutant were treated with 20 µg/mL cycloheximide (CHX) for the indicated durations, and protein levels were examined by Western blot using antibodies against STAT3 and MYC (left). Quantification of STAT3 and MYC levels normalized to GAPDH is shown (right, mean ± SD). Data are represented as the mean ± SD of three independent experiments. (B) RINES interacts with STAT3 and MYC. Co‐immunoprecipitation (Co‐IP) assays revealed that RINES, but not its RING‐domain mutant, binds endogenous STAT3 and MYC proteins in KYSE510 and HCT116 cells. Cell lysates were immunoprecipitated with anti‐Flag antibody, and bound proteins were detected by Western blot using antibodies against STAT3, MYC, and Flag. (C) RINES promotes ubiquitination and proteasomal degradation of endogenous STAT3 and MYC. Ubiquitination levels were analyzed by transfecting His‐tagged ubiquitin into tumor cells, followed by immunoblotting of His‐Ub in STAT3 or MYC immunoprecipitates. Cells were treated with or without 10 µM MG132 for 6 h. (D) Ectopic expression of RINES increases endogenous ubiquitination of STAT3 and MYC. Cells expressing His‐ubiquitin were treated with 10 µM MG132 for 6 h, followed by Ni‐NTA pull‐down. Ubiquitinated STAT3 and MYC were then detected by Western blot. (E) In vitro ubiquitination assays were carried out with recombinant E1, UbcH6c (E2 ubiquitin‐conjugating enzyme), and 100 nM GST‐RINES (E3 ubiquitin ligase), together with biotinylated ubiquitin (Ub), ATP, and purified STAT3 and MYC as substrates. Ubiquitinated STAT3 and MYC were detected by Western blot using a Streptavidin‐HRP antibody.

To determine whether STAT3 and MYC are downstream targets of RINES for ubiquitination and degradation, we inhibited protein synthesis using cycloheximide (CHX) and assessed STAT3 and MYC protein levels upon RINES expression. Forced expression of RINES in KYSE510 and MB231 cells, but not its RING‐domain mutant, significantly reduced the stability of STAT3 and MYC (Figure 5A). Next, we examined the interaction between RINES and STAT3/MYC via overexpression assays. Co‐immunoprecipitation results showed that endogenous STAT3 and MYC co‐precipitated with ectopically expressed RINES in KYSE510 and HCT116 cells, whereas no binding was detected with the RING‐domain mutant (Figure 5B). These results suggest that STAT3 and MYC are direct binding partners and ubiquitination targets of RINES.

We next investigated whether RINES destabilizes STAT3 and MYC through the ubiquitin‐proteasome system. Following MG132 treatment, we detected increased ubiquitination of STAT3 and MYC (Figure 5C). Ectopic expression of RINES, but not its RING mutant, substantially enhanced the ubiquitination of endogenous STAT3 and MYC in KYSE510, MB231, and HCT116 tumor cells (Figure 5C). In addition, Ni‐NTA affinity pull‐down assays revealed that RINES overexpression prominently elevated monoubiquitination of endogenous STAT3 and polyubiquitination of endogenous MYC (Figure 5D). We then performed in vitro ubiquitination assay with purified STAT3 and MYC proteins and found that the addition of 100 nM RINES strongly induced ubiquitination of STAT3 and MYC, while only minimal ubiquitination was observed in the absence of RINES (Figure 5E). These results demonstrate that RINES directly promotes the ubiquitination of STAT3 and MYC.

**FIGURE 6:**
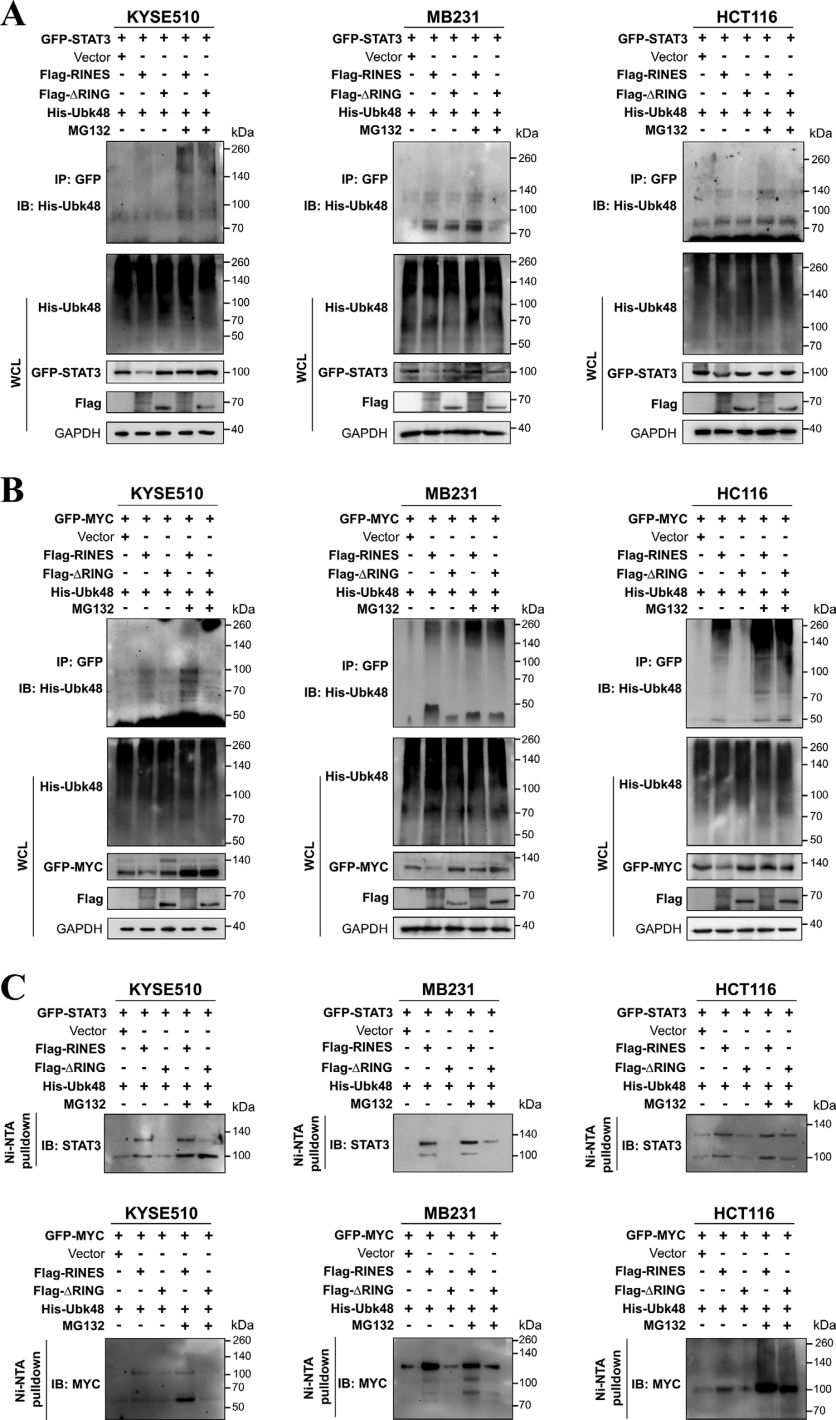
RINES acts as an E3 ubiquitin ligase for STAT3 and MYC. (A, B) Ubiquitination of STAT3 (A) and MYC (B) was assessed in tumor cells expressing RINES or its RING‐domain mutant. Cells were transfected with His‐tagged K48‐linked ubiquitin together with GFP‐STAT3 or GFP‐MYC, and treated with or without 10 µM MG132 for 6 h. GFP immunoprecipitates were analyzed via anti‐His immunoblotting. (C) RING domain is required for RINES‐mediated ubiquitination of STAT3 and MYC. Cells expressing RINES or the RING‐domain mutant were transfected with His‐tagged K48‐linked ubiquitin and GFP‐STAT3/GFP‐MYC plasmids, followed by treatment with 10 µM MG132 for 6 h as indicated. Ni‐NTA pull‐down assays combined with Western blot analysis confirmed RING domain‐dependent ubiquitination.

It is well established that the proteasome preferentially recognizes and degrades proteins conjugated with K48‐linked polyubiquitin chains [51]. To determine whether RINES facilitates K48‐linked ubiquitination of STAT3 and MYC, we employed a His‐tagged ubiquitin mutant carrying only a single lysine residue at position 48 (ubiquitin‐K48). Results demonstrated that RINES specifically catalyzed K48‐linked ubiquitination of STAT3 and MYC, with an effect comparable to that observed for wild‐type ubiquitin (Figure 6A,B). Ni‐NTA ubiquitination assays further validated that RINES enhances K48‐linked monoubiquitination of STAT3 and K48‐linked polyubiquitination of MYC (Figure 6C). Thus, these results suggest that RINES promotes the assembly of K48‐linked polyubiquitin chains on both STAT3 and MYC.

**FIGURE 7:**
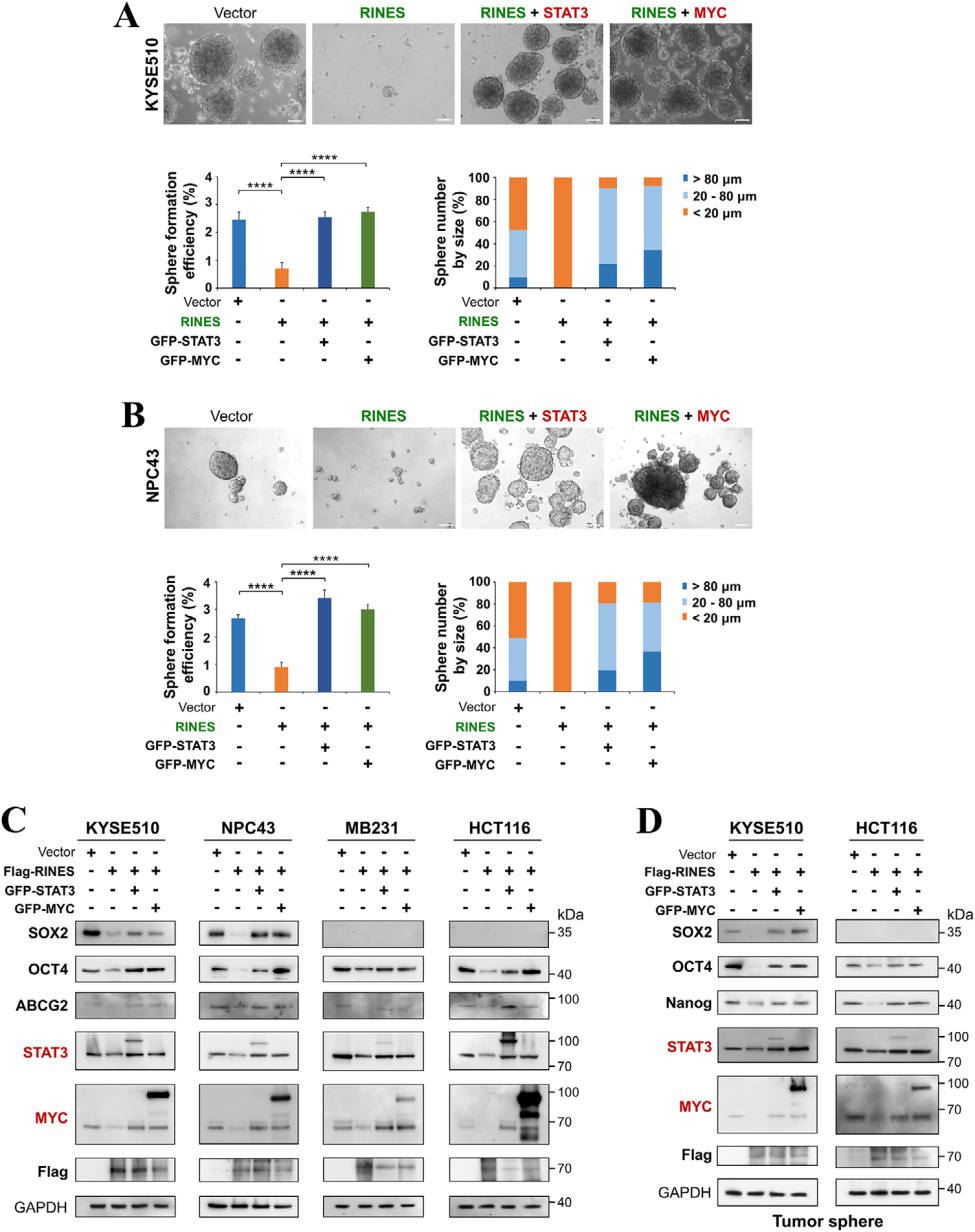
Increased STAT3 and MYC expression rescues RINES‐induced stemness suppression. (A, B) Tumor spheroid formation was assessed in RINES‐expressing KYSE510 (A) and NPC43 (B) cells transfected with GFP‐STAT3 or GFP‐MYC. Representative images of tumor spheres were captured after 7 days of culture. Graphs show the quantitative analysis of sphere‐forming efficiency and size distribution. Data are presented as mean ± SD of three independent experiments. Statistical significance was determined by one‐way ANOVA with Tukey's post hoc test. ^****^, *p* < 0.0001. Scale bar, 50 µm. (C, D) Western blot analysis of stemness regulators and stem cell markers in RINES‐expressing tumor cells (C) and their derived tumor spheres (D) following STAT3 or MYC restoration.

Functionally, restoration of STAT3 and MYC expression in KYSE510 and NPC43 cells fully rescued the inhibitory effects of RINES on the efficiency and size of tumor sphere formation (Figure 7A,B). This rescue effect was further supported by the upregulation of stemness markers (SOX2, OCT4, NANOG, or ABCG2) in both parental cells and tumor spheres (Figure 7C,D). Collectively, these results demonstrate that RINES suppresses tumor stemness by accelerating the ubiquitination and degradation of the stemness‐associated oncoproteins STAT3 and MYC.

### 3.7 RINES Knockdown Enhances Tumor Stemness by Increasing STAT3 and MYC Stability

**FIGURE 8:**
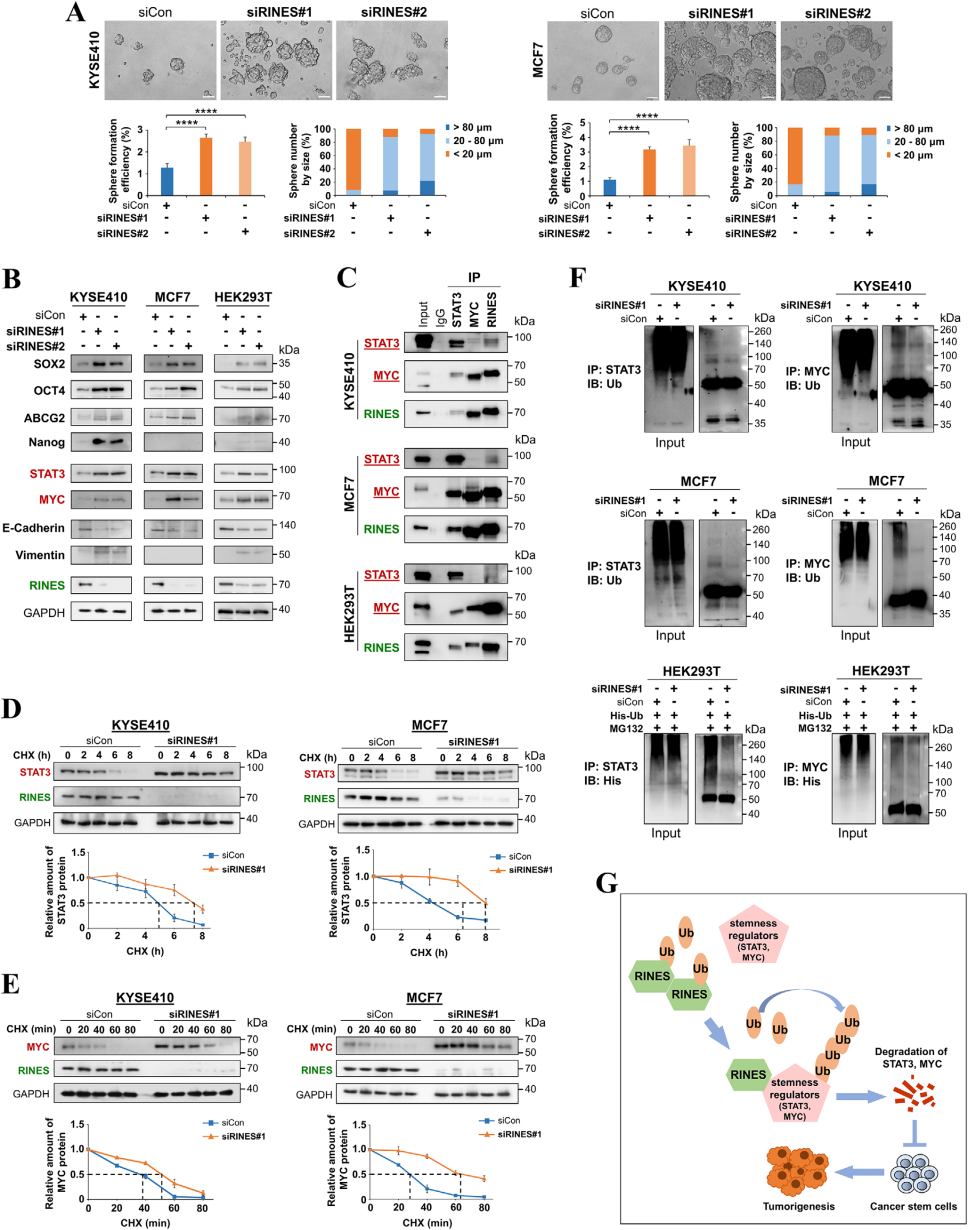
RINES depletion enhances stem‐like properties by upregulating STAT3 and MYC. (A) Tumor sphere formation in KYSE410 and MCF7 cells transfected with control siRNA or RINES siRNA. Representative images of tumor spheres were acquired after 7 days of culture. Graphs quantify sphere‐forming efficiency and size distribution. Data are represented as mean ± SD of three independent experiments by one‐way ANOVA with Tukey's post hoc test, and representative data are shown. ^****^, *p* < 0.0001. Scale bar, 50 µm. Con, Control. (B) Western blot analysis revealed elevated STAT3 and MYC protein levels upon RINES knockdown in KYSE410, MCF7, and HEK293T cells, using the indicated antibodies. (C) Interaction between RINES and endogenous STAT3 or MYC. Cell lysates from KYSE410, MCF7, and HEK293T cells with endogenous RINES expression were subjected to co‐IP with IgG or target‐specific antibodies, followed by Western blot detection. (D, E) RINES knockdown extends the half‐life of endogenous STAT3 (D) and MYC (E) proteins. CHX chase assays were performed with 10 µg/mL CHX in KYSE410 and MCF7 cells transfected with control or RINES siRNA. Relative protein levels of STAT3 and MYC (normalized to GAPDH) are presented as mean ± SD of three independent experiments. (F) RINES knockdown reduced the ubiquitination of endogenous STAT3 and MYC, as detected by IP‐Western analysis. (G) Schematic model illustrating the mechanism by which RINES regulates cancer stemness. RINES mediates RING domain‐dependent ubiquitination and degradation of the stemness regulators STAT3 and MYC during tumorigenesis.

To determine whether RINES regulates tumor stemness, we performed siRNA‐mediated RINES knockdown in RINES‐expressing ESCC (KYSE410), breast (MCF7), and immortalized HEK293T cells. Knockdown with two independent siRNAs significantly promoted tumor sphere formation (Figure 8A). This effect was accompanied by increased protein levels of STAT3 and MYC, upregulated expression of stemness markers (SOX2, OCT4, ABCG2, and Nanog), and altered epithelial‐mesenchymal transition (EMT) profiles, as indicated by reduced E‐cadherin and elevated vimentin levels (Figure 8B). Furthermore, RINES‐depleted MCF7 cells displayed significantly enhanced wound healing capacity and Matrigel migration/invasion ability relative to control cells (Figure S5F–H).

We also confirmed that endogenous RINES interacts with STAT3 and MYC, as validated by co‐immunoprecipitation assays in KYSE410, MCF7, and HEK293T cells (Figure 8C). We next examined the effect of RINES knockdown on the protein turnover of STAT3 and MYC. CHX chase assays revealed that the half‐lives of both proteins were prolonged in RINES‐depleted KYSE410 and MCF7 cells (Figure 8D,E). Consistently, the ubiquitination levels of STAT3 and MYC were significantly decreased following RINES knockdown (Figure 8F). These results suggest that RINES knockdown potentiates tumor stemness by suppressing ubiquitin‐dependent degradation of STAT3 and MYC in tumor cells.

## 4 Discussion

Multiple RNF family proteins are frequently disrupted by genetic and epigenetic alterations in human cancers, contributing to tumorigenesis and progression [7, 8, 18]. This study identifies *RINES/RNF180* as a methylated tumor suppressor in multiple cancers. Promoter CpG methylation silences *RINES*, correlating with poor survival and metastasis. RINES inhibits tumor growth, invasion, and stemness via RING‐dependent degradation of STAT3 and MYC, establishing it as a diagnostic/prognostic biomarker (Figure 8G).

Promoter CpG methylation is a well‐recognized mechanism underlying the inactivation of tumor suppressor genes in cancer. *RINES* promoter methylation occurs frequently across multiple tumor types, yet is rarely detected in normal tissues. No recurrent inactivating mutations (nonsense or frameshift) in *RINES* have been reported in human tumors, suggesting epigenetic silencing as the predominant mechanism. *RINES* methylation detected in nasal swabs of NPC patients suggests non‐invasive diagnostic potential. Similar findings have been documented in gastric cancer, where promoter methylation detected in non‐invasive samples correlates with poor patient prognosis [35]. Demethylating agents (e.g., azacitidine, decitabine) and HDAC inhibitors could restore RINES expression, yet conventional DNMT inhibitors have poor tumor specificity and side effects [24]. Optimized dosing, RINES‐mimetic PROTACs, and several repurposed drugs (GLP‐1‐based therapies, imidazo[2,1‐b]quinazoline derivative, and conformer‐selective thymidylate synthase inhibitors) are potential alternatives [52, 53, 54]. Future studies should evaluate these therapeutic strategies in preclinical models.

RINES/Rines was originally characterized as a novel RING finger protein interacting with the zinc finger transcription factor Zic2 [30]. Through its basic coiled‐coil domain, mouse Rines binds to Zic2 and promotes its ubiquitination and subsequent proteasomal degradation, a process implicated in congenital forebrain malformations [55]. However, Zic2 protein expression remained unchanged in the brains of *Rines*‐knockout mice, indicating that Rines/RINES may possess additional, as yet unidentified, biological functions.

Human RINES exhibits substantial homology to its mouse counterpart and functions as a tumor suppressor controlling cell proliferation and metastasis in gastric cancer [29, 30, 31]. Accumulating evidence also links this gene to other cancers, including lung [56], colon [57], breast [58], ovarian [59], and osteosarcoma [60]. Here, we demonstrate the tumor‐suppressive effects of RINES in ESCC, NPC, and breast cancer. We show that RINES suppresses cell proliferation and metastasis, promotes apoptosis, and inhibits cancer stemness throughout tumor development. Collectively, these data reinforce RINES as a widespread tumor suppressor.

In this study, we elucidated the molecular mechanisms by which RINES exerts its tumor‐suppressive activity. Aberrant upregulation and hyperactivation of the oncogenic STAT3 and MYC pathways represent pervasive hallmarks across human cancers [61]. STAT3 signaling contributes profoundly to every stage of tumorigenesis, ranging from tumor initiation to metastasis, and is a central regulator of cancer cell stemness [62]. Multiple studies have shown that dysregulated MYC promotes the acquisition and retention of stem cell features and maintains aggressive cancer stem cell populations [61, 63, 64]. Through gain‐ and loss‐of‐function experiments, we found that RINES impairs tumor sphere formation and modulates the key stemness regulators STAT3 and MYC, as well as downstream stemness markers.

Cancer stemness is well recognized as a critical driver of tumor initiation, EMT progression, and metastatic dissemination [65]. Consistently, RINES potently suppresses tumor cell invasion and metastasis. Mechanistically, this anti‐metastatic phenotype is linked to elevated expression of the epithelial marker E‐cadherin and reduced levels of the mesenchymal marker vimentin. Notably, removal of the RING domain fully abolishes RINES‐mediated inhibition of cell proliferation, metastasis, and tumor sphere formation, confirming that this domain is indispensable for the tumor‐suppressive functions of RINES. Taken together, our data establish RINES as a crucial modulator of the stemness–EMT–metastasis axis in cancer progression.

Deregulation of RNFs leads to abnormal ubiquitination of cancer proteins, perturbing the stability of tumor suppressors or the degradation of oncoproteins, thereby promoting cancer progression. E3 ubiquitin ligases play a vital role in the UPS by regulating the specificity, timing, and subsequent degradation of ubiquitination [66]. The UPS also serves as the primary machinery for most ER‐associated degradation [55]. RINES localizes to the ER in tumor cells, supporting its ER‐associated role [28]. Unlike the other types of E3 ubiquitin ligases, RING‐type E3 ubiquitin ligases function as scaffolds or interaction mediators, thereby enabling E2 enzymes to transfer ubiquitin to specific substrates [67, 68]. We demonstrate that RINES controls the stability of STAT3 and MYC via post‐translational regulation. By directly binding to these two oncoproteins through its RING domain, RINES facilitates their K48‐linked ubiquitination and subsequent degradation, further suppressing tumorigenesis and disease progression. Notably, the intrinsic E3 ligase activity of RINES strengthens the formation of RINES–STAT3 and RINES–MYC complexes, leading to elevated ubiquitination levels of both substrates.

Previous studies have identified several additional substrates of human RINES, such as DNMT1 [30], DNMT3A [31], and SOX2 [59]. Although RINES mediates their ubiquitination and eventual degradation, whether this regulation occurs through direct binding remains uncharacterized. Together with our current results, these data support that RINES is a functional E3 ligase capable of mediating ubiquitination‐dependent degradation of diverse oncoproteins in cancer. Our work also advances two previous investigations into RINES/RNF180‐mediated regulation of STAT3 and MYC. One report showed that RNF180 inhibits STAT3 indirectly by degrading RhoC, leading to decreased STAT3 phosphorylation with no change in total STAT3 protein abundance [69]. The other demonstrated RNF180‐induced c‐Myc degradation in non‐small cell lung cancer [70]. In contrast to these findings, we confirm STAT3 and MYC as direct substrates of RINES across various cancer types and prove that RINES catalyzes their K48‐linked ubiquitination. These results establish that RINES directly targets both STAT3 and MYC for degradation, acting through a more direct and widespread mode of action than was earlier proposed.

MYC and STAT3 are modulated by a complex regulatory network consisting of ubiquitin, SUMO, NEDD8, ISG15, and their associated E3 ligases and deubiquitinases [17, 71]. These UBLs (ubiquitin‐like) and regulatory enzymes function through several mechanisms: competing for overlapping lysine residues on target proteins to produce antagonistic effects, forming dynamic E3–DUB (deubiquitinating enzyme) cycles that govern protein stability, and establishing sequential modification cascades to alter substrate function. The potential involvement of additional UBLs in RINES‐induced ubiquitination of MYC and STAT3 remains to be elucidated in follow‐up research. Notably, FBXW7 mediates phosphorylation‐dependent c‐Myc degradation [72, 73], whereas RINES targets both STAT3 and MYC independently of protein phosphorylation, relying on its RING domain for this activity. Unlike RNF43 (a key Wnt signaling regulator) and FBXW7 (a critical cell cycle regulator), RINES suppresses cancer stemness owing to its dual‐substrate specificity. This property distinguishes RINES as a promising therapeutic candidate for MYC/STAT3‐dependent cancers.

Several limitations of this study should be addressed. First, the precise interaction domains and binding motifs governing assembly of the RINES–STAT3 and RINES–MYC complexes have not been fully defined. Correlative IHC/ISH studies between RINES and STAT3/MYC in patient tissues or functional assessment in patient‐derived models are required to confirm translational relevance. In addition, it is yet to be determined whether RINES regulates other key stemness factors (SOX2, OCT4, and NANOG) via direct or indirect mechanisms. Second, future investigations are needed to clarify whether RINES exerts broader ubiquitination activity or mediates chain‐specific modifications (e.g., K63‐linked ubiquitination) on its substrates. Third, the potential functional cooperation between RINES and other ubiquitin machinery components, such as SKP1 and Cullins, remains uncharacterized. Fourth, combining in vivo limiting dilution assays with stemness functional markers (e.g., ALDH activity) would provide a more thorough assessment of RINES‐mediated inhibition of cancer stem cell properties.

## 5 Conclusion

In conclusion, we have identified *RINES* as a bona fide tumor suppressor frequently silenced by promoter methylation in multiple cancers. Mechanistically, RINES suppresses tumor cell proliferation, metastasis, and stemness by directly targeting STAT3 and MYC for RING domain‐dependent ubiquitination and degradation. Furthermore, tumor‐specific *RINES* methylation suggests its potential utility as a diagnostic biomarker for multiple common cancers beyond gastric cancer. A deeper understanding of the tumor suppressor functions and underlying mechanisms of *RINES* may facilitate the development of novel therapeutic strategies for human cancers.

## Funding

This study was supported by RGC‐GRF (#14115019; #14111924; #14102923), NSFC/RGC Joint Research Scheme (#N_CUHK419/24), CUHK group grant, and Johns Hopkins Singapore.

## Conflicts of Interest

The authors declare no conflicts of interest.

## Supporting information




**Supporting File 1**: advs76515‐sup‐0001‐SuppMat.docx.

**Supporting File 2**: advs76515‐sup‐0002‐DataFile.pdf.

## Data Availability

The data that support the findings of this study are available from the corresponding author upon reasonable request.
