# Supplementary material for: Loss of E3 Ubiquitin Ligase RINES via CpG Methylation Relieves Suppression of STAT3 and MYC, Facilitating Multiple Tumorigeneses
Source: Adv Sci (Weinh). 2026 Jul 14:e23684. Online ahead of print. doi: 10.1002/advs.202523684 (PMC13366374; doi:10.1002/advs.202523684)
Supplement: Supplementary file 1 — Supporting File 1: advs76515‐sup‐0001‐SuppMat.docx. [file ADVS-9999-e23684-s001.docx]

Supporting Information

Loss of E3 ubiquitin ligase RINES via CpG methylation relieves suppression of STAT3 and MYC, facilitating multiple tumorigeneses

*Lili Li*, Ka Man Ng, Xingsheng Shu, Xiaoxue Chai, Kai Yau Wong, Gopesh Srivastava, Anthony TC Chan, Wai Yee Chan, Qian Tao**

**Materials and Methods**

**Cell lines**

Esophageal, head and neck (nasopharyngeal), colorectal, gastric, breast, lung, kidney, cervix and liver cancer cell lines, including KYSE510 (RRID: CVCL_1354), KYSE410 (RRID: CVCL_1352), KYSE140 (RRID: CVCL_1347), KYSE150 (RRID: CVCL_1348), HKESC1 (RRID: CVCL_D568), HKESC2 (RRID: CVCL_D571), HKESC3 (RRID: CVCL_D572), C666-1 (RRID: CVCL_7949), HK1 (RRID: CVCL_7084), NPC43 (RRID: CVCL_UH64), FaDu (RRID: CVCL_1218), HCT116 (RRID: CVCL_0291), HCT15 (RRID: CVCL_0292), LoVo (RRID: CVCL_0399), HT-29 (RRID: CVCL_0320), Kato-III (RRID: CVCL_0371), YCCEL1 (RRID: CVCL_9647), YCC1 (RRID: CVCL_9646), MB231 (RRID: CVCL_0062), MCF7 (RRID: CVCL_0031), BT549 (RRID: CVCL_1092), H1299 (RRID: CVCL_0060), A549 (RRID: CVCL_0023), CaSki (RRID: CVCL_1100), HeLa (RRID: CVCL_0030), A498 (RRID: CVCL_1056), HH244 (RRID: CVCL_X532), HepG2 (RRID: CVCL_0027), Hep3B (RRID: CVCL_0027), and HuH-6 (RRID: CVCL_4381) were obtained from ATCC (Rockville, MD), DSMZ (Braunschweig, Germany) or our collaborators [1, 2]. Immortalized human normal epithelial cell lines, including NE1 (RRID: CVCL_E306), Het-1A (RRID: CVCL_3702), NP69 (RRID: CVCL_F755), CCD841-CoN (RRID: CVCL_2871), HMEC (RRID: CVCL_0307) and HEK293 (RRID: CVCL_0045) were maintained in KSFM or DMEM [1, 2].

**CpG methylomes**

CpG methylome analysis was performed using methylated DNA immunoprecipitation (MeDIP) coupled with promoter microarray hybridization (MeDIP-chip) [3]. Briefly, genomic DNA from tumor cell lines and primary tumor tissues was immunoprecipitated with a 5-methylcytidine monoclonal antibody (33D3, Diagenode, Seraing, Belgium), then purified, labeled and hybridized to NimbleGen™ HG18 Meth (385K CGI plus) promoter arrays (Array Star, Inc., MD, USA). Methylome data were analyzed using SignalMap by NimbleGen Systems, Inc. as previously described [1, 3].

**Semi-quantitative RT-PCR and Quantitative RT-PCR (qRT-PCR)**

Total RNA was isolated from cell lines and tumor tissues using TRIzol reagent (MRC, Cincinnati, OH, USA). For semi-quantitative RT-PCR, reactions were performed for 32 cycles using GoTaq® G2 DNA Polymerase (Promega, Madison, WI, USA) [1, 2, 4], with *GAPDH* as an internal control, as previously described. Quantitative RT-PCR was conducted using SYBR Green master mix on a StepOne™ Real-Time PCR System (Applied Biosystems, Foster City, CA, USA). Gene expression changes were quantified using the 2−ΔΔCt method normalized to GAPDH. Primer sequences are provided in Supplementary Table S2.

**Deletion analysis of *RINES* by multiplex PCR**

Homozygous deletion of *RINES* was examined using multiplex genomic DNA-PCR as previously described [5, 6]. Primer sequences are shown in Supplementary Table S2.

**Bisulfite treatment and promoter methylation analyses**

Bisulfite conversion of genomic DNA was performed followed by methylation-specific PCR (MSP) and bisulfite genomic sequencing (BGS) as described previously [4, 6, 7]. Both MSP and BGS were conducted for 40 cycles using AmpliTaq Gold DNA Polymerase with hot-start protocol. MSP primers were validated to exclude unbisulfited DNA amplification, confirming the specificity. For BGS, PCR products were cloned into pCR4-TOPO vector (Invitrogen, Carlsbad, CA). Six to ten colonies were randomly chosen and sequenced. Primers used in this study are listed in Supplementary Table S2.

**Colony formation, proliferation, and Transwell assays**

For monolayer colony formation assay, cells were plated in 12-well plates and transfected with RINES, isoform 2, RING-domain mutant, or empty vector control. At 24 h post-transfection, cells were replated into 6-well plates and selected with G418 (0.4 mg/mL) for 10-14 days. Colonies containing > 50 cells were fixed, stained with crystal violet and counted. For soft-agar colony formation assay, cells were suspended in growth medium containing 0.35% agar in 24-well plates. Surviving colonies were photographed and counted after approximately 2 to 3 weeks.

For cell proliferation assay, cells seeded in 96-well plates were assessed at designated time points using CCK-8 assay (MedChemExpress, Monmouth Junction, NJ). Following incubation with 10 μL CCK-8 reagent for 2 hours, absorbance was measured at 450 nm using a SpectraMax® iD3 microplate reader (Molecular Devices, San Jose, CA, USA).

For migration assay, cells suspended in serum-free medium were added to the upper chamber of 8 μm-pore Transwell insert (#3422, Corning, NY, USA). The lower chamber was filled with complete medium supplemented with 10% FBS as a chemoattractant. After 16-18 hours, migrated cells were fixed, stained with 0.1% crystal violet, and quantified by counting five randomly selected microscopic fields per insert under phase-contrast microscopy. Invasion assays were performed similarly using Matrigel-precoated inserts according to the manufacturer's protocol (#354483, Corning, NY, USA).

**Apoptosis analyses**

TUNEL staining was conducted utilizing the In Situ Cell Death Detection Kit (#12156792910, Roche Diagnostics, Indianapolis, IN, USA), followed by nuclear staining with DAPI and subsequent imaging as previously described [8]. HONE1 and HCT116 tumor cells were cotransfected with the EGFP-caspase-3-sensor reporter plasmid and either RINES, Isof2, or an empty vector. After 48 hours, cells with activated nucleus-localized caspase-3 were counted.

**Immunohistochemistry (IHC) staining**

Tissue microarrays (TMAs) were subjected to IHC staining on an Autostainer Link 48 (DAKO A/S, Glostup, Denmark) using the Dako EnVision™ FLEX+ kit (#K8002). The primary antibody against RINES/RNF180 (#HPA006897, Sigma-Aldrich, St Louis, MO, USA) was applied at a dilution of 1:500. High-resolution IHC images were obtained by Aperio Digital Pathology Slide Scanners (Aperio Scanscope XT, Leica Biosystems). Three fields of each sample were randomly chosen to calculate the percentage of positive cells. Staining results were scored by staining intensity and staining positive rate (0-300%).

**Immunoprecipitation**

Cells were lysed on ice for 30 min using RIPA buffer (50 mmol/L Tris-HCl, pH 8.0; 150 mmol/L NaCl; 0.5% NP-40) supplemented with protease inhibitor cocktail. For immunoblotting analysis, whole-cell lysates or immunoprecipitated lysates were separated via SDS-PAGE and electrophoretically transferred onto nitrocellulose membranes. Membranes were blocked, then incubated with primary antibodies, followed by corresponding HRP-conjugated secondary antibodies. For co-immunoprecipitation (co-IP) experiments, cell lysates were incubated with the indicated antibodies together with Protein‑G Sepharose 4 Fast Flow resin (Cytiva, Freiburg, Germany) according to optimized immunoprecipitation conditions. Immunoreactive bands were detected using Amersham ECL Prime Western Blotting Detection Reagent (#RPN2232, Cytiva, Freiburg, Germany) and visualized on the ChemiDoc Image System (Bio-Rad, Hercules, CA, USA) with ImageLab software.

**Immunofluorescence**

For endoplasmic reticulum (ER) labeling, cells were transfected with an ER-specific fluorescent plasmid (pDsRed-ER) for 48 h prior to fluorescent analysis. Subsequently, cells were incubated with anti-FLAG M2 monoclonal antibody (#F3165, Sigma, St. Louis, MO, USA) overnight at 4°C after permeabilization with 4% paraformaldehyde. Subsequently, cells were treated with Alexa Fluor® 488 (Invitrogen, Carlsbad, CA, USA) for 30 min at 37°C, followed by PBS washing. Cell nuclei were stained with 4',6-diamidino-2-phenylindole (DAPI) (Calbiochem, San Diego, CA, USA). Finally, cells were mounted onto glass slides and imaged using an Olympus BX51 fluorescence microscope (Olympus Corporation, Tokyo, Japan).


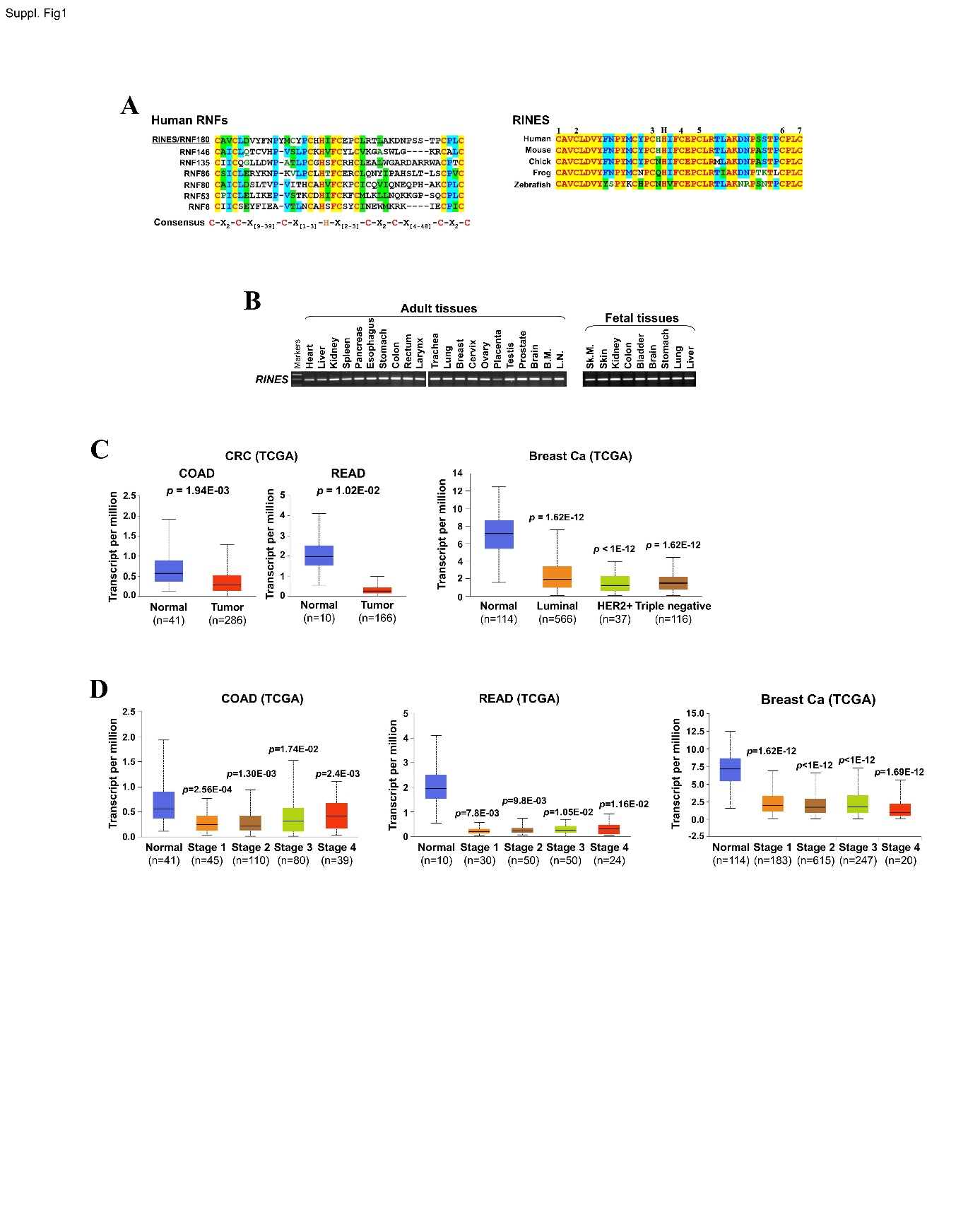


**Suppl. Figure 1.** Correlation between *RINES* downregulation and clinical features in colorectal and breast cancers. (A) Amino acid sequence alignment of the RING domain across seven human RNF proteins (left) and RINES homologs from different species (right). Sequence alignment was performed using ClustalX2. (B) *RINES* mRNA expression in a panel of human adult and fetal tissues was examined via semi-quantitative RT-PCR. *GAPDH* served as an internal control for RNA integrity (not shown). B.M, bone marrow; L.N., lymph nodes. (C, D) UALCAN analysis of TCGA datasets showing correlations of *RINES* mRNA levels with histological subtypes (C) and tumor stages (D) in colorectal and breast cancers. CRC, colorectal cancer; COAD, colon adenocarcinoma; READ, rectum adenocarcinoma; Ca, carcinoma.

**
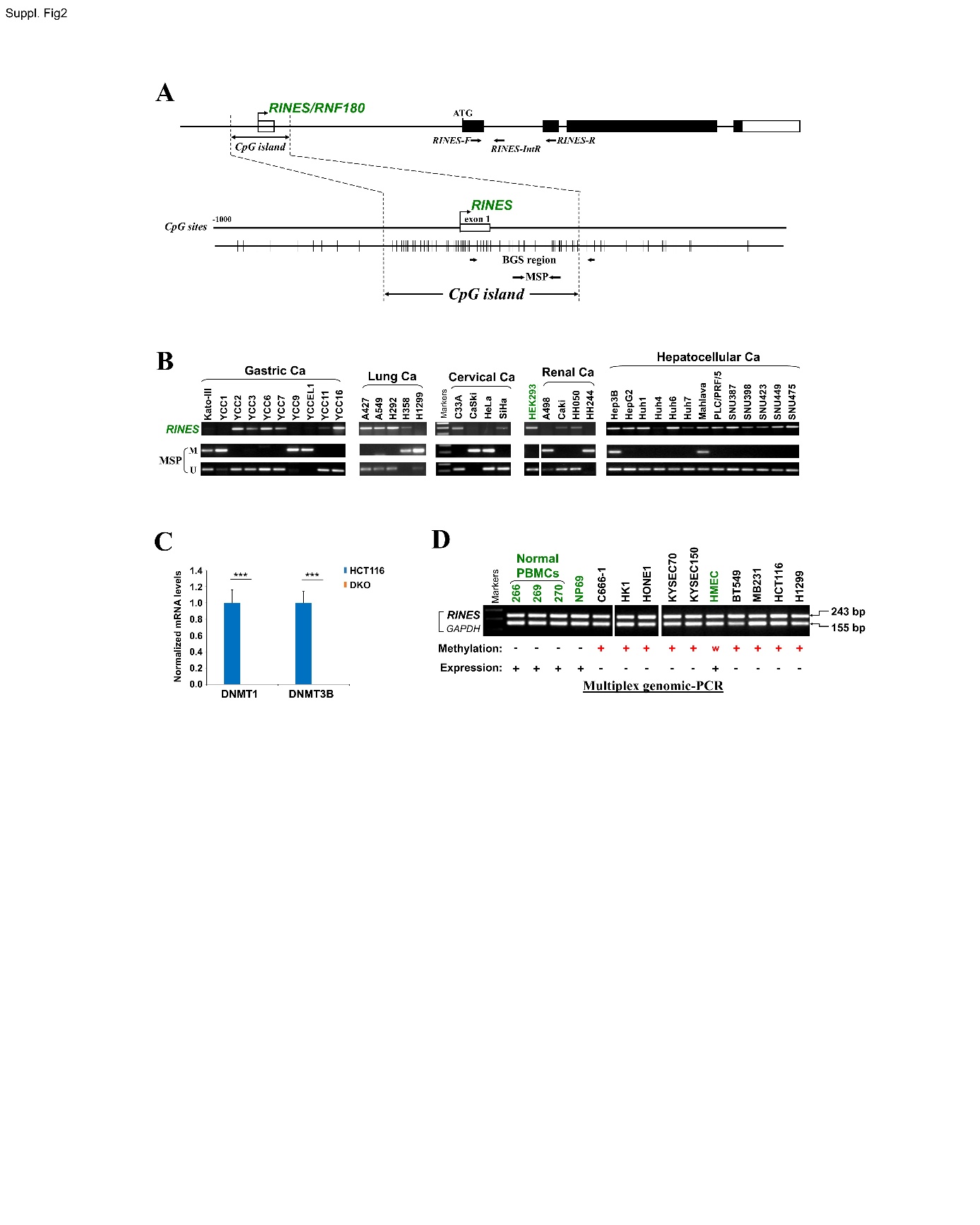
**

**Suppl. Figure 2.** (A) Schematic structure of the *RINES* CpG island (CGI). Locations of the promoter, exons and CpG sites (short vertical lines) are shown. The transcription start site is indicated by a curved arrow. Regions subjected to MSP and BGS analysis are highlighted. (B) *RINES* expression and methylation in multiple tumor cell lines from gastric, lung, liver, cervical, renal, and hepatocellular origins. Semi-quantitative RT-PCR was used to evaluate *RINES* expression at the mRNA level, and methylation-specific PCR (MSP) was used to examine DNA methylation status. *GAPDH* served as an internal control for RNA integrity (data not shown). M, methylated; U, unmethylated; Ca, cancer. (C) DNMT1 and DNMT3B expression levels in HCT116 and DKO cell lines were measured by quantitative real-time PCR. Data are represented as mean ± SD of three independent experiments, and statistical significance was determined via Student's t-test. ***, p < 0.001. (D) Representative *RINES* deletion analysis in tumor and normal cell lines via multiplex differential genomic DNA-PCR, with GAPDH as an internal control. w, weak.

**
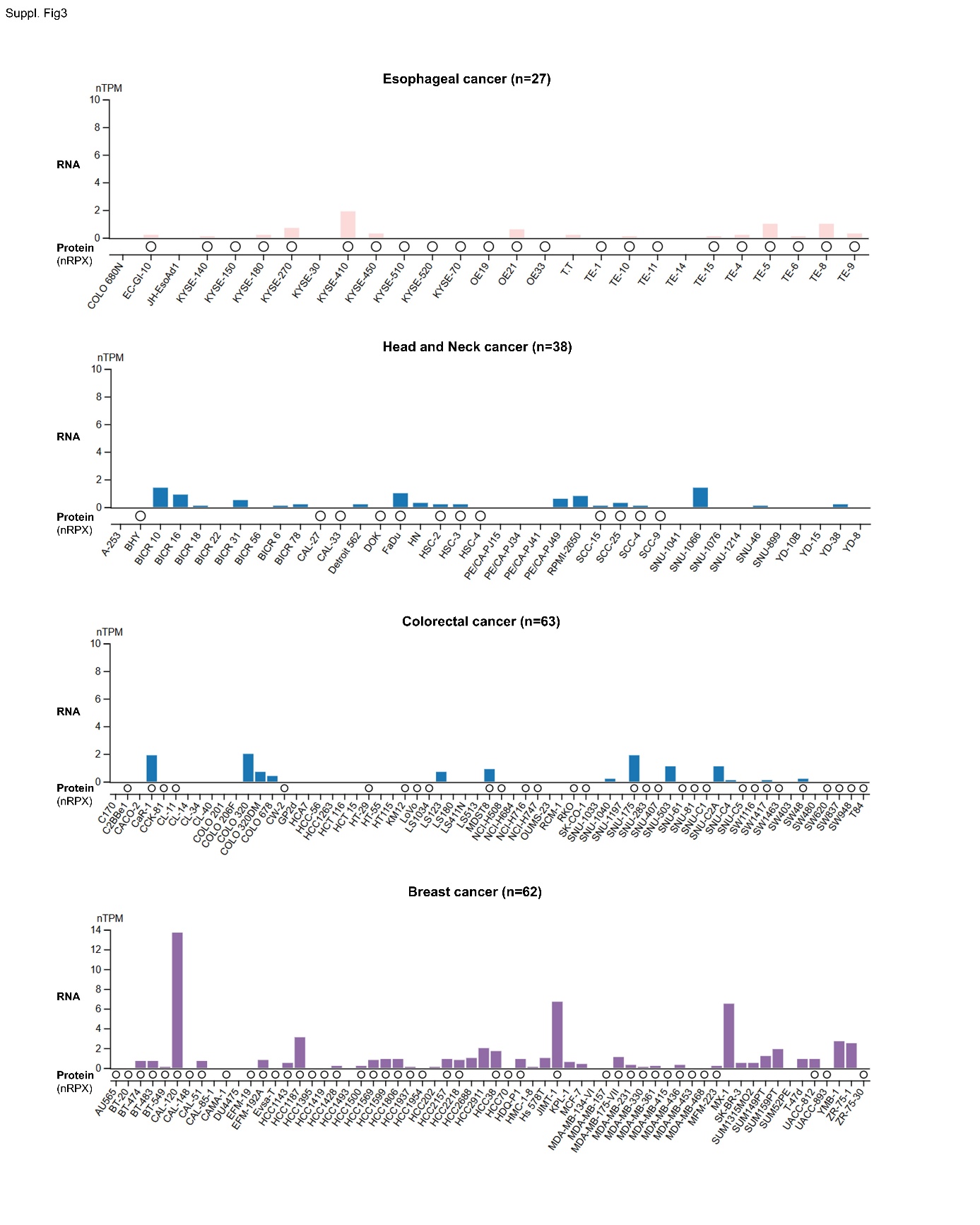
**

**Suppl. Figure 3.** RINES mRNA and protein expression in tumor cell lines from esophageal, head and neck, colon, and breast cancers (data retrieved from the Human Protein Atlas). mRNA abundance is presented as normalized transcripts per million (nTPM). Protein expression derived from mass spectrometry (MS)-based proteomics is represented by circles: circle size corresponds to normalized relative protein expression (nRPX); white circles indicate no detectable protein via MS; no circle indicates unavailable MS data.

**
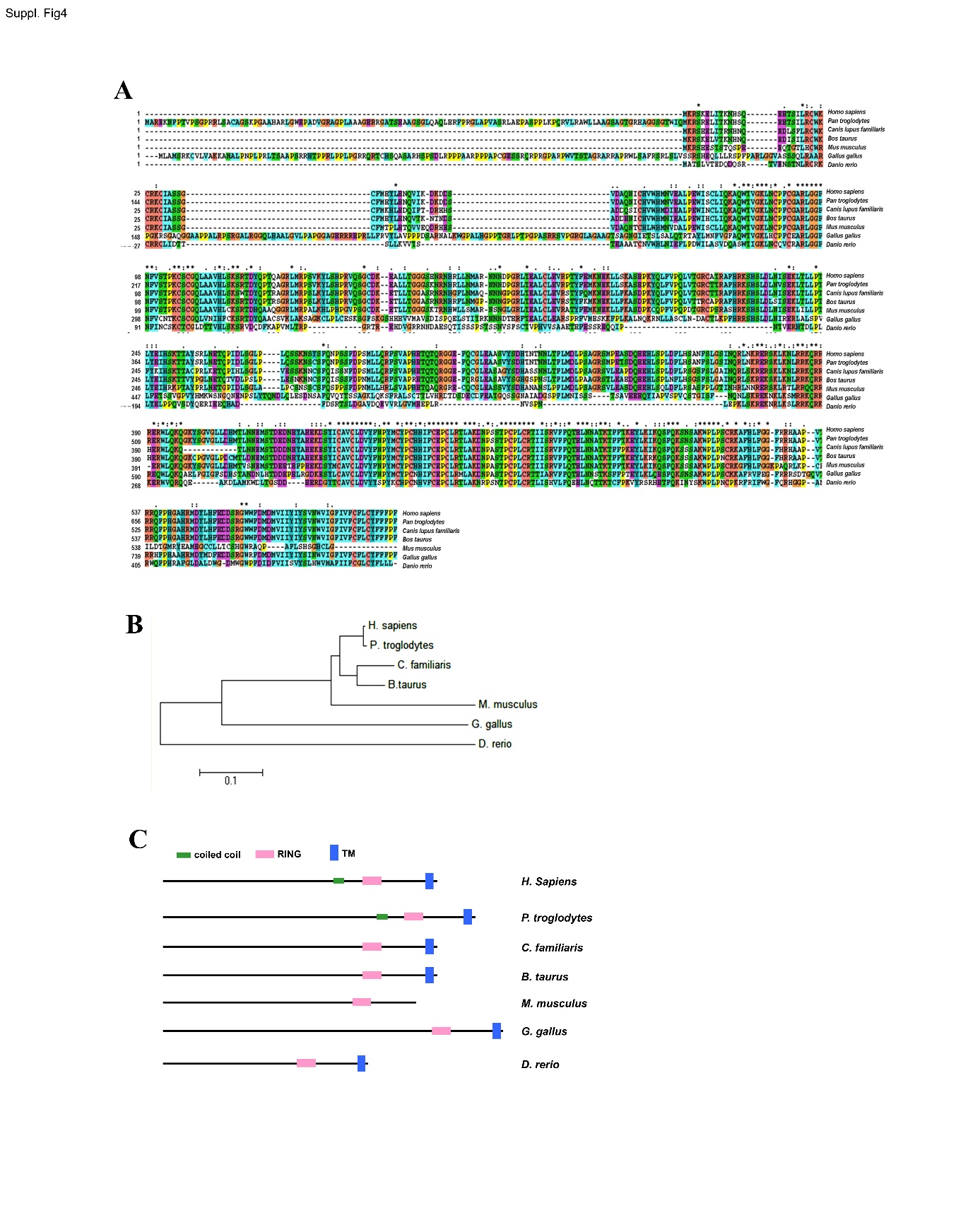
**

**Suppl. Figure 4.** RINES is an evolutionarily conserved protein. (A) Multiple-sequence alignment of RINES proteins across distinct species. An asterisk marks a fully conserved amino acid residue. (B) Phylogenetic tree constructed from cross-species RINES protein sequence alignment. (C) Interspecies comparison of predicted RINES protein domains. TM, transmembrane region. All analyses were performed based on the human reference genome assembly GRCh37/hg19.

**
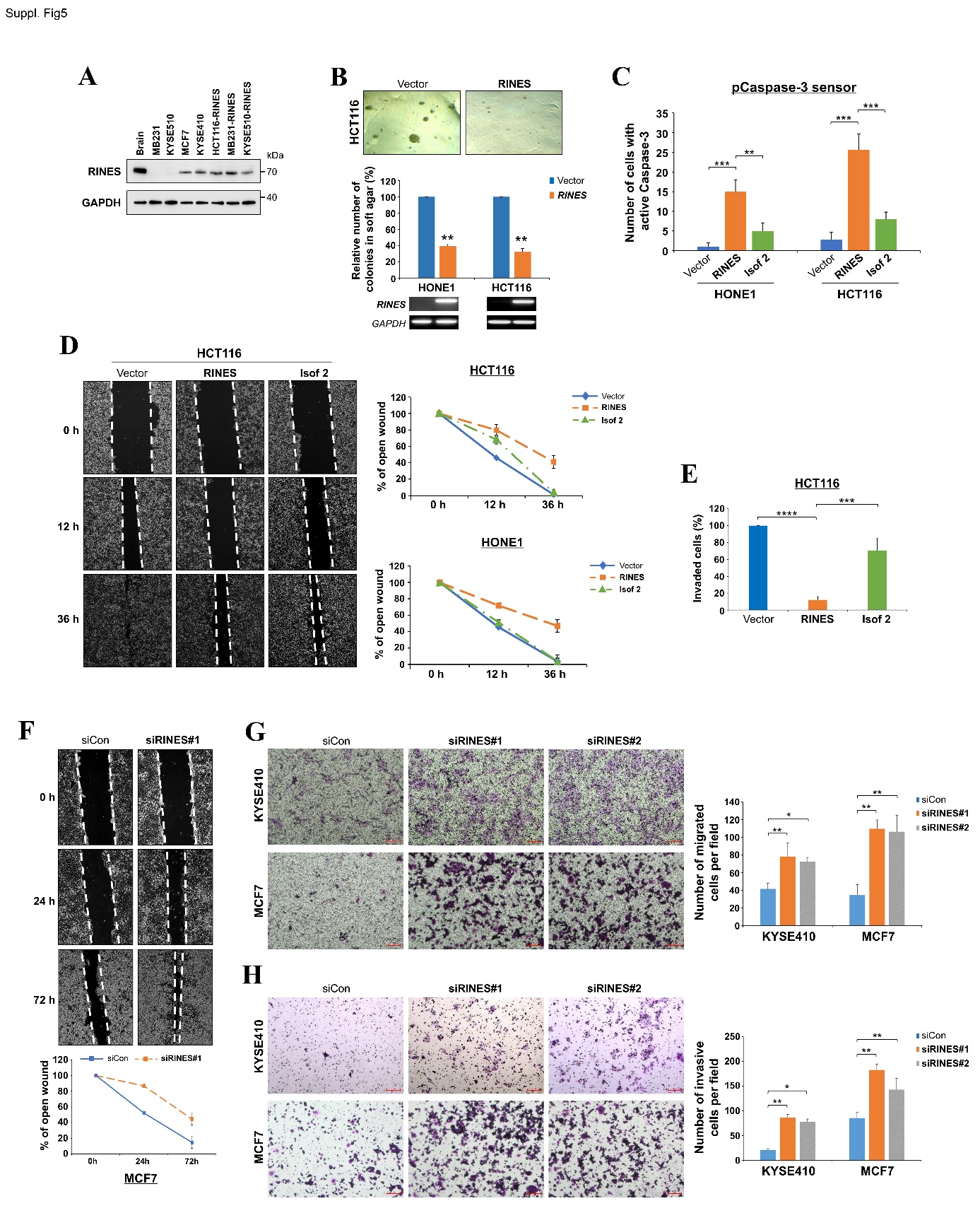
**

**Suppl. Figure 5.** RINES exerts the tumor suppressive function in tumor cells. (A) RINES protein expression in cell line and brain tissue samples. (B-D) Functional characterization of tumor-suppressive effects upon ectopic RINES overexpression. (B) Ectopic RINES suppresses anchorage-independent colony formation in HCT116 and HONE1 cells grown in soft agar. Quantitative colony counts are shown as mean ± SD from three independent biological replicates; statistical comparison was performed via Student’s t-test, with corresponding representative colony images provided. Colony counts (>50 cells per colony) of empty-vector control cells were normalized to 100. **, p < 0.01. Successful *RINES* overexpression in transfected HONE1 and HCT116 cells was validated by semi-quantitative RT-PCR, whereas no *RINES* transcript was detected in empty vector controls. (C) Re-expression of RINES, but not its isoform 2, induced caspase-3 activation. HONE1 and HCT116 tumor cells expressing RINES or isoform 2 were co-transfected with an EGFP-caspase-3 sensor reporter plasmid. Quantification of caspase-3 positive cells is presented as mean ± SD from three replicates by one-way ANOVA with Tukey's post hoc test. (D) Wound-healing migration assay for HONE1 and HCT116 cells overexpressing RINES or isoform 2. Residual wound width was quantified relative to the initial wound width at 0 h. Data are shown as mean ± SD of three independent experiments. (E) Transwell invasion assay of RINES- or isoform 2-expressing HCT116 tumor cells. Invaded cells adhering to the lower filter surface were fixed, stained and counted. Quantitative data are expressed as mean ± SD across three replicates, analyzed by one-way ANOVA with Tukey's post hoc test. (F-H) RINES knockdown enhances migratory and invasive capacities of tumor cells. (F) Wound-healing assay in RINES-depleted MCF7 cells. Remaining wound width was normalized to the initial width at 0 h. Data represent mean ± SD from three replicates. (G, H) Transwell migration (G) and invasion (H) assays in RINES-depleted KYSE410 and MCF7 cells. Cells penetrating the lower membrane were stained and counted. Quantitative results are displayed as mean ± SD from three independent experiments and analyzed via one-way ANOVA with Tukey's post hoc test.


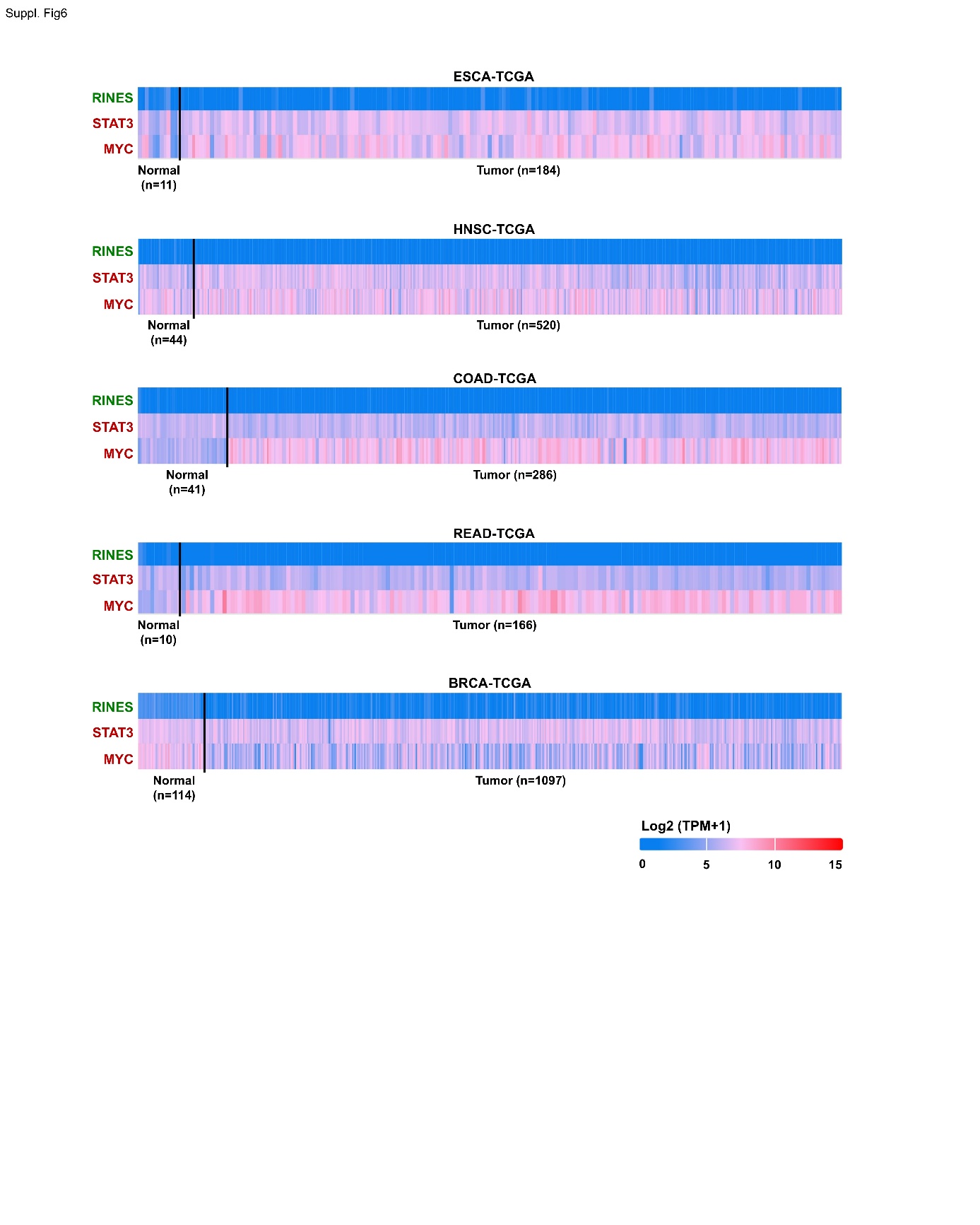


**Supple. Figure 6.** Heatmap illustrating RINES, STAT3, and MYC expression across esophageal, head and neck, colorectal, and breast cancer cohorts retrieved from the TCGA database, with data processed using the UALCAN portal. ESCA, esophageal cancer; HNSC, head-neck squamous cell carcinoma; COAD, colon adenocarcinoma; READ, rectum adenocarcinoma; BRCA, breast invasive carcinoma; Ca, cancer.

**Table S1.** Univariate and multivariate analyses of factors correlated with overall survival of NPC patients

| Variables | Univariate analysis | | | | Multivariate analysis | | | |
| --- | --- | --- | --- | --- | --- | --- | --- | --- |
|  | HR | 95%CI | | *p* value | HR | 95%CI | | *p* value |
|  |  | Lower limits | Upper limits |  |  | Lower limits | Upper limits |  |
| Expression | 0.309 | 0.1 | 0.962 | 0.0426 | 0.4 | 0.11 | 1.44 | 0.16 |
| Sex | 1.182 | 0.385 | 3.625 | 0.77 |  |  |  |  |
| age | 0.542 | 0.2 | 1.467 | 0.228 |  |  |  |  |
| Tumor size | 1.764 | 0.107 | 28.976 | 0.691 |  |  |  |  |
| T | 14.742 | 3.366 | 64.567 | 0.000356 | 4.48 | 0.59 | 34.14 | 0.148 |
| N | 6.701 | 0.889 | 50.542 | 0.065 |  |  |  |  |
| M | 106.499 | 6.661 | 1702.664 | 0.000964 | 21.19 | 1.13 | 395.78 | 0.0409 |
| TNM | 19.929 | 2.642 | 150.341 | 0.00371 | 5.24 | 0.33 | 83.74 | 0.242 |

**Table S2. Primers used in this study**

| **Usage** | **Primer Name** | **Primer sequences (5’-3’)** |
| --- | --- | --- |
| PCR | *RINES-*F | CATAGTCAAGAGGAAACAAG |
|  | *RINES-*R | TAGGCAGCTTATCCATTCTGG |
|  | *RINES-*IntR | GTGAGAGACAAAGAGTCATGAC |
|  | *GAPDH-*33 | GATGACCTTGCCCACAGCCT |
|  | *GAPDH-*55 | ATCTCTGCCCCCTCTGCTGA |
| MSP | *RINES-*m1 | GGTCGGGTTTTATTTAGTCGC |
|  | *RINES-*m2 | AACCCGCCGAACGAAAAACG |
|  | *RINES-*u1 | GGTTGGGTTTTATTTAGTTGT |
|  | *RINES-*u2 | AACCCACCAAACAAAAAACA |
| BGS | *RINES-*BGS1 | GAGTTTTTAGAGTTTGGTTGT |
|  | *RINES-*BGS2 | AATTCCAACCTCCAAATAAAC |

**Table S3. Antibodies used in this study**

| **Antibodies** | **Source** | **Identifier** |
| --- | --- | --- |
| anti-mouse IgG-Alexa Fluor 488-F(ab')2 | ThermoFisher | A-11059 |
| anti-mouse IgG-HRP | ThermoFisher | 31430 |
| anti-rabbit IgG-HRP | ThermoFisher | 31460 |
| α-tubulin | Lab Vision | MS-581 |
| ABCG2 | Santa Cruz | sc-377176 |
| Aurora A | Millipore | 07-648 |
| cleaved caspase-3 | Cell Signaling | 9661 |
| cleaved PARP | Cell Signaling | 9541 |
| c-Myc | Cell Signaling | 5605 |
| E-Cadherin | Cell Signaling | 4065 |
| Flag | Sigma-Aldrich | F3165 |
| GAPDH | Millipore | MAB374 |
| GFP | Sigma-Aldrich | G1546 |
| His | Santa Cruz | sc-8036 |
| KIAA0495/SP0495 | Origene | TA503634 |
| MDM2 | Santa Cruz | sc-13161 |
| Nanog | Signaling | 4903 |
| OCT4 | Proteintech | 60242-1-Ig |
| PCNA | Sigma-Aldrich | MAB424 |
| RINES/RNF180 | OriGene | AP53686PU-N |
| RINES/RNF180 | Sigma-Aldrich | HPA006897 |
| SOX2 | Cell Signaling | 3579 |
| STAT3 | Cell Signaling | 12640 |
| Streptavidin HRP | ThermoFisher | SNN1004 |
| Ubiquitin | Cell Signaling | 20326 |
| Vimentin | Sigma-Aldrich | V6630 |

**List of abbreviations**

Abbreviation Definition

AFP Alpha-fetoprotein

ATCC American Type Culture Collection

Aza 5'-aza-2'-deoxycytidine

BGS Bisulfite genome sequencing

BRCA Breast cancer gene

CA125 Cancer antigen 125

CA199 Carbohydrate antigen 199

CA724 Cancer antigen 724

CEA Carcinoembryonic antigen

CHX Cycloheximide

COAD Colon adenocarcinoma

CRC Colorectal cancer

DKO Double knockout (DNMT1 and DNMT3B)

DNMT DNA methyltransferase

DNMTi DNA methyltransferase inhibitor

DSMZ German Collection of Microorganisms and Cell Cultures

DUB Deubiquitinating enzyme

EMT Epithelial-mesenchymal transition

ER Endoplasmic reticulum

ERAD ER-associated degradation

ESCA Esophageal carcinoma

ESCC Esophageal squamous cell carcinoma

FBS Fetal bovine serum

FPKM Fragments per kilobase of transcript per million mapped reads

GEO Gene Expression Omnibus

GFP Green fluorescent protein

GST Glutathione S-transferase

HDAC Histone deacetylase

HMEC Human mammary epithelial cells

HNSC Head and neck squamous cell carcinoma

HRP Horseradish peroxidase

MSP Methylation-specific PCR

NPC Nasopharyngeal carcinoma

PROTAC Proteolysis-targeting chimera

READ Rectum adenocarcinoma

RING Really Interesting New Gene

TCGA The Cancer Genome Atlas

TNBC Triple-negative breast cancer

TSA Trichostatin A

TUNEL Terminal deoxynucleotidyl transferase dUTP nick end labeling

UALCAN University of Alabama at Birmingham Cancer data analysis portal

Ub Ubiquitin

UBL Ubiquitin-like

UPS Ubiquitin-proteasome system

**References:**

1. Li, L., Shu, X.S., Geng, H., Ying, J., Guo, L., Luo, J., et al., *A novel tumor suppressor encoded by a 1p36.3 lncRNA functions as a phosphoinositide-binding protein repressing AKT phosphorylation/activation and promoting autophagy.* Cell Death Differ, 2023. **30**(5): 1166–1183. DOI: 10.1038/s41418-023-01129-w.

2. Li, L., Fan, Y., Huang, X., Luo, J., Zhong, L., Shu, X.S., et al., *Tumor Suppression of Ras GTPase-Activating Protein RASA5 through Antagonizing Ras Signaling Perturbation in Carcinomas.* iScience, 2019. **21**: 1–18. DOI: 10.1016/j.isci.2019.10.007.

3. Li, L., Zhang, Y., Fan, Y., Sun, K., Su, X., Du, Z., et al., *Characterization of the nasopharyngeal carcinoma methylome identifies aberrant disruption of key signaling pathways and methylated tumor suppressor genes.* Epigenomics, 2015. **7**(2): 155–73. DOI: 10.2217/epi.14.79.

4. Cheng, Y., Geng, H., Cheng, S.H., Liang, P., Bai, Y., Li, J., et al., *KRAB zinc finger protein ZNF382 is a proapoptotic tumor suppressor that represses multiple oncogenes and is commonly silenced in multiple carcinomas.* Cancer Res, 2010. **70**(16): 6516–26. DOI: 10.1158/0008-5472.CAN-09-4566.

5. Li, L., Ying, J., Li, H., Zhang, Y., Shu, X., Fan, Y., et al., *The human cadherin 11 is a pro-apoptotic tumor suppressor modulating cell stemness through Wnt/beta-catenin signaling and silenced in common carcinomas.* Oncogene, 2012. **31**(34): 3901–12. DOI: 10.1038/onc.2011.541.

6. Qiu, G.H., Tan, L.K., Loh, K.S., Lim, C.Y., Srivastava, G., Tsai, S.T., et al., *The candidate tumor suppressor gene BLU, located at the commonly deleted region 3p21.3, is an E2F-regulated, stress-responsive gene and inactivated by both epigenetic and genetic mechanisms in nasopharyngeal carcinoma.* Oncogene, 2004. **23**(27): 4793–806. DOI: 10.1038/sj.onc.1207632.

7. Ying, J., Li, H., Seng, T.J., Langford, C., Srivastava, G., Tsao, S.W., et al., *Functional epigenetics identifies a protocadherin PCDH10 as a candidate tumor suppressor for nasopharyngeal, esophageal and multiple other carcinomas with frequent methylation.* Oncogene, 2006. **25**(7): 1070–80. DOI: 10.1038/sj.onc.1209154.

8. Li, L., Ying, J., Tong, X., Zhong, L., Su, X., Xiang, T., et al., *Epigenetic identification of receptor tyrosine kinase-like orphan receptor 2 as a functional tumor suppressor inhibiting beta-catenin and AKT signaling but frequently methylated in common carcinomas.* Cell Mol Life Sci, 2014. **71**(11): 2179–92. DOI: 10.1007/s00018-013-1485-z.
