# Supplementary material for: Loss of E3 Ubiquitin Ligase RINES via CpG Methylation Relieves Suppression of STAT3 and MYC, Facilitating Multiple Tumorigeneses
Source: Adv Sci (Weinh). 2026 Jul 14:e23684. Online ahead of print. doi: 10.1002/advs.202523684 (PMC13366374; doi:10.1002/advs.202523684)

Supplementary Information – Raw data

Figure 3A, B

Uncropped blots related to Figure 3A

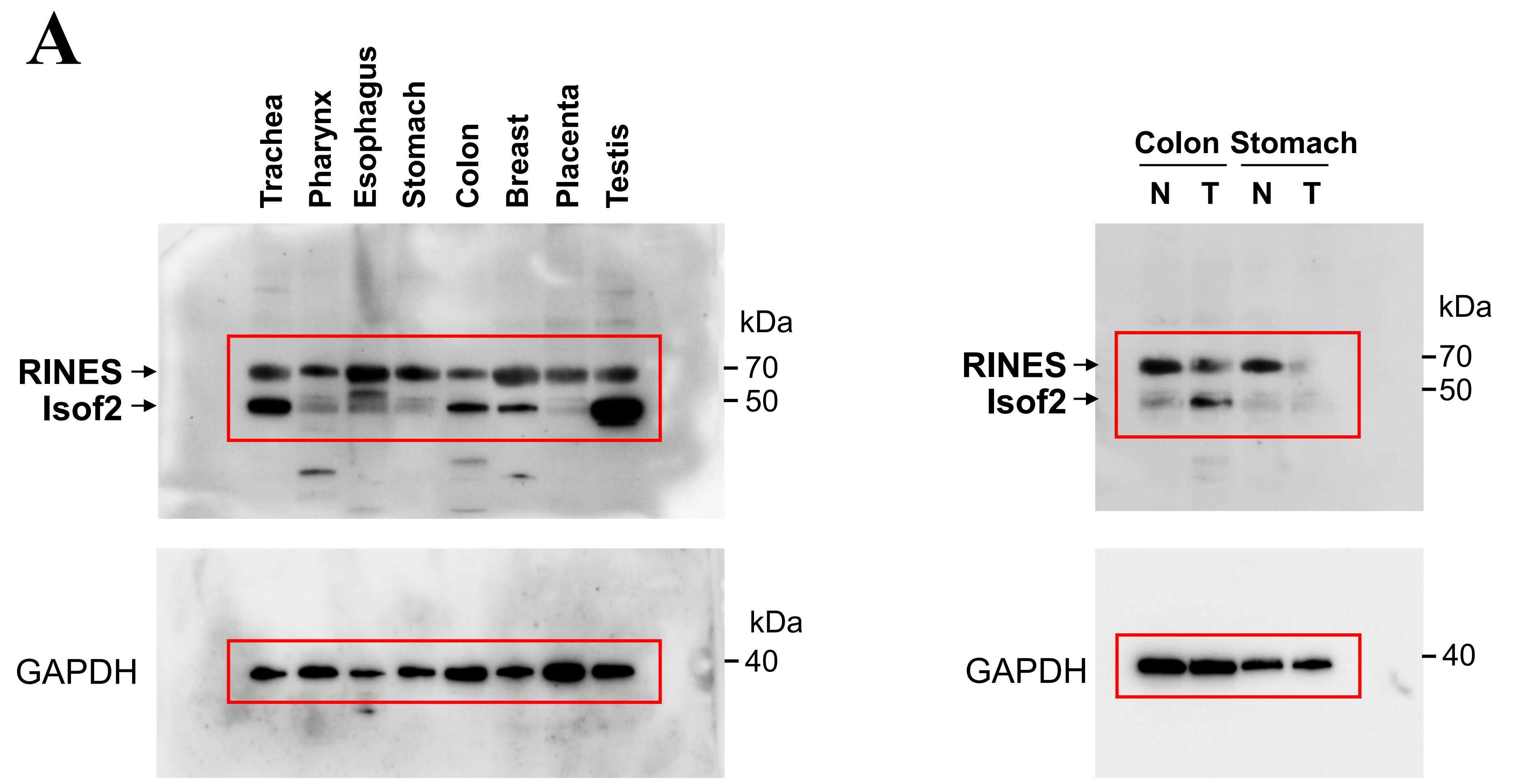

Uncropped images related to Figure 3B

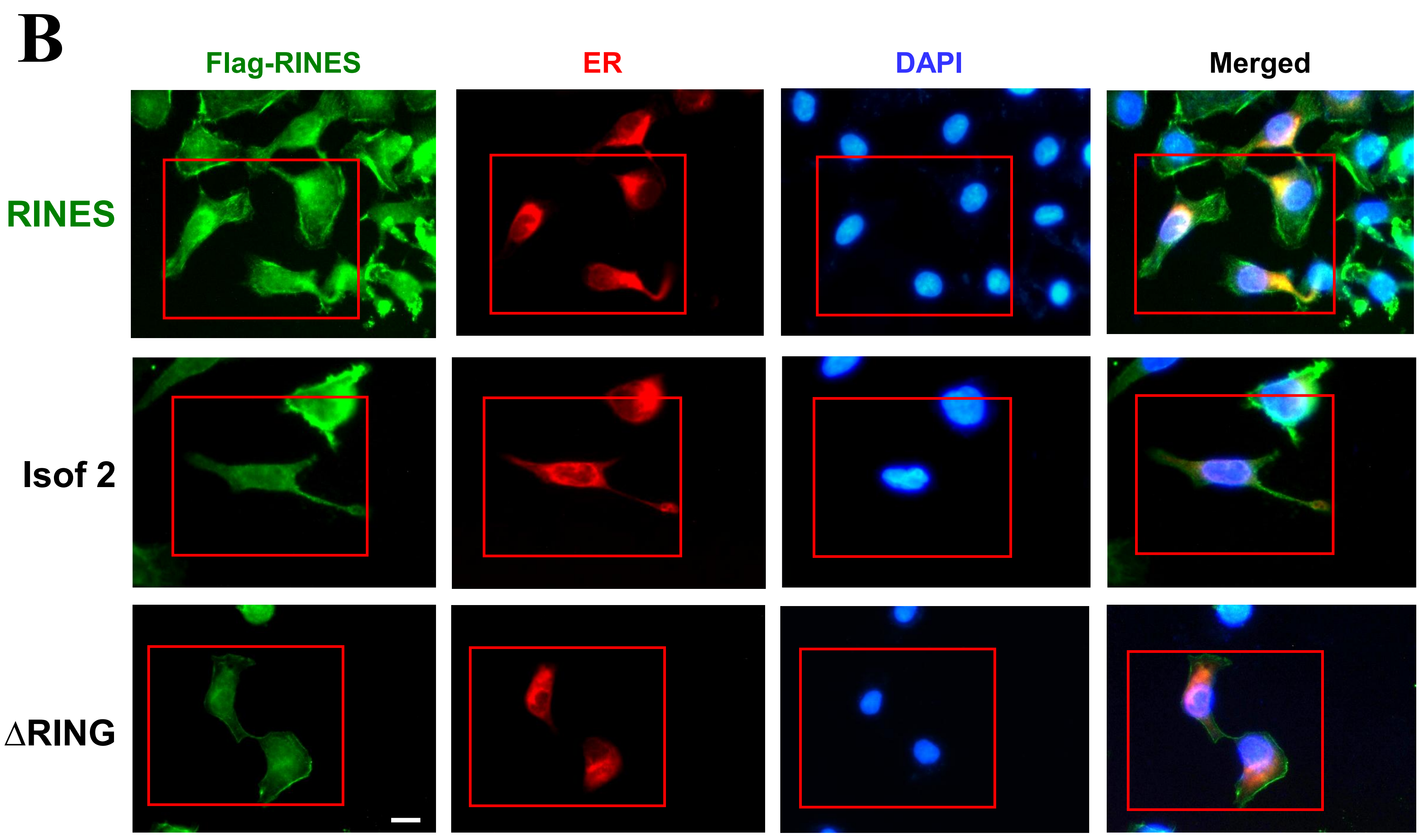

Figure 3C, I, J

Uncropped blots related to Figure 3C

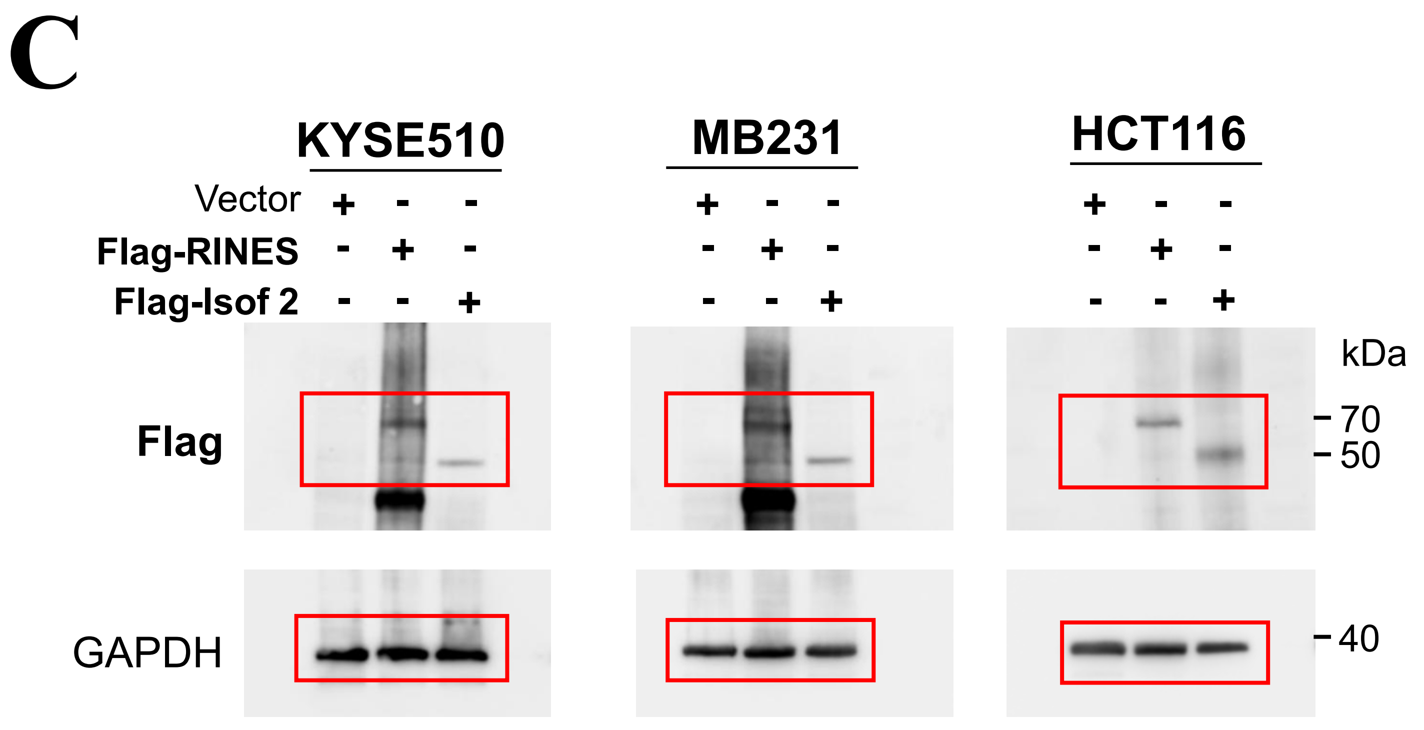

Uncropped images related to Figure 3I

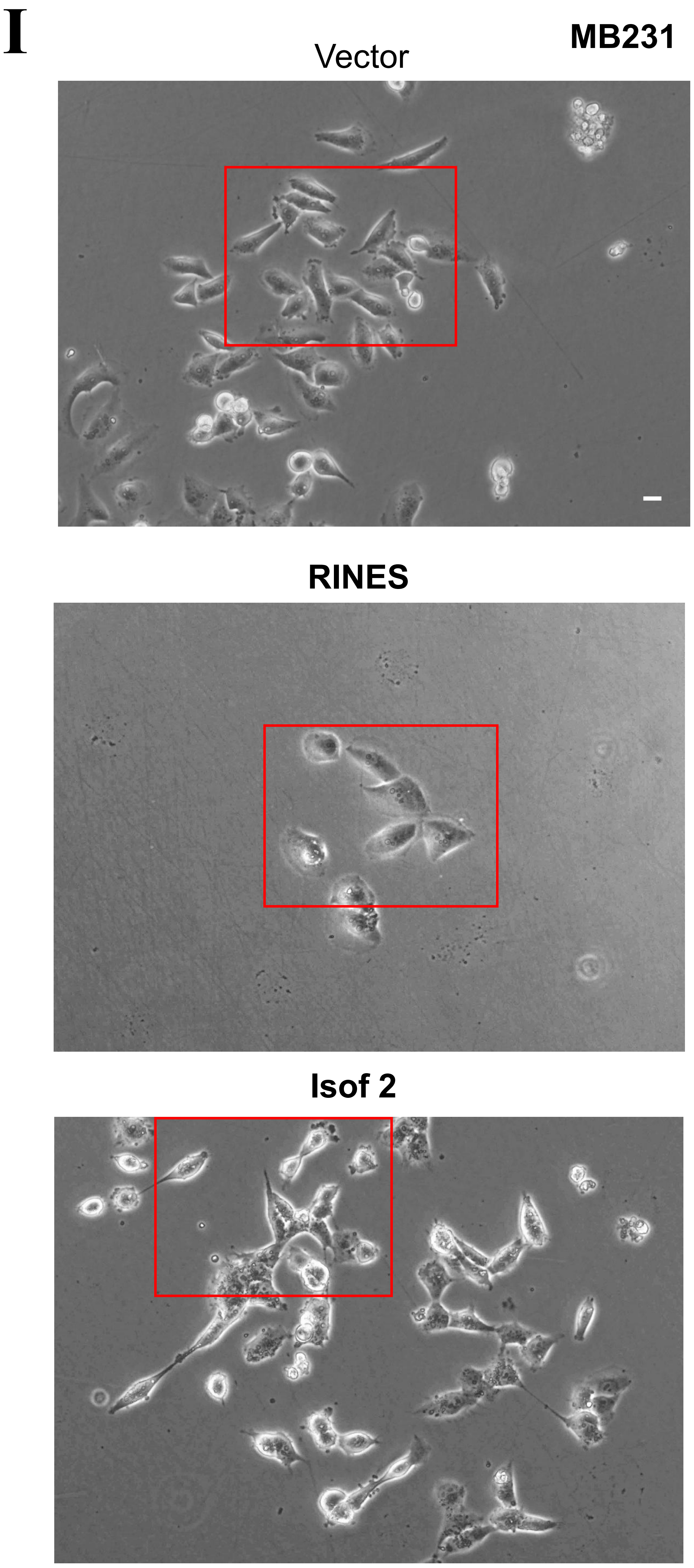

Uncropped blots related to Figure 3J

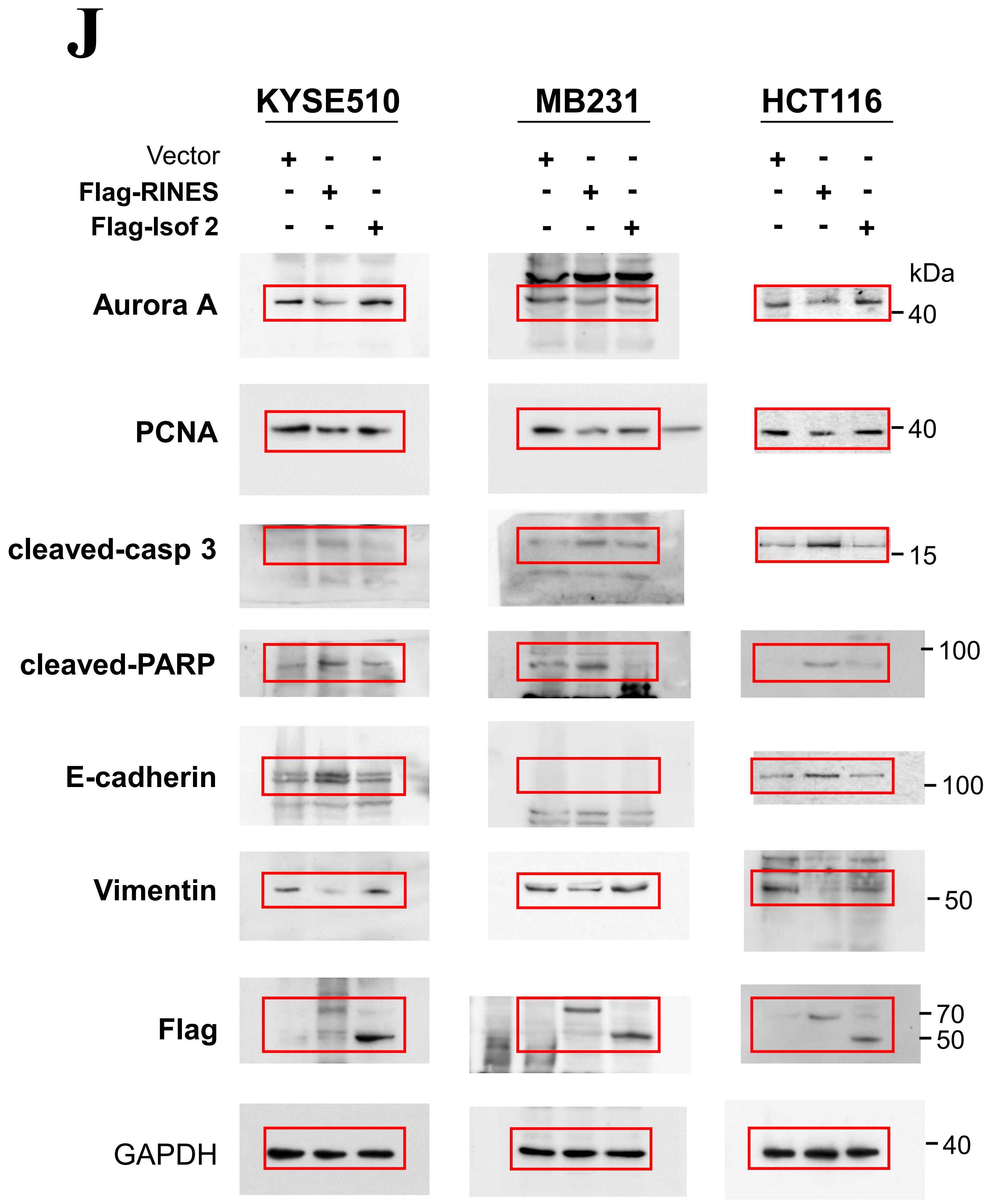

Figure 4B, C, D

Uncropped blots related to Figure 4B

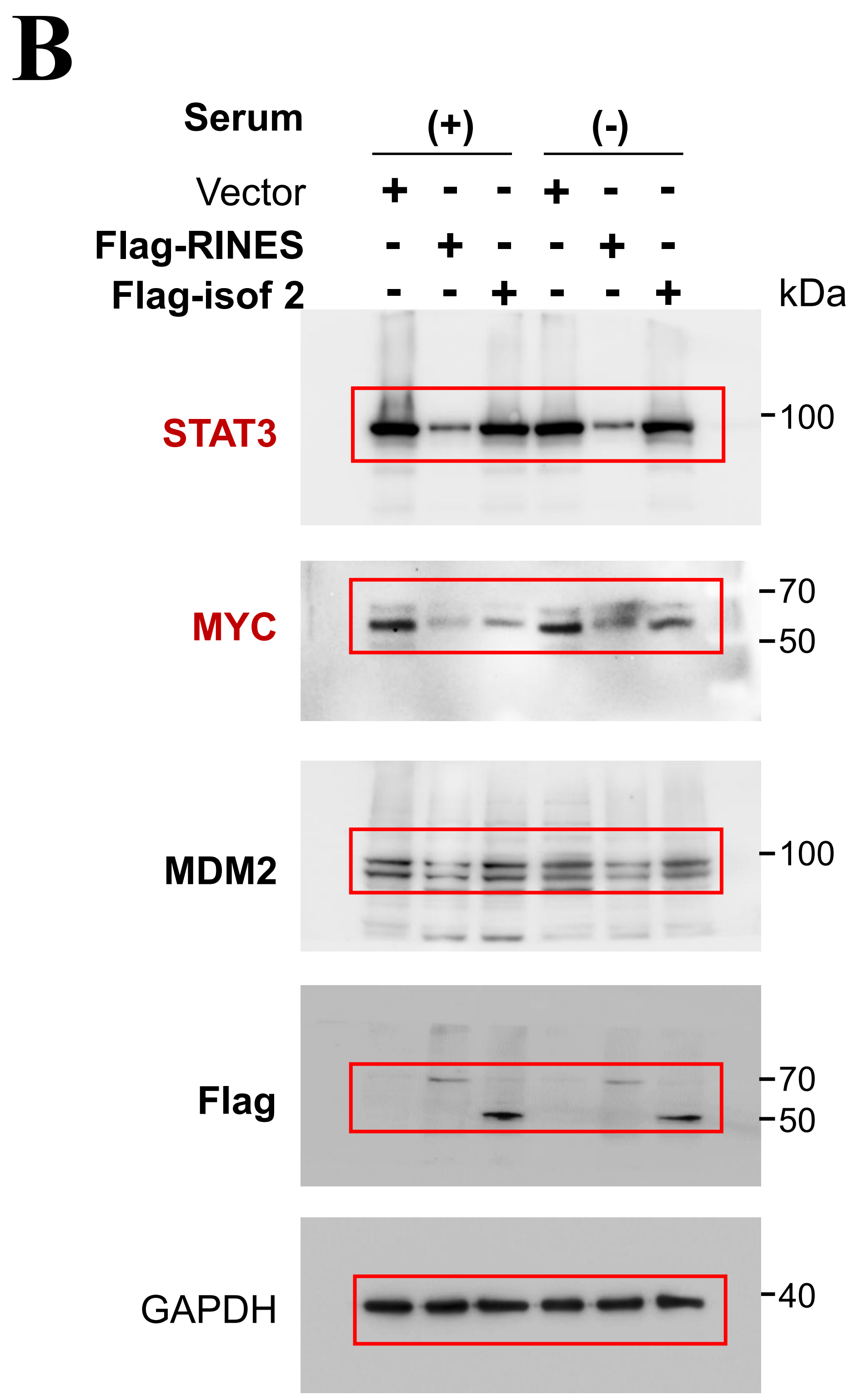

Uncropped blots related to Figure 4C

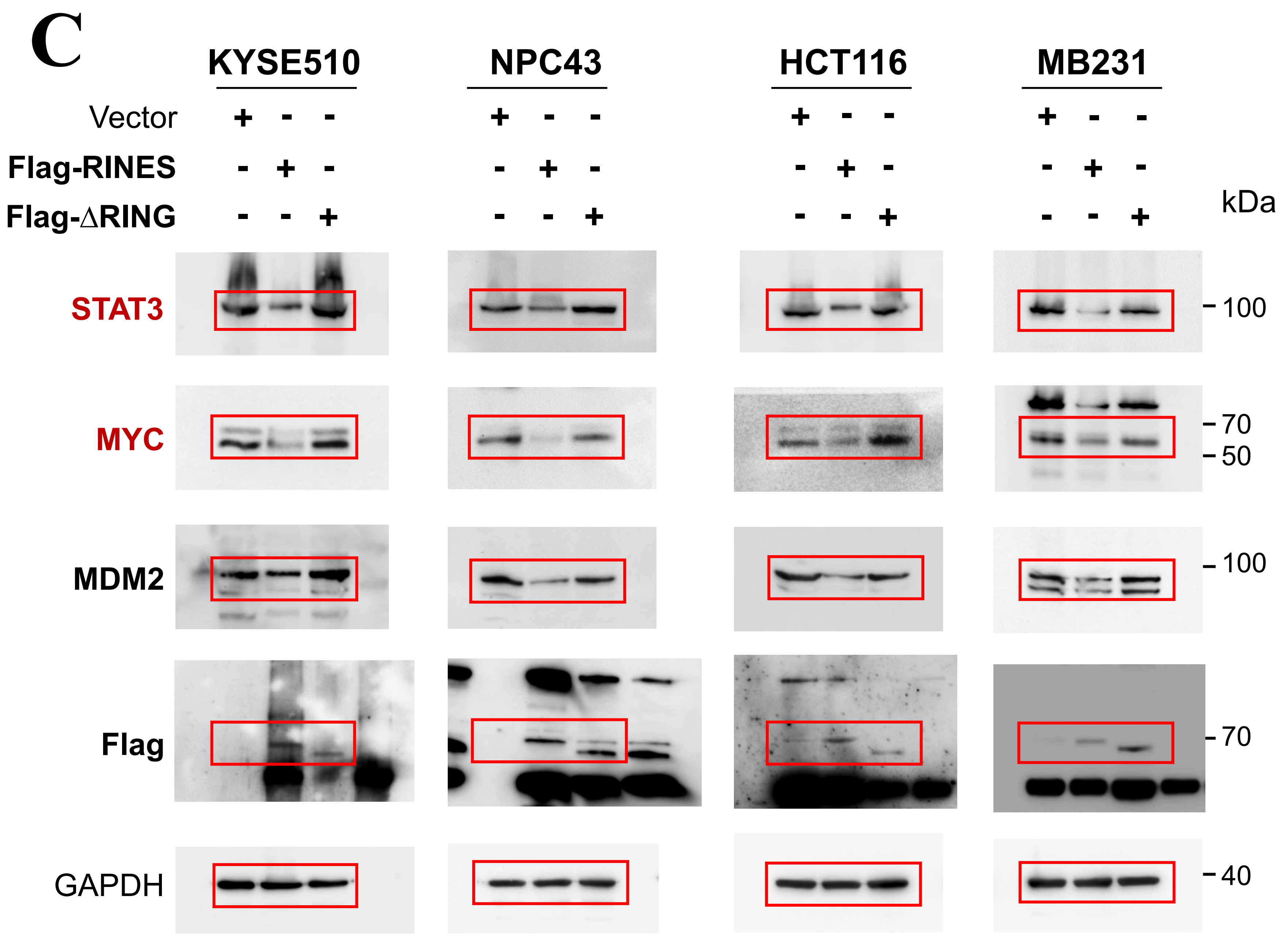

Uncropped blots related to Figure 4D

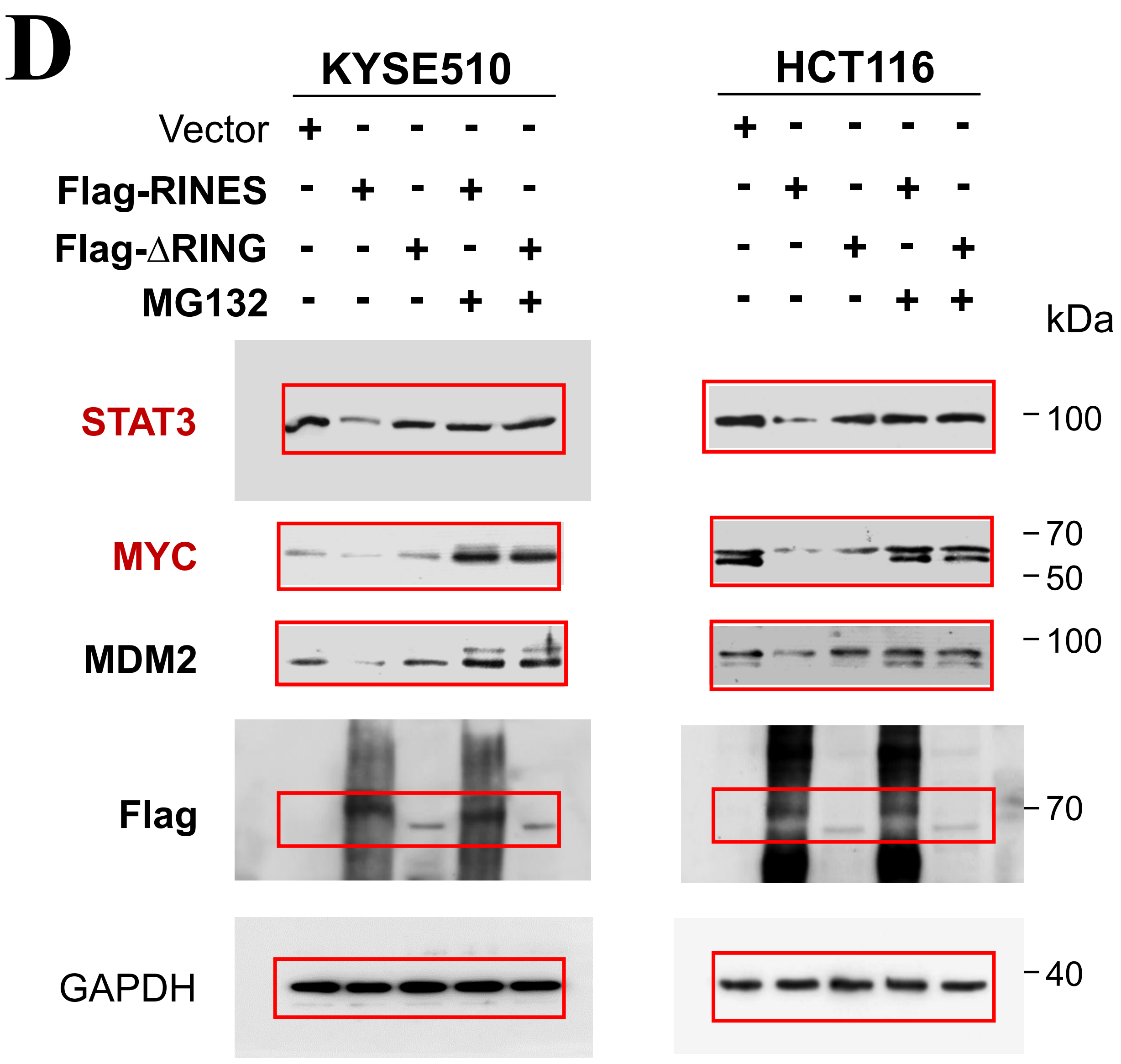

Figure 4G, H

Uncropped blots related to Figure 4G

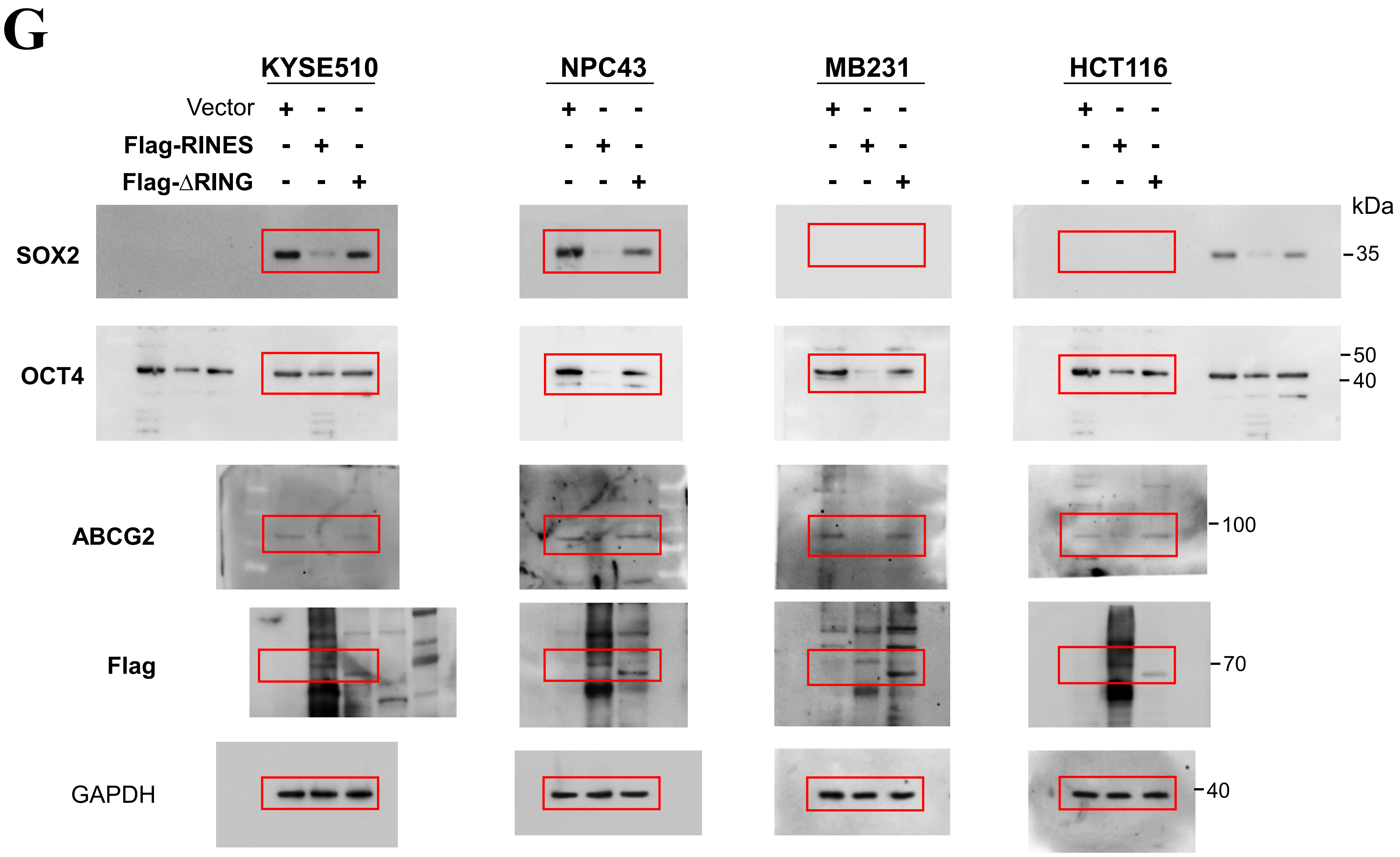

Uncropped blots related to Figure 4H

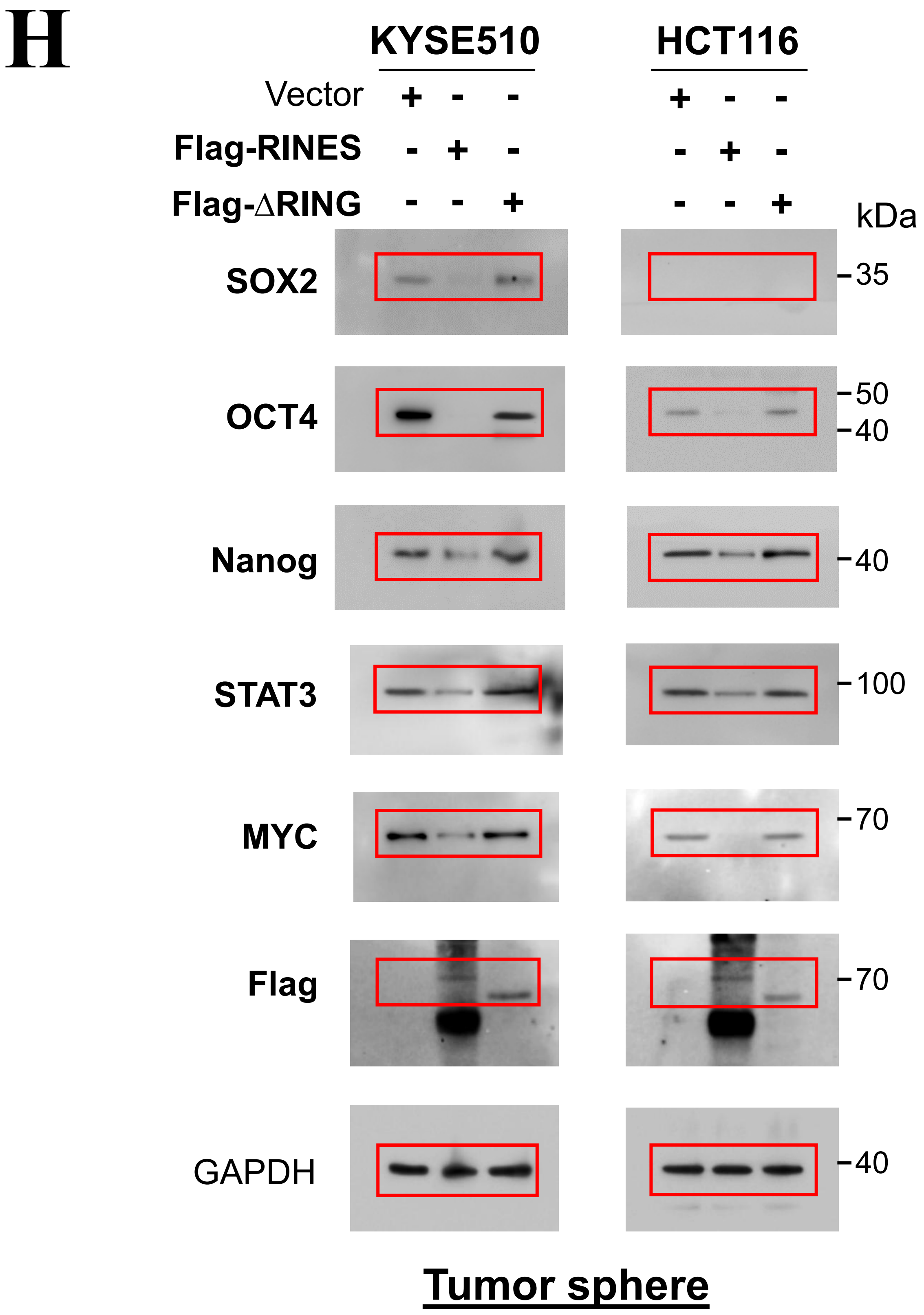

Figure 5A, B

Uncropped blots related to Figure 5A

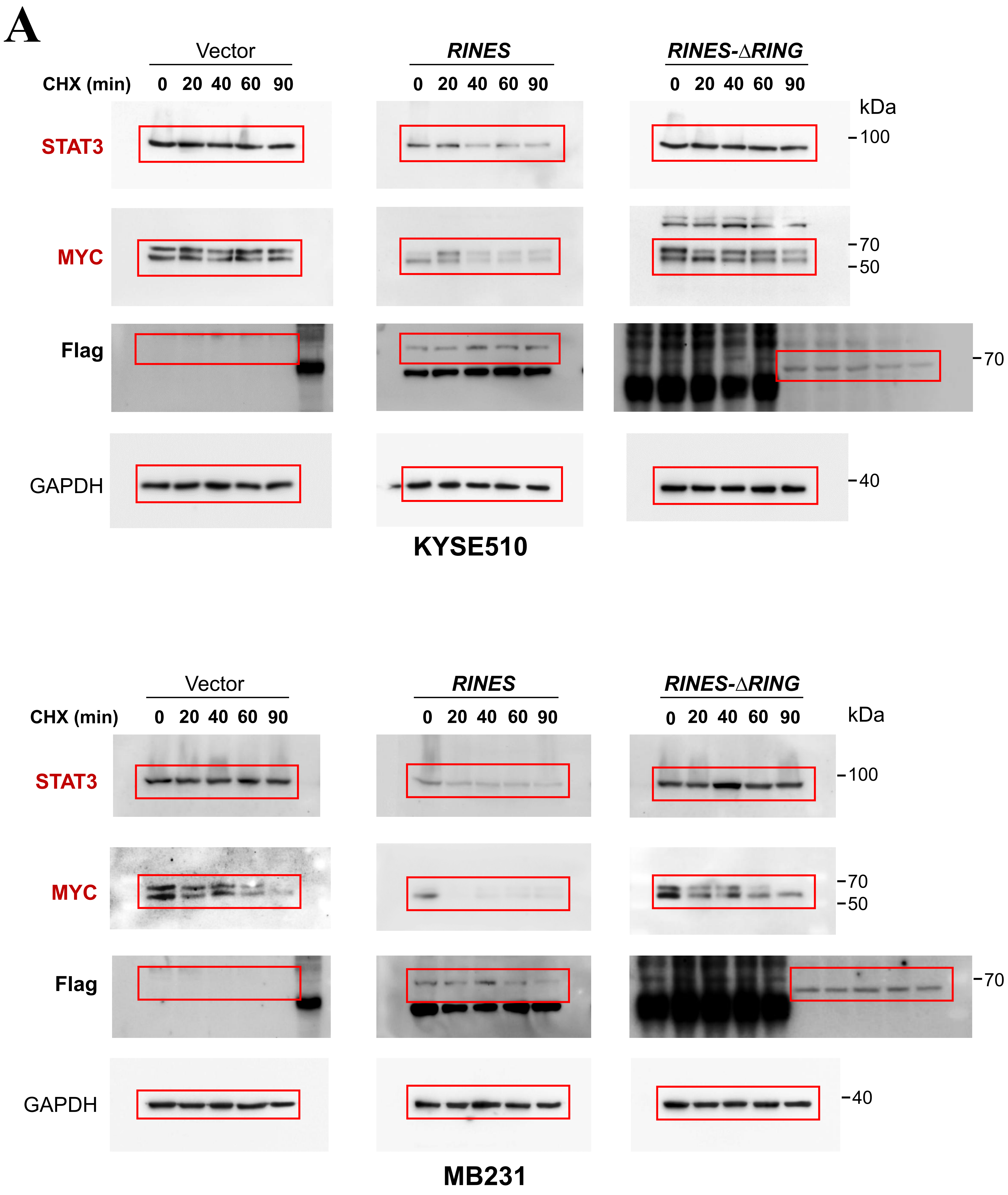

Uncropped blots related to Figure 5B

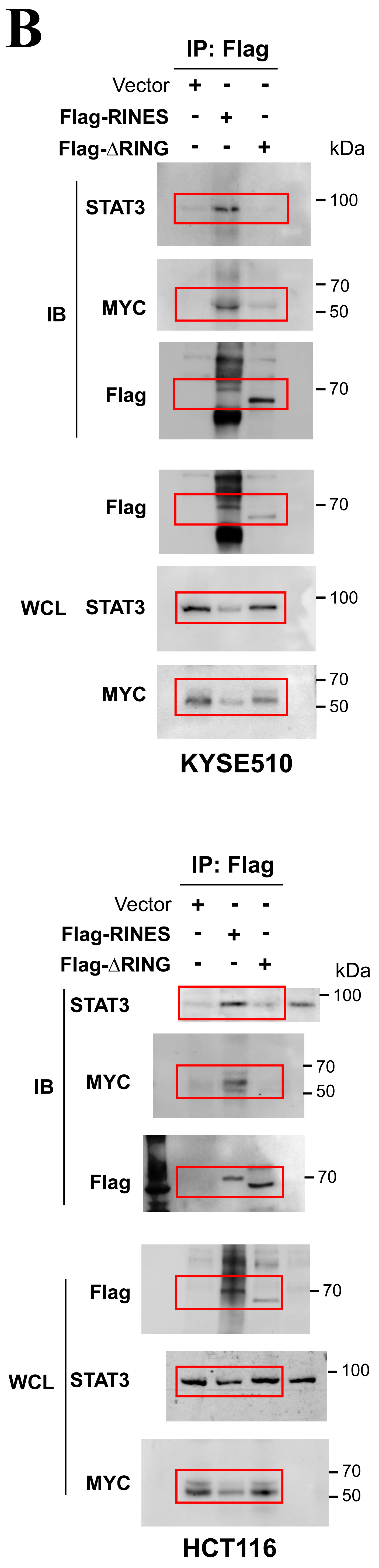

Figure 5C

Uncropped blots related to Figure 5C

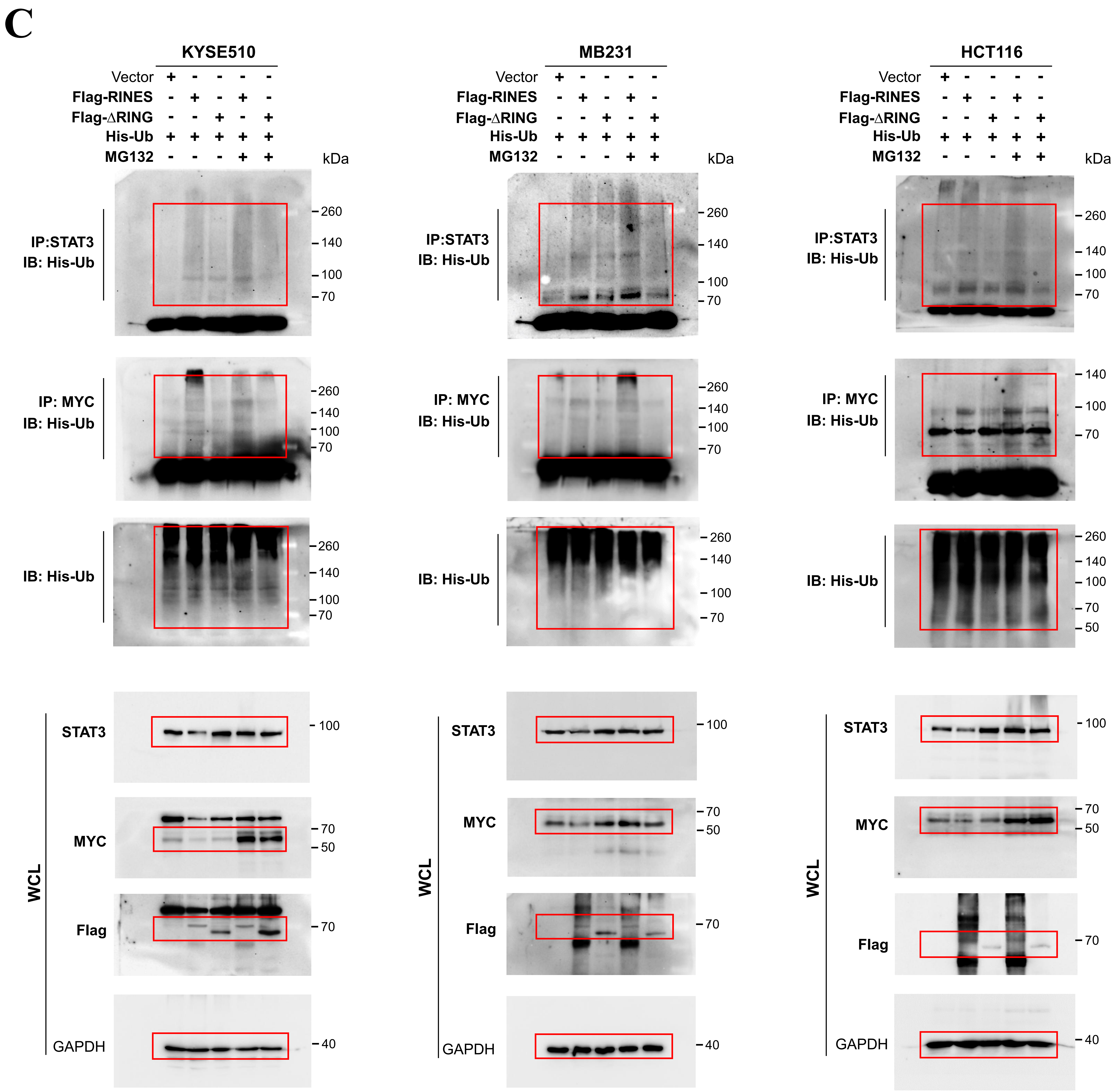

Figure 5D, E

Uncropped blots related to Figure 5D

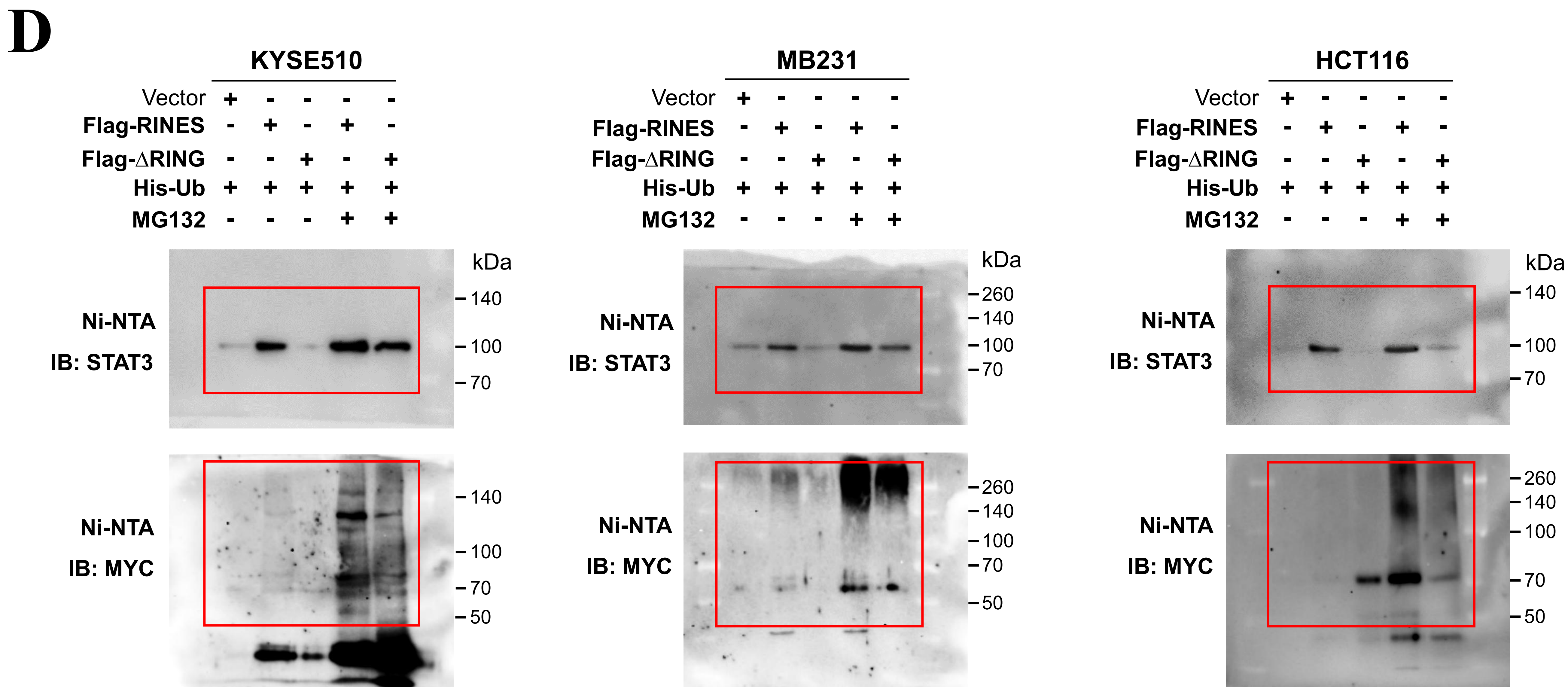

Uncropped blots related to Figure 5E

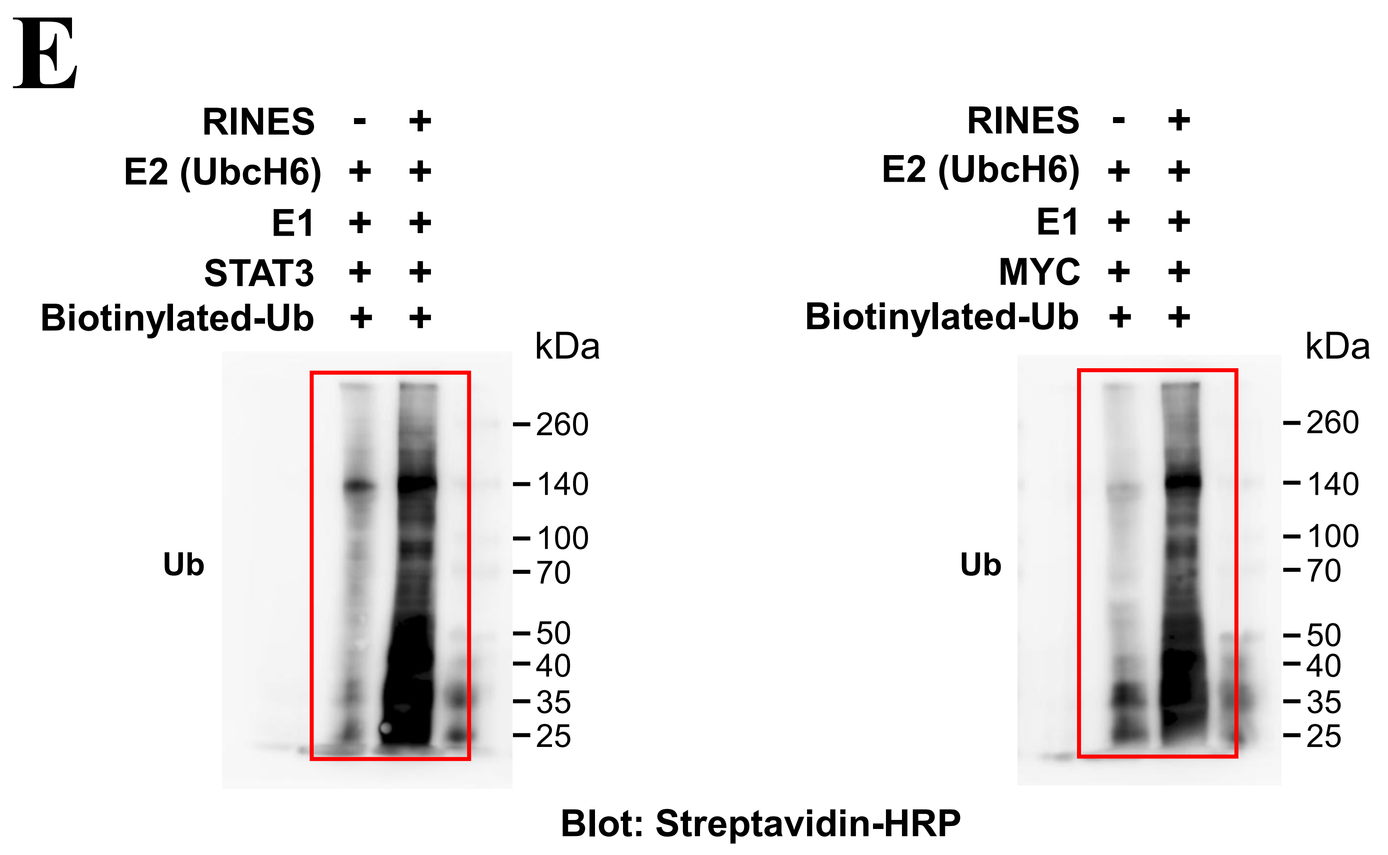

Figure 6A

Uncropped blots related to Figure 6A

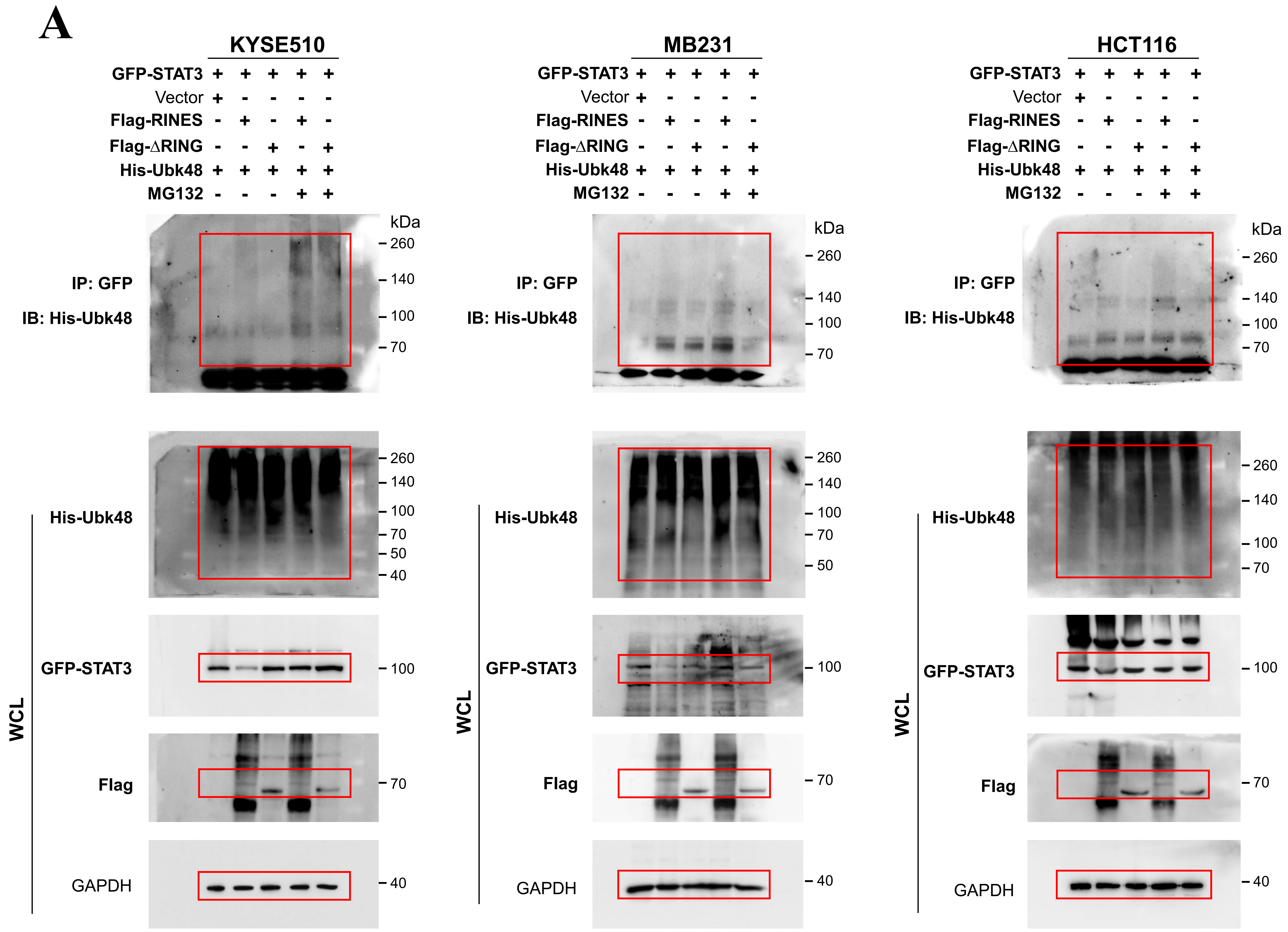

Figure 6B

Uncropped blots related to Figure 6B

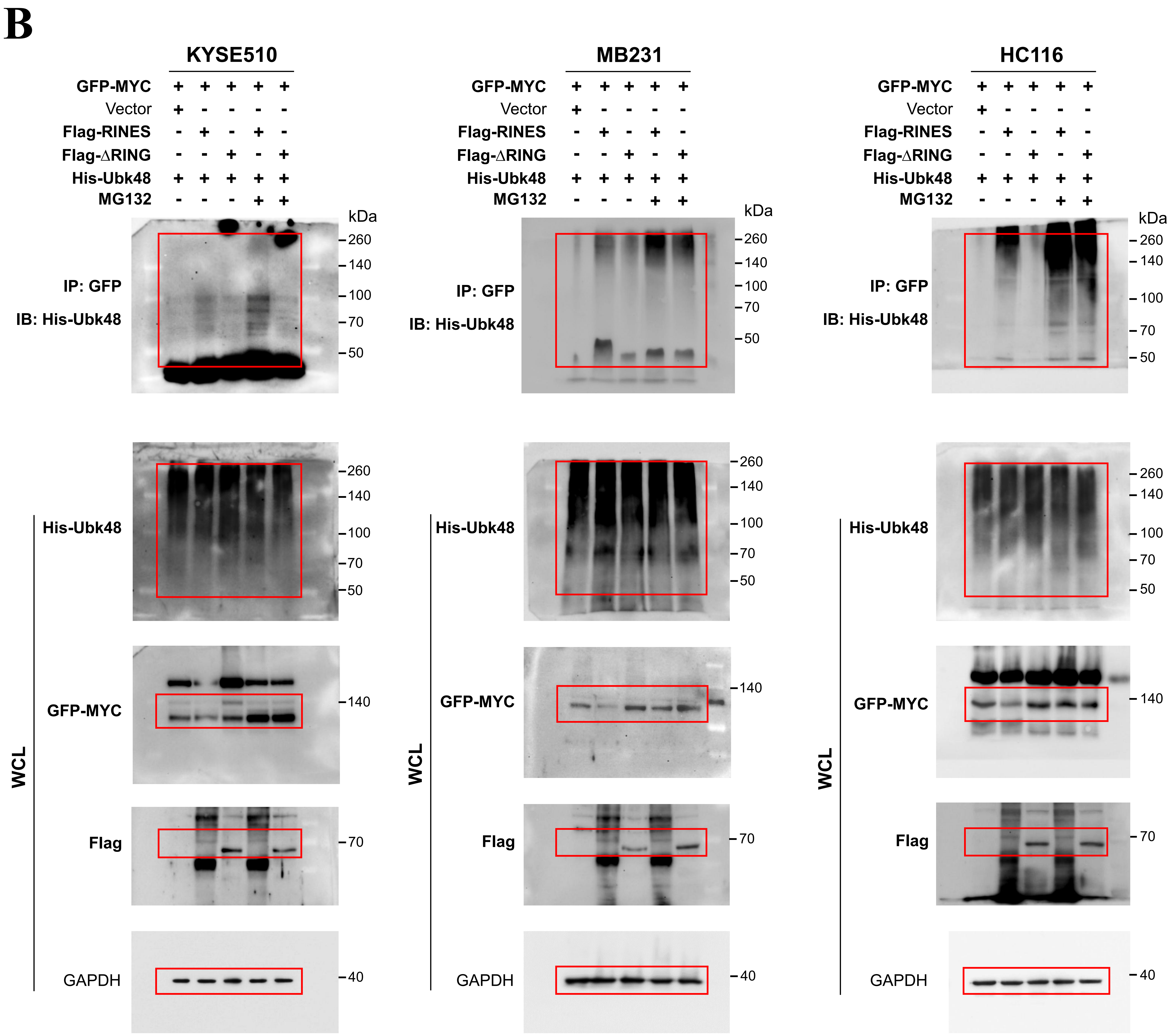

Figure 6C

Uncropped blots related to Figure 6C

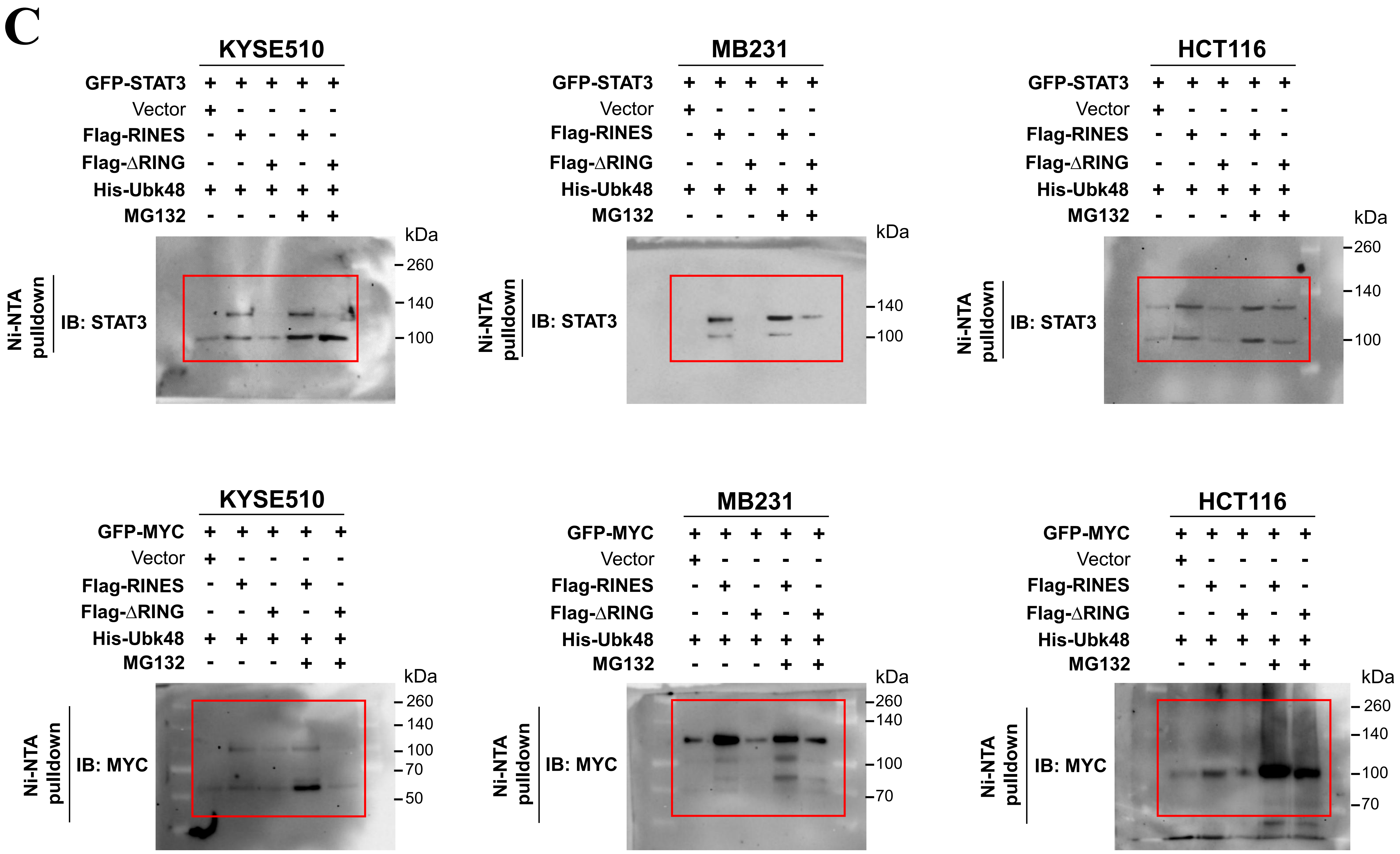

Figure 7C

Uncropped blots related to Figure 7C

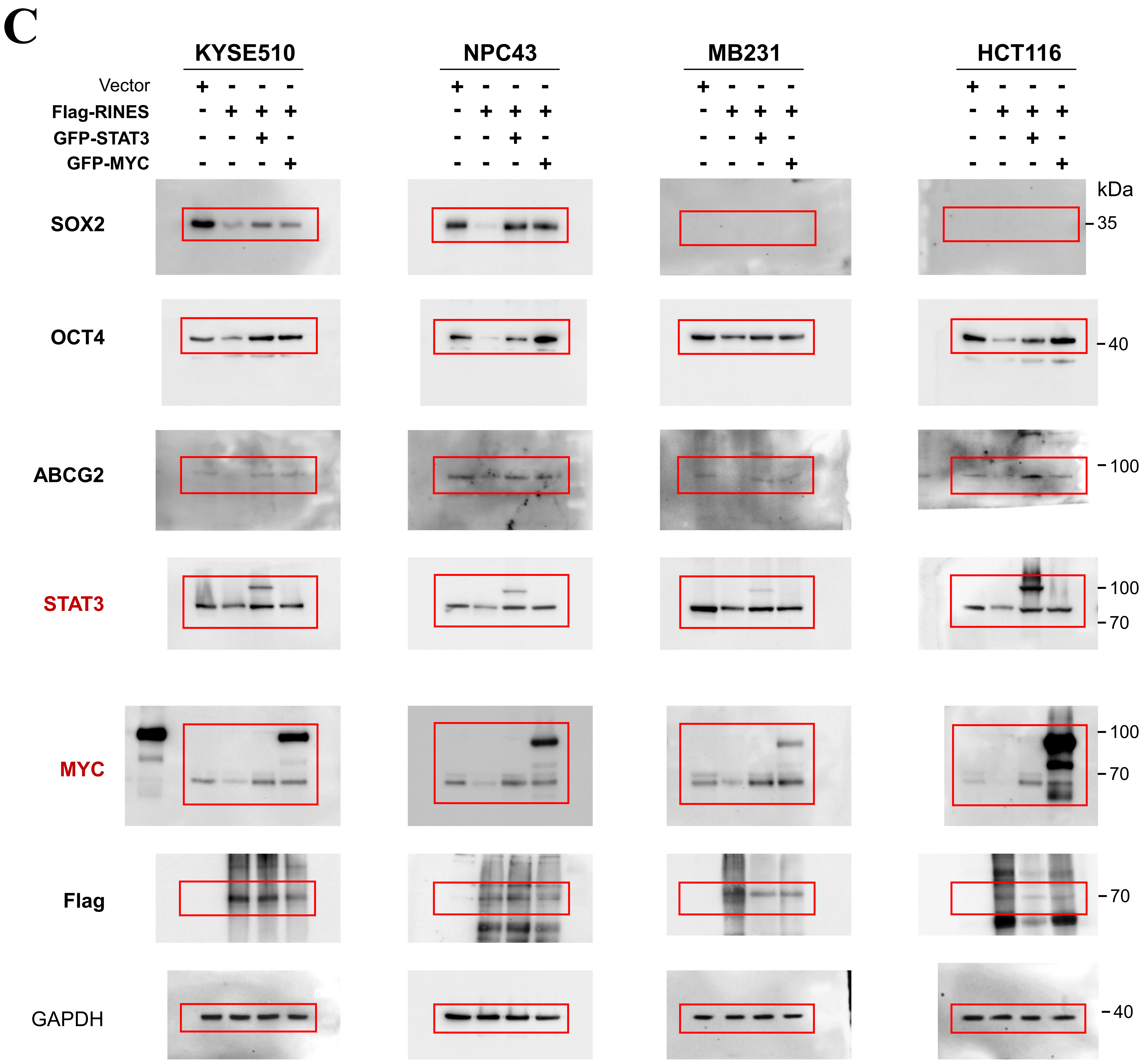

Figure 7D

Uncropped blots related to Figure 7D

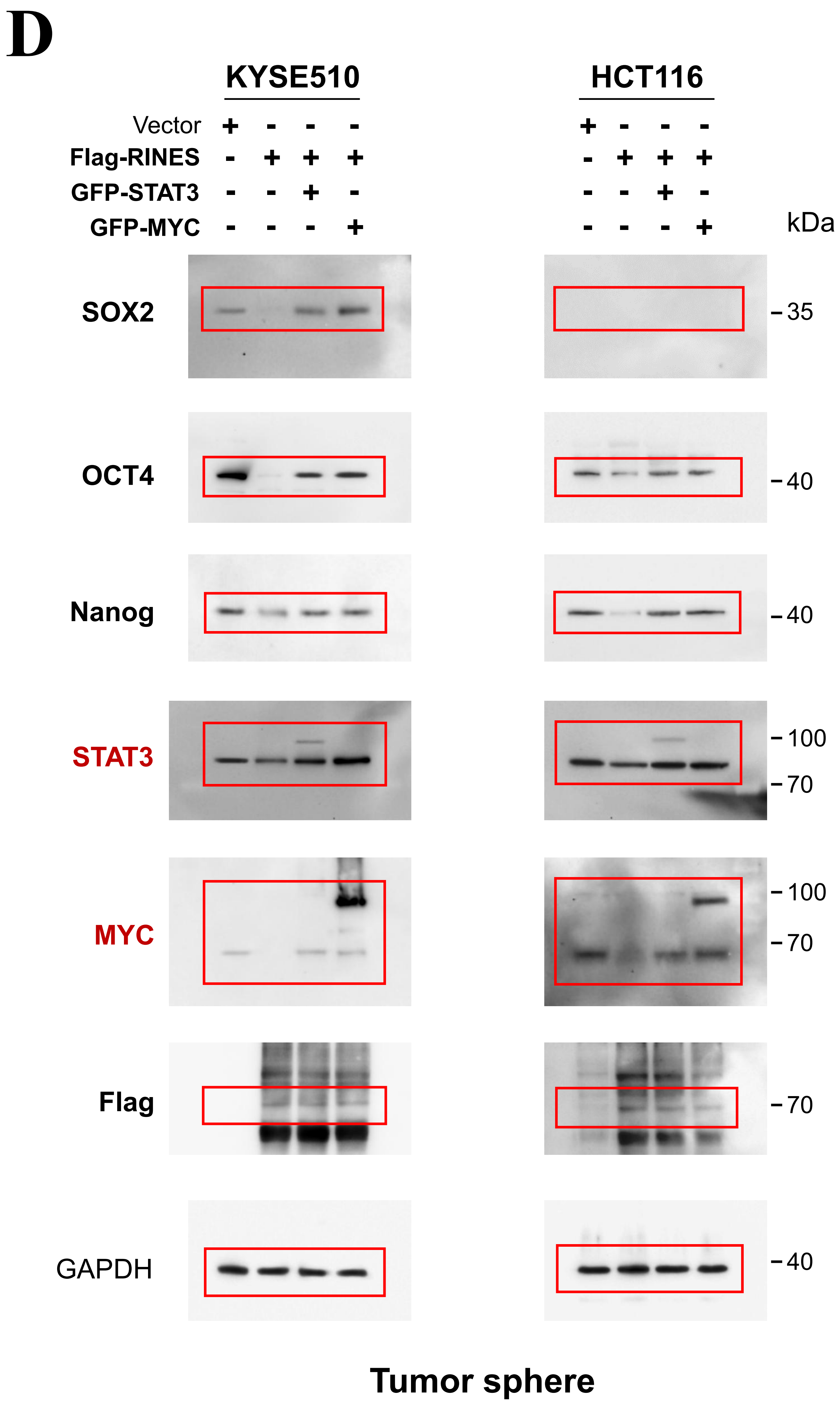

Figure 8B

Uncropped blots related to Figure 8B

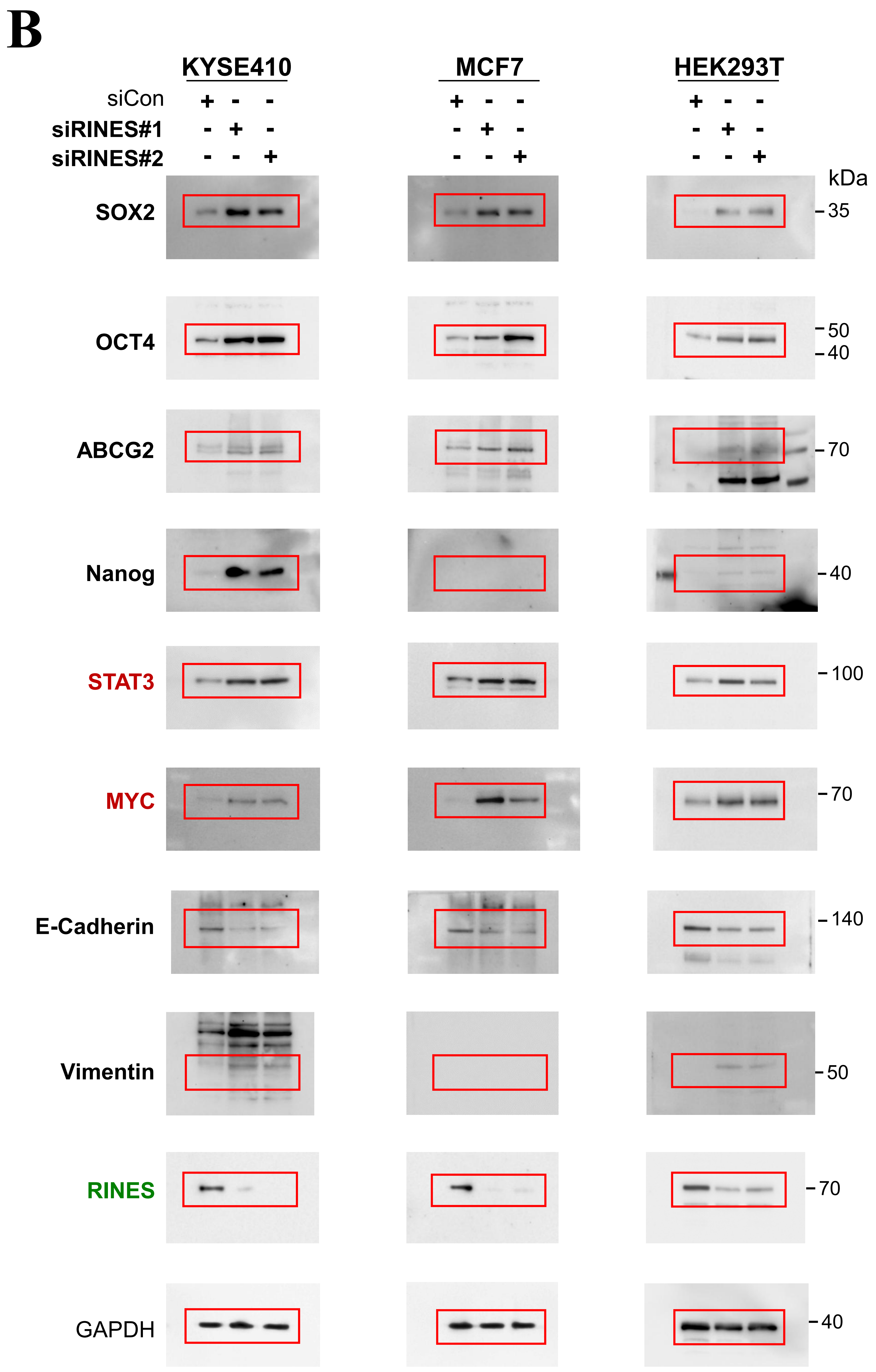

Figure 8C, D, E

Uncropped blots related to Figure 8C

C

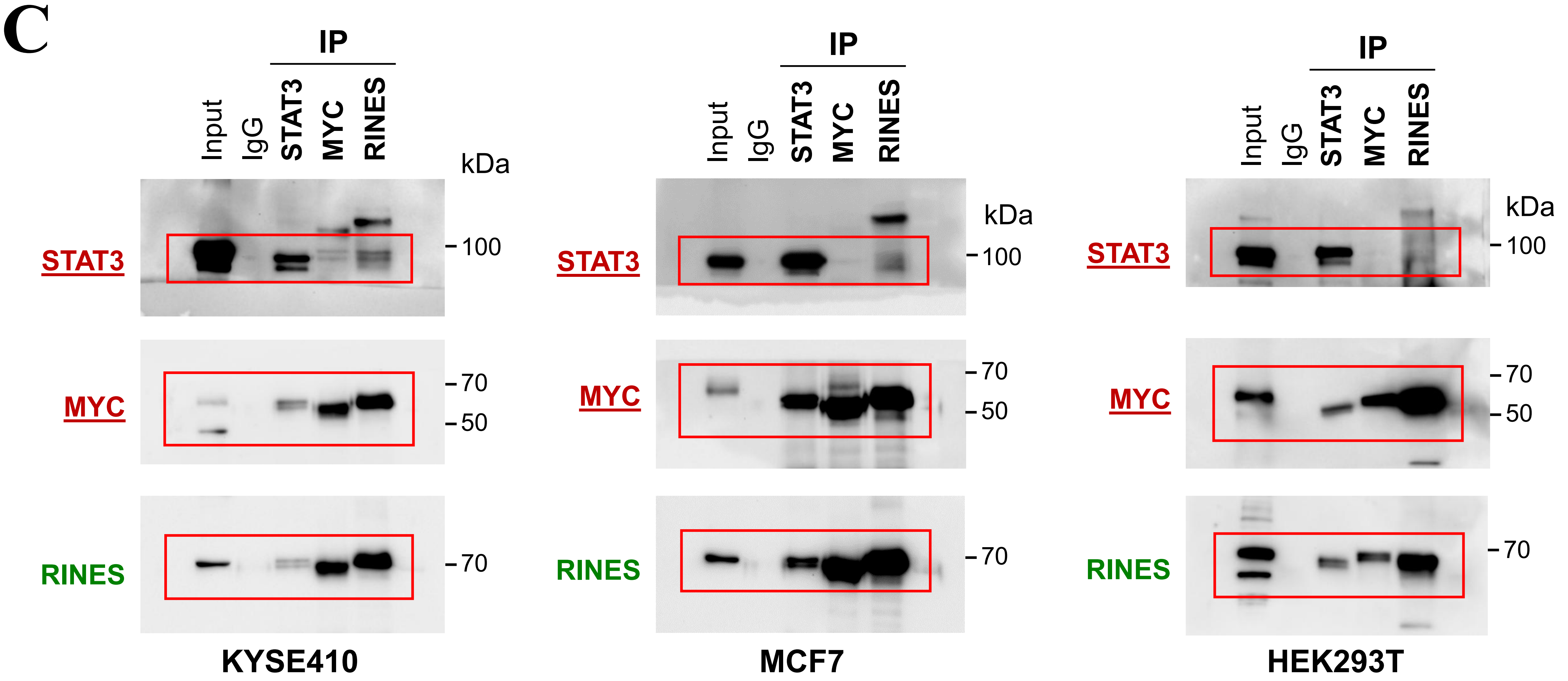

Uncropped blots related to Figure 8D

D

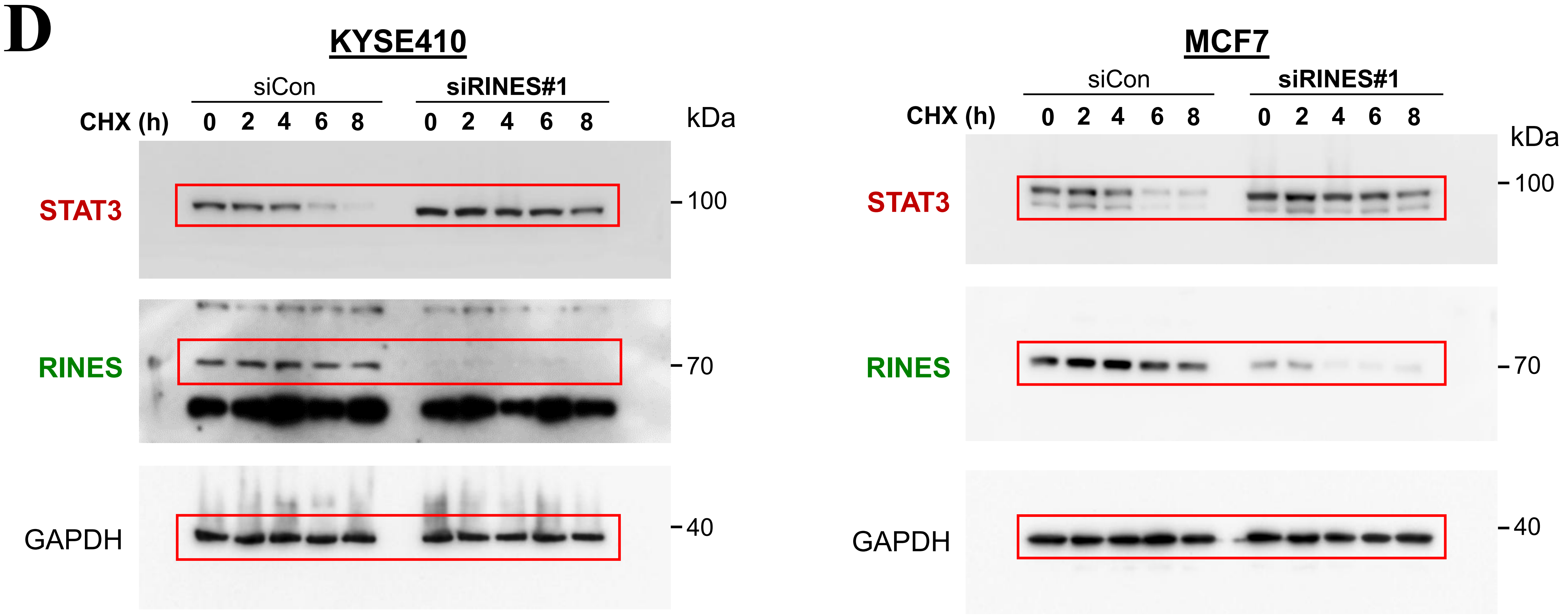

Uncropped blots related to Figure 8E

E

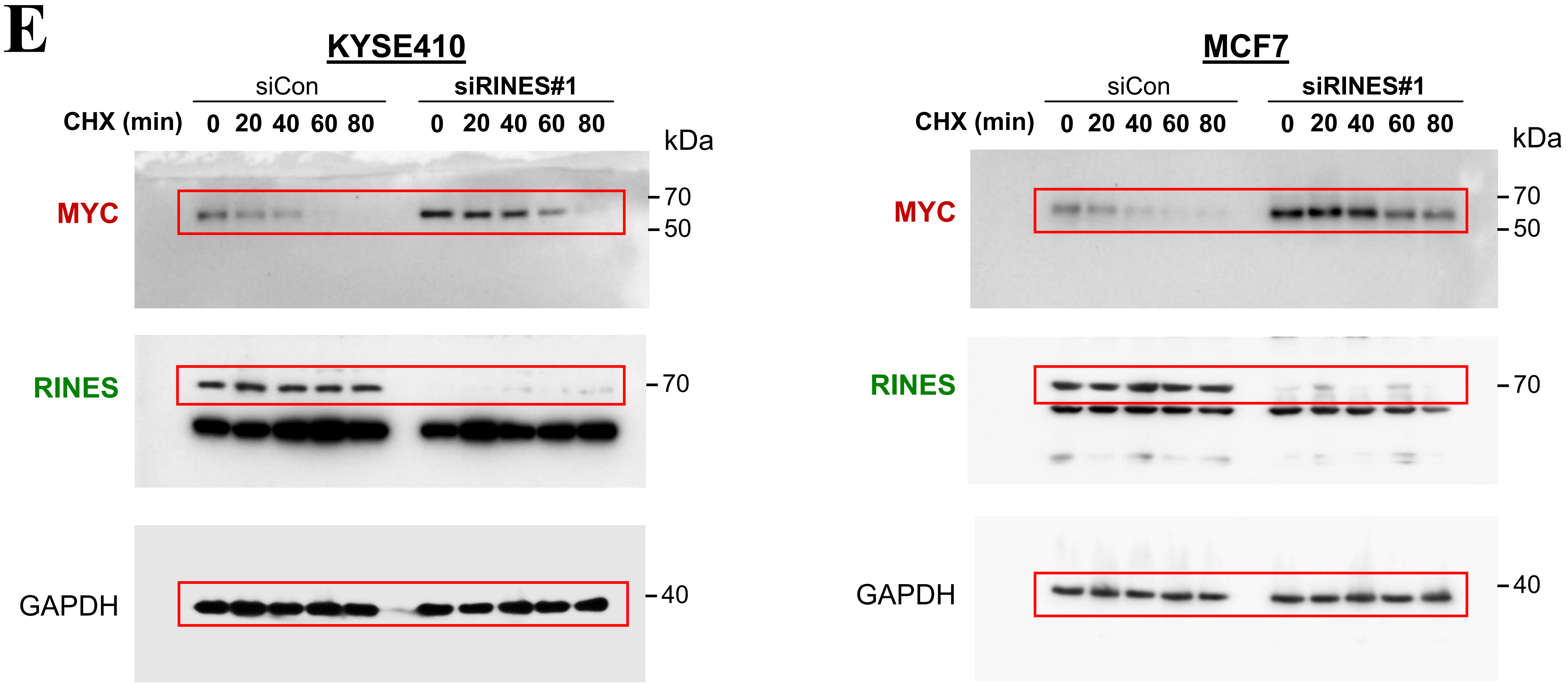

Figure 8F

Uncropped blots related to Figure 8F

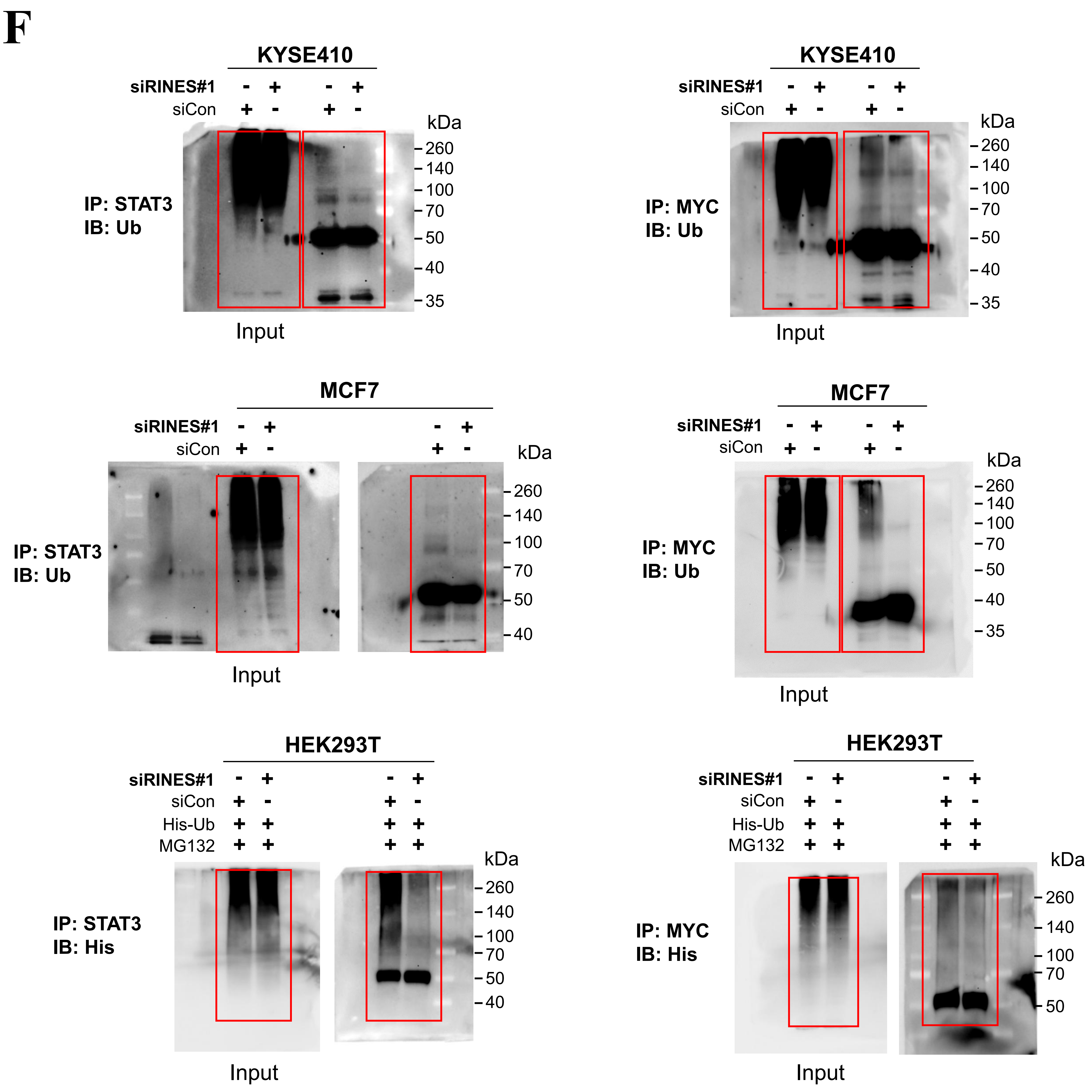

Suppl. Figure 5A

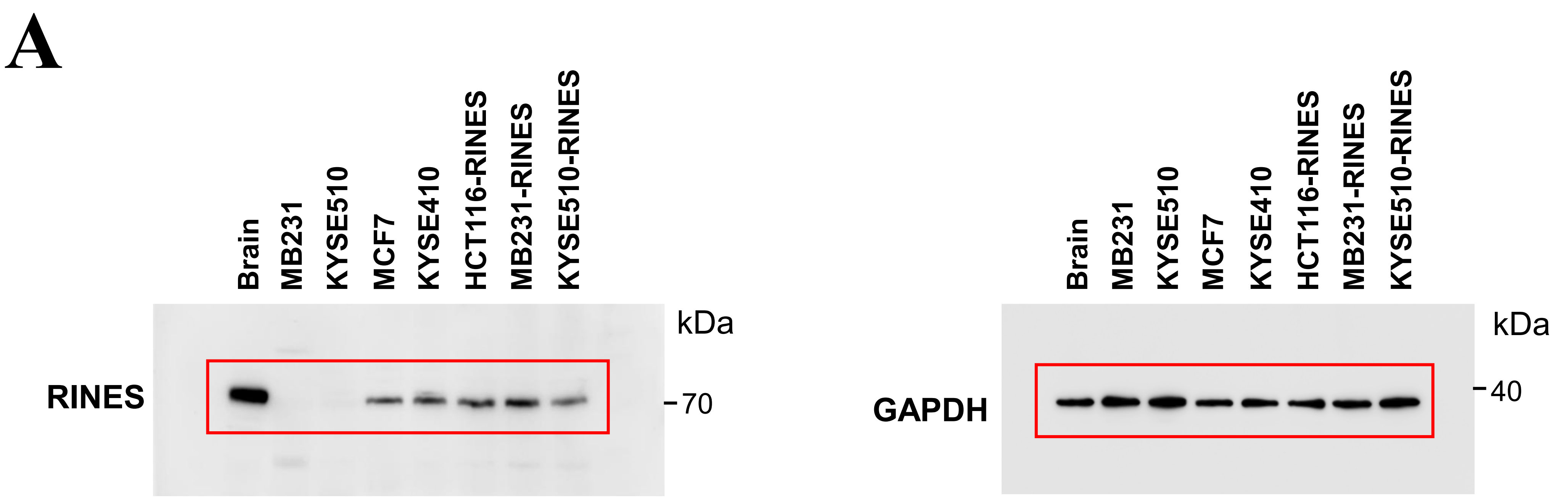

Supplement: Supplementary file 2 — Supporting File 2: advs76515‐sup‐0002‐DataFile.pdf. [file ADVS-9999-e23684-s002.pdf]
